# New Aspects of the Reaction of Thioacetamide and N-Substituted Maleimides

**DOI:** 10.3390/molecules27248800

**Published:** 2022-12-12

**Authors:** Yulia V. Aseeva, Nadezhda V. Stolpovskaya, Dmitriy Y. Vandyshev, Vladimir B. Sulimov, Mikhail A. Prezent, Mikhail E. Minyaev, Khidmet S. Shikhaliev

**Affiliations:** 1 Department of Organic Chemistry, Faculty of Chemistry, Voronezh State University, Universitetskaya Sq. 1, 394018 Voronezh, Russia; 2 Moscow Center of Fundamental and Applied Mathematics, Leninskie Gory Str., 1, 119234 Moscow, Russia; 3 Research Computing Center, Lomonosov Moscow State University, Leninskie Gory, 1, Building 4, 119234 Moscow, Russia; 4 N. D. Zelinsky Institute of Organic Chemistry, Russian Academy of Sciences, 47 Leninsky Prosp., 119991 Moscow, Russia

**Keywords:** N-arylmaleimide, N-alkylmaleimide, thioacetamide, recyclization, nucleophilic addition, Diels-Alder reaction, epithiopyrrolo[3,4-c]pyridin, pyrrolo[3,4-c]pyridin, 3,3′-thiobis(1-arylpyrrolidine-2,5-dione), 3,3′-thiobis(1-alkylpyrrolidine-2,5-dione)

## Abstract

N-Arylmaleimides are universal substrates for the synthesis of various heterocyclic compounds with a wide spectrum of biological activity. However, their reactions with thioacetamides have not been comprehensively studied. We studied the reactions of thioacetamide with N-arylmaleimides under various conditions. We established for the first time that three types of products: epithiopyrrolo[3,4-c]pyridines, pyrrolo[3,4-c]pyridines and 3,3′-thiobis(1-arylpyrrolidine-2,5-diones) can be obtained in different conditions. In all cases, two maleimide molecules are involved in the reaction. 3,3′-Thiobis(1-arylpyrrolidine-2,5-diones) are the major products when the reaction is conducted at boiling in acetic acid. When thioacetamide and N-arylmaleimide are kept in dioxane at 50 °C, epithiopyrrolo[3,4-c]pyridines can be isolated, which, when heated in dioxane, in acetic acid or in methanol in the presence of catalytic amounts of sodium methoxide, are converted into pyrrolo[3,4-c]pyridines by eliminating hydrogen sulfide. The reaction of thioacetamide and N-arylmaleimide in dioxane at boiling temperature with the portioned addition of N-arylmaleimide leads predominantly to the formation of pyrrolo[3,4-c]pyridines. The reaction of thioacetamide with N-alkylmaleimides under all the above conditions leads predominantly to the formation of the corresponding sulfides. The structure of the compounds obtained was characterized by a set of spectral analysis methods and X-ray diffraction (XRD) data.

## 1. Introduction

**Scheme 1:**
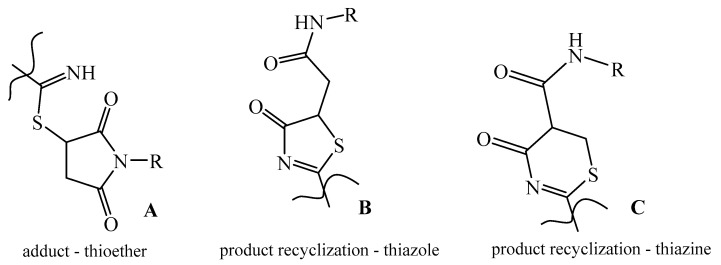
Possible products of the interaction of N-substituted maleimides with 1,3-N,S-binucleophiles.

N-substituted maleimides are promising organic substrates that are used to build a wide range of fused and linearly linked heterocyclic scaffolds with biologically active properties [1,2,3,4,5,6,7,8,9]. The reactive feature of maleic acid imides is their ability to enter into 1,3-dipolar addition reactions [8,9,10,11,12] and into recyclization reactions under the action of binucleophilic reagents—in particular, 1,3-S,N-binucleophiles [1,2,3,4,5,6,7,13,14,15,16,17,18,19,20,21,22,23]. In such reactions, the addition of a more nucleophilic sulfur atom to the activated double bond of N-substituted maleimide usually occurs at the first stage, and the recyclization of the succinimide ring under the action of the nucleophilic nitrogen atom occurs at the second stage [19,21] (Scheme 1, adduct **A**).

In the reactions of N-arylmaleimides with thiourea and its derivatives, five- and six-membered heterocycles are usually formed: thiazoles **B** [1,7,13,14,15,16,17,18] and thiazines **C** [20]. Reactions of N-arylmaleimides with polynucleophilic reagents, such as amidinothiourea and thiosemicarbazones proceed at 1,3-S,N-binucleophilic centers with the formation of the corresponding thiazolines [2,3,21,22]. The reaction of N-arylmaleimides with heterocyclic 1,3-N,S-binucleophiles (for example, 2-thioquinazolines [7] and 2-mercapto-1,2,4-triazoles [5]) leads to the formation of condensed systems, such as thiazolo[2,3-b]quinazolines and thiazolo[3,2-b][1,2,4]triazoles, respectively. Thioethers—products of sulfur atom addition to the double bond of N-arylmaleimides by the Michael reaction type were isolated for 2-mercapto-1,2,4-triazole [6].

The reactions of the simplest 1,3-N,S-binucleophile thioacetamide with N-arylmaleimides are described in only one publication [23]. The authors reported an unusual course of the reaction of thioacetamide with N-phenylmaleimide when the initial reagents were heated under nitrogen in dioxane. They showed that two molecules of N-phenylmaleimide are involved in the reaction with thioacetamide. It was reported that this interaction proceeds as a cascade process. In the first stage, a thiazole **E_1_** is formed, which then acts as a 1,3-dipolarophile and reacts with another N-arylmaleimide molecule in the second stage to form 2-(4-methyl-1,3,6-trioxo-2-phenyloctahydro-7*H*-4,7-epithiopyrrolo[3,4-c]pyridin-7-yl)-N-phenylacetamide **1a** [23].

The purpose of this work was to study, in greater depth, the interaction of thioacetamide with N-substituted maleimides under various conditions to obtain new hetero-cyclic compounds with potential biological activity.

As a result of this study, we found that, under different conditions the reaction of thioacetamide with N-arylmaleimides can lead to 2-(4-methyl-1,3,6-trioxo-2-aryloctahydro-7*H*-4,7-epithiopyrrolo[3,4-c]pyridin-7-yl)-N-arylacetamides **1**, 2-(2-aryl-4-methyl-1,3,6-trioxo-2,3,5,6-tetrahydro-1*H*-pyrrolo[3,4-c]pyridin-7-yl)-N-arylacetamides **2** or 3,3′-thiobis(1-arylpyrrolidine-2,5-diones) **3**. We found that 2,3,5,6-tetrahydro-1*H*-pyrrolo[3,4-c]pyridines **2** can be obtained by heating 4,7-epithiopyrrolo[3,4-c]pyridines **1** in dioxane, in acetic acid or in methanol in the presence of sodium methoxide. The reaction of thioacetamide with N-alkylmaleimides under all the above conditions led predominantly to the formation of the corresponding sulfides **3**. Compounds **2** and **3** were obtained for the first time.

## 2. Results and Discussion

**Scheme 2:**
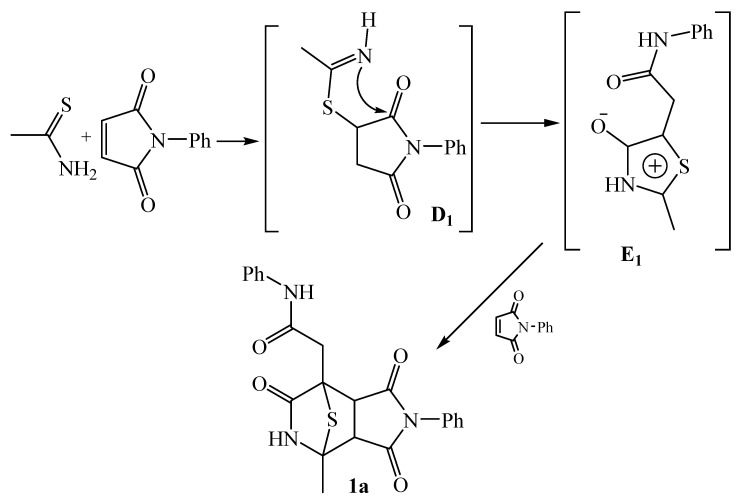
Interaction of thioacetamide and N-phenylmaleimide during heating in dioxane [23].

It was previously shown that refluxing thioacetamide and N-phenylmaleimide in dioxane in an inert atmosphere results in the formation of tricyclic compound **1a**—the yield of which depends on the ratio of the reagents (Scheme 2) [23].

2-(4-Methyl-1,3,6-trioxo-2-phenyloctahydro-7*H*-4,7-epithiopyrrolo[3,4-c]pyridin-7-yl)-N-phenylacetamide **1a** was the only reaction product described by the authors. At the first stage, the sulfur atom of thioacetamide is added to the double bond of the N-phenylmaleimide to form adduct **D_1_** [23]. The addition of S-nucleophiles by the Michael reaction to the double bond of N-substituted maleimides is widely used in organic synthesis [24,25,26,27]. Similar intermediates are also formed in reactions with other binucleophilic substrates [6,19,21].

The second stage of the process consists in the recyclization reaction of the succinimide ring due to the nucleophilic attack of the imino group at the nearest carbonyl group with the formation of thiazole **E_1_**. It is important to note that it was not possible to isolate this intermediate, because even at an equimolar ratio of the reactants, the next stage proceeds immediately. The third stage is a 1,3-dipolar cycloaddition reaction. Intermediate **E_1_** acts as a 1.3-dipole, and a N-phenylmaleimide molecule acts as a dipolarophile [23]. Cycloaddition reactions involving N-substituted maleimides are used in the synthesis of biologically active compounds [8,9,10,11].

In this work, the reactions of thioacetamide with N-phenylmaleimide were studied under various conditions (Table 1). Analysis of the reaction mass was conducted by thin layer chromatography and LC-MS. The reaction was performed at a molar ratio of thioacetamide: N-phenylmaleimide equal to 1:2.

**Scheme 3:**
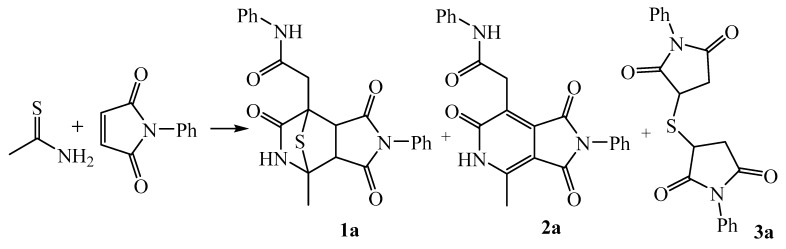
Reaction products of thioacetamide and N-phenylmaleimide.

We found that refluxing the starting compounds in dioxane led to the formation of a mixture of three basic substances 2-(4-methyl-1,3,6-trioxo-2-phenyloctahydro-7*H*-4,7-epithiopyrrolo[3,4-c]pyridine-7-yl)-N-phenylacetamide **1a**, 2-(2-phenyl-4-methyl-1,3,6-trioxo-2,3,5,6-tetrahydro-1*H*-pyrrolo[3,4-c]pyridin-7-yl)-N-phenylacetamide **2a** and 3,3′-thiobis(1-phenylpyrrolidine-2,5-dione) **3a**. The ratio of these components depended on the reaction time (Scheme 3).

More extensive monitoring of reaction conditions demonstrated that solvents and temperature influence the direction of the reaction. When the initial reagents were stirred at room temperature in dioxane, an extremely low conversion of the initial reagents was observed. Stirring a mixture of thioacetamide and N-phenylmaleimide in dioxane at 50 °C made it possible to obtain the tricyclic bridging compound **1a** at the maximum yield. Stirring the reagents at room temperature in THF, ethyl acetate and acetonitrile also led to the formation of compound **1a** as the major component of the mixture; however, the content of impurities under these conditions was higher.

The portionwise addition of N-phenylmaleimide to a refluxing solution of thioacetamide in dioxane led to the formation of predominantly compound **2a**, which is the product of the elimination of a hydrogen sulfide molecule from compound **1a**. The use of acetic acid as a solvent, as well as a mixture of dioxane and acetic acid, led to the formation of a mixture of compounds **2** and **3** in various proportions. When using boiling chloroform and methanol as solvents, a low conversion of the starting materials was observed.

**Scheme 4:**
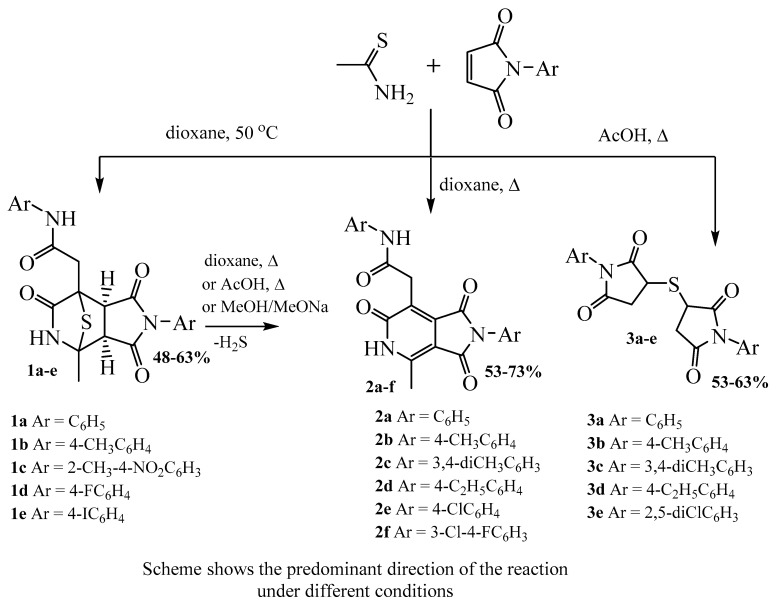
Reaction products of thioacetamide and N-arylmaleimides.

The extension of the reaction to other N-arylmaleimides led to similar results—the formation of compounds **1**, **2** and **3** in various ratios. Scheme 4 shows the predominant direction of the reaction under different conditions.

Based on the monitoring of the reaction conditions, we chose methods for the synthesis of compounds **1**–**3** that provided a good yield of the target products. Compounds **1a**–**e** were obtained by stirring the starting reagents at 50 °C in a dioxane medium for 12–15 h. Pyrrolo[3,4-c]pyridine-1,3,6-triones **2a**–**f** were obtained as the major component of the mixture by the portionwise addition of N-arylmaleimide to a refluxing solution of thioacetamide in dioxane and isolated in pure form. 3,3′-Thiobis(1-arylpyrrolidine-2,5-diones) **3a**–**e** was successfully isolated as a major product through the portionwise addition of N-arylmaleimide to a solution of thioacetamide in refluxing acetic acid.

Under milder conditions at 50 °C, the reaction likely proceeds under kinetic control with the formation of epithiopyrrolo[3,4-c]pyridines **1**. At higher temperatures, hydrogen sulfide was eliminated from compounds **1**, accompanied by the formation of conjugated system **2** (Scheme 4).

**Scheme 5:**
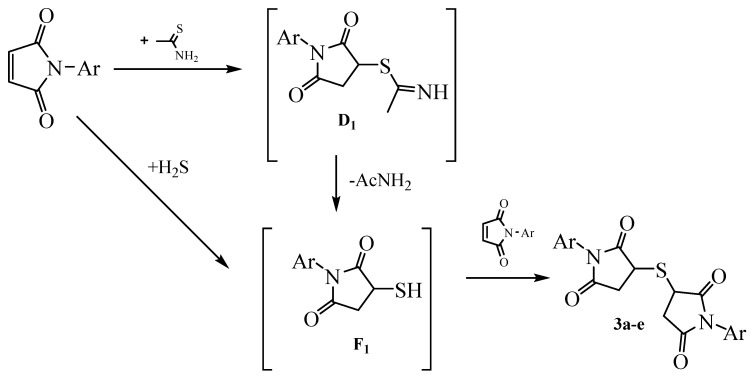
Probable pathways for the formation of 3,3′-thiobis(1-arylpyrrolidine-2,5-diones) **3**.

The emitted hydrogen sulfide can react with the starting N-arylmaleimide to form 3-mercapto-1-arylpyrrolidine-2,5-dione **F_1_** as an intermediate that then reacts with another N-arylmaleimide molecule and forms sulfides **3** [28,29,30] (Scheme 5). It is possible that the fractional addition of N-arylmaleimide to thioacetamide boiling in dioxane prevents the formation of compounds **3** because the emitted hydrogen sulfide leaves the reaction sphere and no intermediates **F_1_** are formed. As a result, product **2** accumulates in the reaction medium.

Heating of the initial reagents in acetic acid likely leads to an alternative pathway for the formation of 3,3′-thiobis(1-arylpyrrolidine-2,5-diones) **3a**–**e**. This pathway is based on the hydrolysis of S-alkylthioacetamides **D_1_** in an acidic medium under the action of water contained in acetic acid. These conditions also led to 3-mercapto-1-arylpyrrolidine-2,5-diones **F_1_**, which adds to the double bond of another N-arylmaleimide molecule to form sulfides **3a**–**e**. The fractional addition of N-arylmaleimide increases the selectivity of the reaction.

As noted earlier, the formation of products **1** includes a cycloaddition step. This process proceeds stereoselectively with the formation of exoisomers. This conclusion was made based on the interpretation of ^1^H NMR spectroscopy data from the position of the characteristic signals of CH-protons. In the spectra of compounds **1**, two CH-proton doublets are seen at 3.59 and 3.72 ppm, and the spin–spin coupling constant is 6.6 Hz, which is typical for exoisomers. For endoisomers, the signals of these protons are shifted to a weaker field [12,23], and the spin–spin coupling constant is increased [23].

The proposed pathway for the formation of pyrrolo[3,4-c]pyridine-1,3,6-triones **2** includes all stages of the formation of tricyclic compound **1** followed by elimination of the hydrogen sulfide molecule. This assumption is confirmed by the fact that compounds **2** were formed by refluxing epithiopyridine **1** in dioxane, in acetic acid or in methanol in the presence of sodium methoxide. This is consistent with the literature data. Previously, reactions of this kind were studied for tetrahydro-5*H*-4,8-epithio[1,2,5]oxadiazolo[3,4-f]isoindole-5,7(6*H*)-diones [12]. Hydrogen sulfide was eliminated in methanol in the presence of sodium methoxide, which led to the formation of 5*H*-[1,2,5]oxadiazolo[3,4-f]isoindole-5,7(6H)-dione. The starting tricyclic compounds, in turn, were obtained as a result of the cycloaddition reaction of N-substituted maleimides to thieno[3,4-c]-1,2,5-oxadiazole [12].

**Scheme 6:**
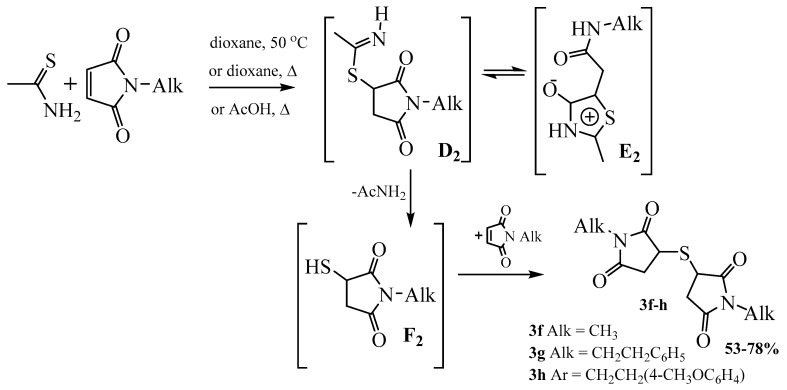
Reaction of thioacetamide and N-alkylmaleimides.

To expand the range of compounds **1**–**3**, the reactions of thioacetamide and N-akylmaleimides were studied. We found that, under all the previously described conditions (keeping in dioxane at 50 °C and at the boiling point or boiling in acetic acid), 3,3′-thiobis(1-alkylpyrrolidine-2,5-diones) **3g**–**h** were predominantly formed, and products **1** and **2** were formed only in small amounts (less than 5%). In this regard, only the major products, dialkyl sulfides **3f**–**h**, were isolated in high yields (Scheme 6). This is likely due to two facts: (1) the reversibility of the **E_2_** dipole formation reaction and (2) the lower activity of N-alkylmaleimides in 1,3-dipolar cycloaddition reactions, which is due to the electron-donating effect of their alkyl groups [31,32]. In this regard, the stage of hydrolysis of S-alkylthioacetamide **D_2_** with the formation of mercaptan **F_2_** becomes predominant. The reaction of the latter with another molecule of N-alkylmaleimide led to dialkyl sulfides **3f**–**h**.

Compounds **1**–**3** are of interest as substances with potential biological activity. Pyrrolo[3,4-c]pyridine derivatives are known to exhibit a wide spectrum of biological activity [33]. For example, the pyrrolo[3,4-c]pyridine-1,3,6-trione fragment can be used to create compounds insensitive to HIV-1 integrase mutations, which have potential in the treatment of AIDS [34,35]. Sulfides, structurally similar to compounds **3**, are able to inhibit matrix metalloprotease and can be used as agents against osteoarthritis and rheumatoid arthritis as well as an agent for inhibiting the metastasis of various types of cancer [36].

The structure of the compounds obtained by us was confirmed by ^1^H NMR and ^13^C NMR spectroscopy. Thus, in the ^1^H NMR spectra of compounds **1a**–**e,** singlets in the regions of 9.15–9.18 ppm and 9.70–10.18 ppm correspond to the protons of two NH-groups. Along with the signals of aromatic protons and substituent protons in the aromatic nucleus in the corresponding regions, the spectra contain a singlet of the methyl group (1.82–1.84 ppm) and two doublets of the protons of the methylene group at 3.18–3.20 ppm and 3.27–3.29 ppm as well as two characteristic doublets of CH-protons (3.59 and 3.72 ppm).

In the ^1^H NMR spectra of compounds **2a**–**f**, doublets of two protons of CH-groups disappear, the singlet of the methyl group shifts towards a weaker field (2.56–2.58 ppm), and the signals of the protons of the methylene group appear as a singlet in the region of 3.98–4.01 ppm. The proton signals of aryl substituents essentially did not change their position. The structure of compound **3b** was confirmed by X-ray diffraction analysis. X-ray diffraction data were collected at 100 K on a Bruker Quest D8 diffractometer equipped with a Photon-III area-detector (shutterless φ- and ω-scan technique), using graphite-monochromatized Mo Kα-radiation.

The intensity data were integrated by the SAINT program [37] and corrected for absorption and decay using SADABS [38]. The structure was solved by direct methods using SHELXT [39] and refined on F [38] using SHELXL-2018 [40]. All non-hydrogen atoms were refined with anisotropic displacement parameters. Hydrogen atoms were placed in ideal calculated positions and refined as riding atoms with relative isotropic displacement parameters. The SHELXTL program suite [37] was used for molecular graphics.

**Figure 1:**
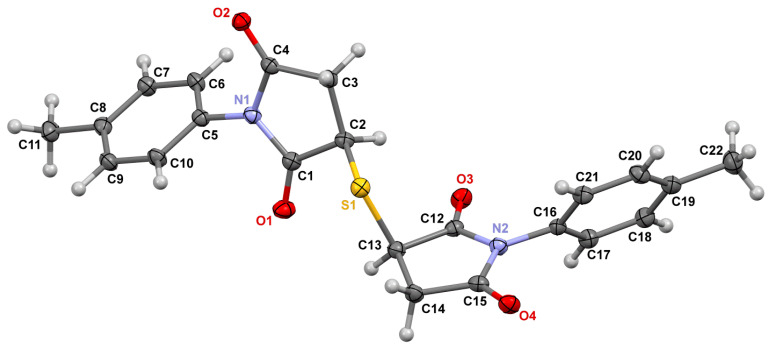
Molecular structure of 3,3′-thiobis (1-p-tolylpyrrolidine-2,5-dione) **3b** (*p* = 50%).

3,3′-Thiobis(1-p-tolylpyrrolidine-2,5-dione) **3b** crystalized from DMSO as crystallosolvate **3b**•DMSO. The molecular structure of **3b** is shown in Figure 1. Two moieties C_11_H_10_NO_2_ are connected via a sulfur atom with the C2-S1-C13 angle of 102.28(9)°. In each C_11_H_10_NO_2_ moiety, the N atom is in a trigonal planar environment. Due to N-C π-conjugation, the C(=O) N C(=O) fragment is planar, and the N-C bond distances lie in the range of 1.388(3) Å to 1.401(2) Å, which corresponds to a bond order of ~1.5. The N Cipso(Ph) bond distances (1.434(2) Å and 1.432(2) Å for N1-C5 and N2-C16) are only slightly shorter than single bonds.

The dihedral angle between planes defined by atoms of the aromatic ring (C6) and atoms of the C(=O) N C(=O) fragment is 64.3(1)° for atoms C5, …, C10 and O1, C1, N1, C4 and O2 and 59.9(1)° for C16, …, C21 and O3, C12, N2, C15 and O4. Therefore, the π conjugation within the C(=O) N C(=O) fragment is evidently present; however, the conjugation between this fragment and the π-system of the p-tolyl group is virtually absent.

**Figure 2:**
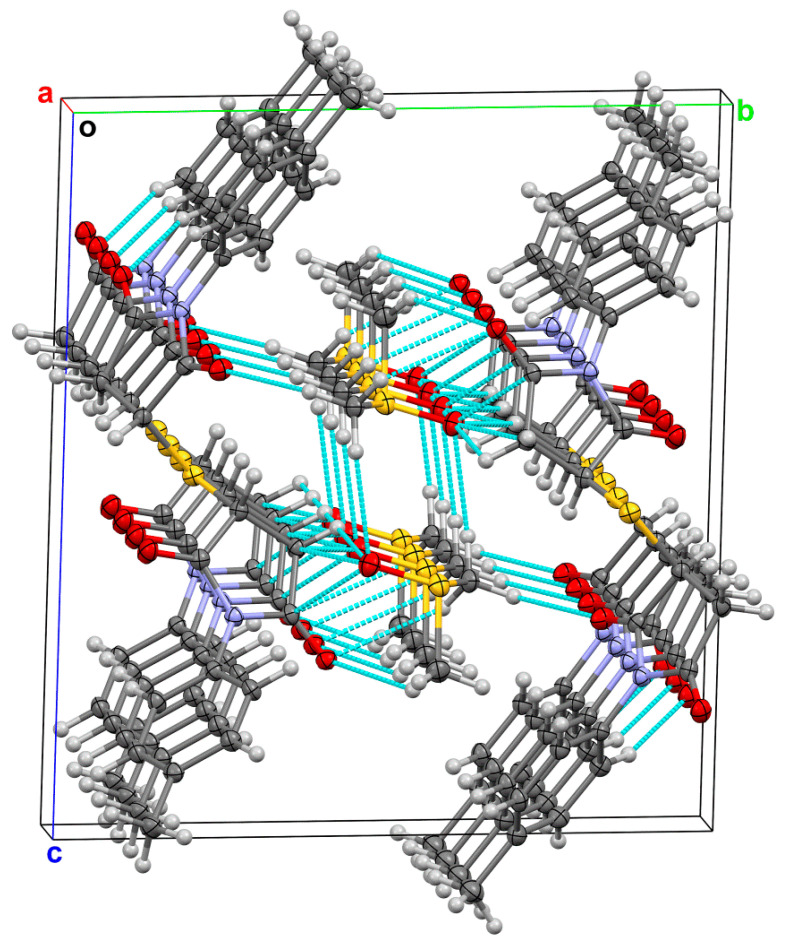
Packing plot of **3b**•DMSO along the a axis (*p* = 50%). Non-covalent short contacts are shown with blue dashed lines.

In the crystalline lattice, DMSO molecules are located in crystal channels (Figure 2) between layers of molecules **3b**. Non-covalent intermolecular contacts are formed between two neighboring molecules of DMSO or neighboring molecules of DMSO and **3b**. Intramolecular O…H-C interactions were also observed in molecules **3b** (Figure 2).

CCDC 2216201 contains the supplementary crystallographic data for this paper. These data can be obtained free of charge via http://www.ccdc.cam.ac.uk/conts/retrieving.html (accessed on 28 October 2022) (or from the CCDC, 12 Union Road, Cambridge CB2 1EZ, UK; Fax: +44 1223 336033; E-mail: deposit@ccdc.cam.ac.uk). Crystal data for **3b**•DMSO (C_22_H_20_N_2_O_4_S•C_2_H_6_OS), FW = 486.59, triclinic crystal system, P, a = 5.6417(2)Å, b = 13.7700(5)Å, c = 15.1459(6)Å, α = 92.238(1)°, β = 95.040(1)°, γ = 95.809(1)°, V = 1164.69(8)Å3, Z = 2, T = 100(2) K, μ(MoKα) = 0.268 mm^−1^, Dcalc = 1.387 g·cm^−3^, θmax = 34.98°, reflections collected 60803, independent 10230 (Rint = 0.1044), observed (with I > 2σ(I)) 6257, final R1 = 0.0683 (I > 2σ(I)), wR2 = 0.1463 (all data). A detailed description of the X-ray structural analysis data is presented in the Supplementary Material.

In the ^1^H NMR spectra of compounds **3a**–**e**, along with the signals of aryl substituents in the corresponding regions, there are doublets of doublets of protons of two CH groups in the regions of 4.37–4.52 and 4.62–4.75 ppm as well as signals of four protons of CH_2_ groups in the form of two doublets of doublets (2.63–2.83 ppm and 2.88–2.92 ppm) and a multiplet in the region of 3.28–3.41 ppm. It should be noted that compounds **3** have two chiral centers, while the nature of the ^1^H NMR spectra of 3,3′-thiobis(1-arylpyrrolidine-2,5-diones) **3a**–**e** indicates the formation of only one pair of enantiomers. The absolute configuration of chiral carbon atoms was determined on the basis of the X-ray diffraction data. We found that, in the case of N-arylmaleimides, the formation of thioethers **3a**–**e** proceeded diastereoselectively with the formation of a mixture of R,R- and S,S-isomers. The formation of the meso form was not observed.

Analysis of the ^1^H NMR spectra of compounds **3f**–**h** showed that the introduction of an aliphatic substituent instead of an aromatic substituent slightly changed the position of the signals. The signals of all protons of the pyrrolidine cycles for N-alkyl derivatives shifted upfield. The spectrum of compound **3f** showed three sets of signals corresponding to the CH and CH_2_ protons of the pyrrolidine rings as a multiplet at 2.49–2.51 ppm, two doublets at 2.54 and 2.68 ppm and a doublet of doublets at 3.15 ppm. The singlets of the two methyl groups were located at 2.82 and 2.83 ppm.

In the spectra of compounds **3g**–**h**, the doubling of all signals was observed, which allows us to conclude that, in addition to the pair of R,R- and S,S-enantiomers, the meso form was formed at a ratio of 2:1. The signals of the CH protons of the pyrrolidine rings of the meso form shifted upfield in compare to the signals of the protons of the R,R- and S,S-enantiomers. In addition to the signals corresponding to the protons of the CH and CH_2_ groups of the pyrrolidine rings, the spectra contain signals of two pairs of methylene groups in the region of 3.04–3.14 ppm and 3.52–3.60 ppm as well as aromatic proton signals in the corresponding regions. In the spectrum of compounds **3h**, singlets of methoxy groups were visible at 3.67 and 3.68 ppm. Thus, compounds **3g**–**h** were isolated as a mixture of stereoisomers and could not be separated.

It should be noted that the literature describes methods for the preparation of some sulfides containing a pyrrolidine-2,5-dione fragment in their structure and similar in structure to compounds **3**. These are obtained as a result of the interaction of N-substituted maleimides with hydrogen sulfide or alkyl mercaptans [28,29,30]. These reactions are used for the quantitative determination of hydrogen sulfide or mercaptans [28,29]. An alternative method for the synthesis of such systems is the interaction of the corresponding amines with thiodisuccinic acid [36]. Thus, in this work, a number of new 3,3′-thiobis(1-arylpyrrolidine-2,5-diones) and 3,3′-thiobis(1-alkylpyrrolidine-2,5-diones) were obtained, and a new method for their synthesis was proposed.

## 3. Materials and Methods

### 3.1. General

^1^H NMR, ^13^C NMR and NOESY spectra were recorded on BrukerDRX-500 devices (500.13 and 125.75 MHz) in DMSO-*d*6 or CDCl_3_ and TFA-d with an internal TMS standard. Melting points were taken on a Stuart SMP30 device (Cole-Parmer Ltd., St. Neots, UK). HPLC/MS spectra were recorded on an Agilent Infinity 1260 chromatograph (Agilent Technologies, Palo Alto, CA, USA) with MS interface Agilent 6230 TOFLC/MS. Conditions for the separation: mobile phase MeCN/H_2_O + 0.1% FA (formic acid), gradient elution (first CH_3_CN:H_2_O (60:40) and then for 5 min to 90% CH_3_CN), column—Poroshell 120 EC-C18 (4.6 × 50 mm, 2.7 μm), thermostat 23–28 °C and flow rate of 0.3–0.4 mL/min electrospray ionization (capillary—3.5 kV; fragmentor +191 V; and OctRF +66 V—positive polarity). The course of the reaction and the purity of the obtained compounds were controlled by TLC on Merck TLC Silica gel 60 F254 plates in a 20:1 CHCl_3_–MeOH system (visualization under UV light). The commercially available reagents were purchased from Acros Organics (Geel, Belgium).

### 3.2. X-ray Analysis

X-ray diffraction data were collected at 100 K on a Bruker Quest D8 diffractometer equipped with a Photon-III area-detector (shutterless φ- and ω-scan technique) using graphite-monochromatized Mo Kα-radiation. The intensity data were integrated by the SAINT program [37] and corrected for absorption and decay using SADABS [38]. The structure was solved by direct methods using SHELXT [39] and refined on F [38] using SHELXL-2018 [40]. All non-hydrogen atoms were refined with anisotropic displacement parameters. Hydrogen atoms were placed in ideal calculated positions and refined as riding atoms with relative isotropic displacement parameters. The SHELXTL program suite [37] was used for the molecular graphics. CCDC 2216201 contains the supplementary crystallographic data for this paper. These data can be obtained free of charge via http://www.ccdc.cam.ac.uk/conts/retrieving.html (accessed on 28 October 2022) (or from the CCDC, 12 Union Road, Cambridge CB2 1EZ, UK; Fax: +44 1223 336033; E-mail: deposit@ccdc.cam.ac.uk). The crystal data and structure refinement for **3b** are presented in the Table 2.

### 3.3. General Procedure for the Preparation of 2-(2-Aryl-4-methyl-1,3,6-trioxooctahydro-7H-4,7-epithiopyrrolo[3,4-c]pyridin-7-yl)-N-arylacetamides (***1a***–***e***)

A mixture of thioacetamide (1.5 mmol) and N-arylmaleimide (3 mmol) was stirred in 10 mL of 1,4-dioxane for 12–15 h at 50 °C. The precipitate that formed was washed with 1,4-dioxane and filtered to furnish the desired products **1a**–**e**.

#### 3.3.1. 2-(4-Methyl-1,3,6-trioxo-2-phenyloctahydro-1H-4,7-epithiopyrrolo[3,4-c]pyridin-7-yl)-N-phenylacetamide (**1a**)

White powder (yield 0.40 g, 63%), m.p. > 300 °C; ^1^H NMR (500 MHz, DMSO-*d*6, δ ppm): 1.83 (s, 3H, CH_3_), 3.24 (d, J = 16.2 Hz, 1H, CH_2_), 3.27 (d, J = 16.2 Hz, 1H, CH_2_), 3.59 (d, J = 6.6 Hz, 1H, CH), 3.72 (d, J = 6.6 Hz, 1H, CH), 7.00–7.57 (m, 10H, Ar), 9.15 (s, 1H, NH), 10.07 (s, 1H, NH); ^13^C NMR (125 MHz, DMSO-*d*6, δ ppm) [23]: δ 17.0, 36.9, 50.2, 58.4, 63.2, 72.4, 118.9, 122.9, 126.6, 128.4, 128.9, 131.9, 139.0, 167.0, 173.0, 173.4, 175.7; HPLC-HRMS (ESI) calcd for C_22_H_19_N_3_O_4_S [M + H]^+^: 422.1170; found: 422.1172.

#### 3.3.2. 2-(4-Methyl-1,3,6-trioxo-2-(p-tolyl)octahydro-1H-4,7-epithiopyrrolo[3,4-c]pyridin-7-yl)-N-(p-tolyl)acetamide (**1b**)

White powder (yield 0.37 g, 55%), m.p. > 300 °C; ^1^H NMR (500 MHz, DMSO-*d*6, δ ppm): 1.84 (s, 3H, CH_3_), 2.34 (s, 3H, CH_3_), 2.51 (s, 3H, CH_3_), 3.18–3.74 (m, 4H, CH_2_ + 2CH), 7.03–7.51 (m, 8H, Ar), 9.15 (br s, 1H, NH), 10.01 (br s, 1H, NH); ^13^C NMR (125 MHz, DMSO-*d*6, δ ppm): 17.6, 20.9, 21.2, 33.9, 50.8, 59.0, 63.9, 73.1, 119.6, 126.8, 127.1, 129.4, 129.8, 130.0, 132.5, 135.1, 138.8, 167.4, 173.8, 174.2, 176.5; HRMS (ESI) calcd for C_24_H_23_N_3_O_4_S [M + H]^+^: 450.1483; found: 450.1485.

#### 3.3.3. 2-(4-Methyl-2-(2-methyl-4-nitrophenyl)-1,3,6-trioxooctahydro-1H-4,7-epithiopyrrolo[3,4-c]pyridin-7-yl)-N-(2-methyl-4-nitrophenyl)acetamide (**1c**)

White powder (yield 0.38 g, 48%), m.p. > 300 °C, ^1^H NMR (500 MHz, DMSO-*d*6, δ ppm): 1.86 (s, 3H, CH_3_), 2.48 (s, 3H, CH_3_), 2.51 (s, 3H, CH_3_), 3.20–3.98 (m, 4H, CH_2_ + 2CH), 7.83–8.32 (m, 6H, Ar), 9.18 (s, 1H, NH), 9.70 (s, 1H, NH); ^13^C NMR (125 MHz, DMSO-*d*6, δ ppm): 17.3, 33.8, 32.4, 39.5, 50.9, 59.6, 63.3, 72.9, 122.1, 123.3, 125.8, 130.3, 131.4, 137.4, 139.0, 142.9, 143.5, 148.1, 165.1, 168.4, 173.1, 176.3; HRMS (ESI) calcd for C_24_H_21_N_5_O_8_S [M + H]^+^: 540.1185; found: 540.1186.

#### 3.3.4. N-(4-Fluorophenyl)-2-(2-(4-fluorophenyl)-4-methyl-1,3,6-trioxooctahydro-1H-4,7-epithiopyrrolo[3,4-c]pyridin-7-yl)acetamide (**1d**)

White powder (yield 0.35 g, 51%), m.p. > 300 °C; ^1^H NMR (500 MHz, DMSO-*d*6, δ ppm): 1.83 (s, 3H, CH_3_), 3.12–3.29 (m, 2H, CH_2_), 3.60 (d, J = 6.6 Hz, 1H, CH), 3.72 (d, J = 6.6 Hz, 1H, CH), 7.09–7.65 (m, 8H, Ar), 9.18 (br s, 1H, NH), 10.18 (br s, 1H, NH); 13C NMR (125 MHz, DMSO-*d*6, δ ppm): 17.6, 34.0, 50.8, 59.1, 63.7, 73.1, 122.2, 122.4, 126.0, 126.1, 128.7, 128.8, 132.3, 137.5, 167.5, 173.4, 173.9, 176.3; HRMS (ESI) calcd for C_22_H_17_F_2_N_3_O_4_S [M + H]^+^: 458.0981; found: 458.0986.

#### 3.3.5. N-(4-Iodophenyl)-2-(2-(4-iodophenyl)-4-methyl-1,3,6-trioxooctahydro-1H-4,7-epithiopyrrolo[3,4-c]pyridin-7-yl)acetamide (**1e**)

White powder (yield 0.59 g, 59%), m.p. > 300 °C; ^1^H NMR (500 MHz, DMSO-*d*6, δ ppm): 1.82 (s, 3H, CH_3_), 3.17 (d, J = 16.4, 1H, CH_2_), 3.24 (d, J = 16.4, 1H, CH_2_), 3.54–3.58 (m, 1H, CH), 3.70 (d, J = 6.6 Hz, 1H, CH), 7.03 (d, J = 8.6 Hz, 2H, Ar), 7.38 (d, J = 8.6 Hz, 2H, Ar), 7.61 (d, J = 8.5 Hz, 2H, Ar), 7.86 (d, J = 8.5 Hz, 2H, Ar), 9.18 (s, 1H, NH), 10.08 (s, 1H, NH); ^13^C NMR (125 MHz, DMSO-*d*6, δ ppm): 17.6, 34.0, 50.9, 63.8, 73.0, 87.0, 95.4, 121.8, 129.4, 132.2, 137.6, 138.5, 139.4, 167.9, 173.4, 173.8, 176.3; HRMS (ESI) calcd for C_22_H_17_I_2_N_3_O_4_S [M + H]^+^: 673.9102; found: 673.9100.

### 3.4. General Procedure for the Preparation of 2-(4-Methyl-1,3,6-trioxo-2-aryl-2,3,5,6-tetrahydro-1H-pyrrolo[3,4-c]pyridin-7-yl)-N-arylacetamides (***2a***–***f***)

Method A: N-Arylmaleimide (3 mmol) was added in portions to a boiling solution of thioacetamide (1.5 mmol) in dioxane (10 mL). The mixture was refluxed for 2 h. The precipitated was filtered and recrystallized from i-PrOH.

Method B: 2-(4-Methyl-1,3,6-trioxo-2-aryloctahydro-1*H*-4,7-epithiopyrrolo[3,4-c]pyridin-7-yl)-N-arylacetamide 1 (1 mmol) was refluxed in 5 mL of dioxane for 10–12 h until the reaction was completed according to the TLC data. The precipitated was filtered and recrystallized from i-PrOH.

Method C: 2-(4-Methyl-1,3,6-trioxo-2-aryloctahydro-1*H*-4,7-epithiopyrrolo[3,4-c]pyridin-7-yl)-N-arylacetamide 1 (1 mmol) was refluxed in 5 mL of acetic acid for 7 h until the reaction was completed according to the TLC data. The precipitated was filtered and recrystallized from i-PrOH.

Method D: A mixture of 2-(4-methyl-1,3,6-trioxo-2-aryloctahydro-1*H*-4,7-epithiopyrrolo[3,4-c]pyridin-7-yl)-N-arylacetamide 1 (1 mmol) and sodium methoxide (cat.) was stirred in 5 mL MeOH at r.t. until the reaction was completed according to the TLC data. The precipitated was filtered and recrystallized from i-PrOH.

#### 3.4.1. 2-(4-Methyl-1,3,6-trioxo-2-phenyl-2,3,5,6-tetrahydro-1H-pyrrolo[3,4-c]pyridin-7-yl)-N-phenylacetamide (**2a**)

White powder (yield: Method A: 0.38 g, 65%; Method B: 0.20 g, 53%; Method C: 0.26 g, 68%; Method D: 0.28 g, 73%) m.p. > 300 °C. ^1^H NMR (500 MHz, DMSO-*d*6, δ ppm): 2.59 (s, 3H, CH_3_), 4.01 (s, 2H, CH_2_), 6.99–7.58 (m, 10H, Ar), 10.12 (s, 1H, NH), 12.55 (s, 1H, NH); ^13^C NMR (125 MHz, DMSO-*d*6, δ ppm): 15.2, 32.6, 103.6, 118.8, 120.7, 124.9, 126.7, 128.9, 131.1, 132.0, 138.7, 148.3, 164.5, 165.1, 165.9, 167.9; HRMS: m/z calcd for C_22_H_17_N_3_O_4_ [M + H]^+^: 388.1293; found: 388.1295.

#### 3.4.2. 2-(4-Methyl-1,3,6-trioxo-2-(p-tolyl)-2,3,5,6-tetrahydro-1H-pyrrolo[3,4-c]pyridin-7-yl)-N-(p-tolyl)acetamide (**2b**)

White powder (yield Method A: 0.34 g, 55%), m.p. > 300 °C. ^1^H NMR (500 MHz, DMSO-*d*6, δ ppm): 2.20 (s, 3H, CH_3_), 2.53 (s, 3H, CH_3_), 2.58 (s, 3H, CH_3_), 4.00 (s, 2H, CH_2_), 6.99–7.55 (m, 8H, Ar), 10.03 (br s, 1H, NH), 12.50 (br s, 1H, NH); ^13^C NMR (125 MHz, DMSO-*d*6, δ ppm): 15.2, 20.3, 20.6, 32.0, 103.6, 118.8, 123.7, 126.7, 127.0, 129.0, 129.3, 131.8, 136.7, 136.8, 137.7, 147.7, 163.9, 164.8, 165.6, 166.8; HRMS: m/z calcd. for C_24_H_21_N_3_O_4_ [M + H]^+^: 416.1606; found: 416.1604.

#### 3.4.3. N-(3,4-Dimethylphenyl)-2-(2-(3,4-dimethylphenyl)-4-methyl-1,3,6-trioxo-2,3,5,6-tetrahydro-1H-pyrrolo[3,4-c]pyridin-7-yl)acetamide (**2c**)

White powder (yield Method C: 0.30 g, 67%), m.p. > 300 °C. ^1^H NMR (500 MHz, DMSO-*d*6, δ ppm): 2.12 (s, 3H, CH_3_), 2.13 (s, 3H, CH_3_), 2.22 (s, 3H, CH_3_), 2.23 (s, 3H, CH_3_), 2.56 (s, 3H, CH_3_), 3.98 (s, 2H, CH_2_), 6.96–7.38 (m, 6H, Ar), 9.92 (s, 1H, NH), 12.45 (br s, 1H, NH). ^13^C NMR (125 MHz, DMSO-*d*6, δ ppm): 15.9, 19.3, 19.6, 19.8, 20.1, 32.6, 104.2, 117.0, 120.7, 124.2, 125.2, 128.6, 129.8, 130.1, 131.2, 136.8, 137.2, 137.4, 137.6, 148.3, 164.6, 165.5, 166.3, 167.4; HRMS: m/z calcd. for C_26_H_25_N_3_O_4_ [M + H]^+^: 444.1919; found: 444.1920.

#### 3.4.4. N-(4-Ethylphenyl)-2-(2-(4-ethylphenyl)-4-methyl-1,3,6-trioxo-2,3,5,6-tetrahydro-1H-pyrrolo[3,4-c]pyridin-7-yl)acetamide (**2d**)

White powder (yield Method C: 0.26 g, 58%), m.p. > 300 °C. ^1^H NMR (500 MHz, DMSO-*d*6, δ ppm): 1.14 (t, J = 7.6 Hz, 3H, CH_3_), 1.21 (t, J = 7.6 Hz, 3H, CH_3_), 2.52–2.54 (m, 4H, 2CH_2_), 2.58 (s, 3H, CH_3_), 4.00 (s, 2H, CH_2_), 7.12 (d, J = 8.3 Hz, 2H, Ar), 7.27 (d, J = 8.3 Hz, 2H, Ar), 7.34 (d, J = 8.3 Hz, 2H, Ar), 7.45 (d, J = 8.3 Hz, 2H, Ar), 10.01 (s, 1H, NH), 12.55 (br s, 1H, NH); ^13^C NMR (125 MHz, DMSO-*d*6, δ ppm): 15.5, 15.6, 15.9, 27.7, 28.0, 32.6, 103.6, 117.2, 124.7, 125.0, 126.7, 126.8, 128.2, 129.7, 129.8, 137.4, 137.5, 147.7, 164.6, 165.6, 166.0, 167.5; HRMS: m/z calcd. for C_26_H_25_N_3_O_4_ [M + H]^+^: 444.1919; found: 444.1923.

#### 3.4.5. N-(4-Chlorophenyl)-2-(2-(4-chlorophenyl)-4-methyl-1,3,6-trioxo-2,3,5,6-tetrahydro-1H-pyrrolo[3,4-c]pyridin-7-yl)acetamide (**2e**)

White powder (yield Method A: 0.37 g, 55%), m.p. > 300 °C. ^1^H NMR (500 MHz, DMSO-*d*6, δ ppm): 2.58 (s, 3H, CH_3_), 4.00 (s, 2H, CH_2_), 7.30 (d, J = 8.5 Hz, 2H, Ar), 7.42 (d, J = 8.5 Hz, 2H, Ar), 7.59 (d, J = 8.5 Hz, 4H, Ar), 10.29 (s, 1H, NH), 12.55 (br s, 1H, NH). ^13^C NMR (125 MHz, DMSO-*d*6, δ ppm): 15.9, 32.7, 104.2, 121.0, 124.1, 127.1, 129.2, 129.4, 129.6, 131.1, 133.3, 137.4, 138.7, 148.6, 164.5, 165.1, 165.9, 167.9; HRMS: m/z calcd. for C_22_H_15_N_3_O_4_Cl_2_ [M + H]^+^: 456.0513; found: 456.0516.

#### 3.4.6. N-(3-Chloro-4-fluorophenyl)-2-(2-(3-chloro-4-fluorophenyl)-4-methyl-1,3,6-trioxo-2,3,5,6-tetrahydro-1H-pyrrolo[3,4-c]pyridin-7-yl)acetamide (**2f**)

White powder (yield Method A: 0.43 g, 58%), m.p. > 300 °C. ^1^H NMR (500 MHz, DMSO-*d*6, δ ppm): 2.57 (s, 3H, CH_3_), 3.99 (s, 2H, CH_2_), 7.30–7.87 (m, 6H, Ar), 10.35 (s, 1H, NH), 12.57 (s, 1H, NH). ^13^C NMR (125 MHz, DMSO-*d*6, δ ppm): 16.0, 32.7, 104.1, 117.4, 117.6, 117.8, 119.5, 119.6, 119.7, 120.0, 120.1, 120.8, 123.8, 128.9, 129.0, 129.3, 130.2, 136.9, 137.4, 148.9, 156.2, 164.6, 164.9, 165.9, 168.0; HRMS: m/z calcd. for C_22_H_13_Cl_2_F_2_N_3_O_4_ [M + H]^+^: 492.0325; found: 492.0327.

### 3.5. General Procedure for the Preparation of 3,3′-Thiobis(1-arylpyrrolidine-2,5-diones) (***3a***–***e***)

N-Arylmaleimide (3 mmol) was added in portions to a boiling solution of thioacetamide (1.5 mmol) in 5 mL acetic acid. The mixture was then refluxed until completion of the reaction as evidenced by TLC. The precipitated was filtered and recrystallized from dioxane.

#### 3.5.1. 3,3′-Thiobis(1-phenylpyrrolidine-2,5-dione) (**3a**)

White powder (yield 0.36 g, 63%), m.p. 199–201 °C. ^1^H NMR (500 MHz, CDCl_3_, δ ppm): 2.65 (dd, J = 4.4 Hz, J = 18.8 Hz, 1H, CH), 2.89 (dd, J = 4.4 Hz, J = 18.7 Hz, 1H, CH), 3.30–3.39 (m, 2H, 2CH), 4.39 (dd, J = 4.4 Hz, J = 9.2 Hz, 1H, CH), 4.75 (dd, J = 4.4 Hz, J = 9.5 Hz, 1H, CH), 7.25–7.51 (m, 10H, Ar). ^1^H NMR (500 MHz, DMSO-*d*6, δ ppm): 2.83 (dd, J = 4.4 Hz, J = 18.4 Hz, 1H, CH), 2.92 (dd, J = 4.4 Hz, J = 16.0 Hz, 1H, CH), 3.32–3.41 (m, 2H, CH), 4.49 (dd, J = 4.4 Hz, J = 9.2 Hz, 1H, CH), 4.62 (dd, J = 4.4 Hz, J = 9.2 Hz, 1H, CH), 7.28–7.54 (m, 10H, Ar); ^13^C NMR (125 MHz, DMSO-*d*6, δ ppm): 35.4, 37.3, 40.5, 40.6, 127.5, 127.6, 129.0, 129.1, 129.4, 129.5, 132.8, 132.9, 174.5, 174.7, 175.8, 176.5; HRMS (ESI) calcd for C_20_H_16_N_2_O_4_S [M + H]^+^: 381.0904; found: 381.0906.

#### 3.5.2. 3,3′-Thiobis(1-(p-tolyl)pyrrolidine-2,5-dione) (**3b**)

White powder (yield 0.35 g, 58%), m.p. 263–265 °C. ^1^H NMR (500 MHz, DMSO-*d*6, δ ppm): δ 2.35 (s, 6H, 2CH_3_), 2.75–2.95 (m, 2H, 2CH), 3.27–3.40 (m, 2H, 2CH), 4.45 (m, 1H, CH), 4.50–4.65 (m, 1H, CH), 7.10–7.20 (m, 4H, Ar), 7.25–7.35 (m, 4H, Ar); ^13^C NMR (125 MHz, DMSO-*d*6, δ ppm): 21.3, 35.4, 37.3, 40.5, 40.6, 127.3, 127.4, 129.9, 130.2, 130.3, 138.5, 138.6; HRMS (ESI) calcd for C_22_H_20_N_2_O_4_S [M + H]^+^: 409.1218; found: 409.1219.

#### 3.5.3. 3,3′-Thiobis(1-(3,4-dimethylphenyl)pyrrolidine-2,5-dione) (**3c**)

White powder (yield 0.39 g, 60%), m.p. 167–169 °C. ^1^H NMR (500 MHz, CDCl_3_, δ ppm): 2.27–2.29 (m, 12H, 4CH_3_), 2.63 (dd, J = 4.3 Hz, J = 18.8 Hz, 1H, CH), 2.88 (dd, J = 4.3 Hz, J = 18.6 Hz, 1H, CH), 3.31 (dd, J = 9.5 Hz, J = 18.8 Hz, 1H, CH), 3.34 (dd, J = 9.3 Hz, J = 18.6 Hz, 1H, CH), 4.37 (dd, J = 4.4 Hz, J = 9.1 Hz, 1H, CH), 4.75 (dd, J = 4.3 Hz, J = 9.5 Hz, 1H, CH), 6.95–7.25 (m, 6H, Ar). ^13^C NMR (125 MHz, DMSO-*d*6, δ ppm): 19.6, 19.8 19.9, 35.4, 37.3, 40.5, 40.6, 124.8, 124.9, 128.2, 128.3, 130.2, 130.3, 130.4, 130.5, 137.3, 137.4, 137.5, 137.6, 174.6, 174.8, 175.8, 176.5. HRMS (ESI) calcd for C_24_H_24_N_2_O_4_S [M + H]^+^: 437.1531; found: 437.1533.

#### 3.5.4. 3,3′-Thiobis(1-(4-ethylphenyl)pyrrolidine-2,5-dione) (**3d**)

White powder (yield 0.36 g, 55%), m.p. 216–217 °C. ^1^H NMR (500 MHz, DMSO-*d*6, δ ppm): 1.10 (t, J = 7.5 Hz, 3H, CH_3_), 1.21 (t, J = 7.5 Hz, 3H, CH_3_), 2.41–2.62 (m, 4H, 2CH_2_), 2.85 (dd, J = 4.4 Hz, J = 18.4 Hz, 1H, CH), 2.95 (dd, J = 4.4 Hz, J = 16.0 Hz, 1H, CH), 3.32–3.41 (m, 2H, 2CH), 4.50 (dd, J = 4.4 Hz, J = 9.2 Hz, 1H, CH), 4.60 (dd, J = 4.4 Hz, J = 9.2 Hz, 1H, CH), 7.28 (d, J = 8.4 Hz, 2H, Ar), 7.45(d, J = 8.4 Hz, 2H, Ar), 7.55 (d, J = 8.4 Hz, 2H, Ar), 7.70 (d, J = 8.4 Hz, 2H, Ar); ^13^C NMR (125 MHz, DMSO-*d*6, δ ppm): 15.4, 27.8, 34.8, 36.7, 39.9, 40.0, 126.7, 126.8, 128.1, 128.2, 129.7, 128.8, 144.1, 144.2, 174.0, 174.1, 175.2, 175.9; HRMS (ESI) calcd for C_24_H_24_N_2_O_4_S [M + H]^+^: 437.1531; found: 437.1535.

#### 3.5.5. 3,3′-Thiobis(1-(2,5-dichlorophenyl)pyrrolidine-2,5-dione) (**3e**)

White powder (yield 0.41 g, 53%), m.p. 245–247 °C. ^1^H NMR (500 MHz, DMSO-*d*6, δ ppm): 2.84–3.01 (m, 2H, 2CH), 3.27–3.35 (m, 2H, 2CH), 4.50 (dd, J = 4.4 Hz, J = 9.2 Hz, 1H, CH), 4.63 (dd, J = 4.4 Hz, J = 9.2 Hz, 1H, CH), 6.80–7.45 (m, 6H, Ar); ^13^C NMR (125 MHz, DMSO-*d*6, δ ppm): 36.3, 37.2, 40.4, 40.6, 130.0, 130.1, 130.2, 130.3, 130.5, 131.0, 131.1, 131.3, 131.9, 132.0, 172.9, 173.5, 173.8, 174.4; HRMS (ESI) calcd for C_20_H_12_C_l4_N_2_O_4_S [M + H]^+^: 516.9345; found: 516.9348.

### 3.6. General Procedure for the Preparation of 3,3′-Thiobis(1-alkylpyrrolidine-2,5-diones) (***3f***–***h***)

Method A: A mixture of thioacetamide (1.5 mmol) and N-alkylmaleimide (3 mmol) was stirred in 10 mL of 1,4-dioxane for 12–15 h at 50 °C. The precipitate that formed was washed with 1,4-dioxane and filtered.

Method B: N-Alkylmaleimide (3 mmol) was added in portions to a boiling solution of thioacetamide (1.5 mmol) in dioxane (10 mL). The mixture was refluxed for 2 h. The precipitated was filtered and recrystallized from acetone.

Method C: N-Alkylmaleimide (3 mmol) was added in portions to a boiling solution of thioacetamide (1.5 mmol) in 5 mL acetic acid. The mixture was then refluxed until completion of the reaction as evidenced by TLC. The precipitated was filtered and recrystallized from acetone.

#### 3.6.1. 3,3′-Thiobis(1-methyl)pyrrolidine-2,5-dione) (**3f**)

White powder (yield Method A: 0.27 g, 62%; Method B: 0.28 g, 64%; Method C: 0.33 g, 78%), m.p.210–213 °C. ^1^H NMR (600 MHz, DMSO-*d*6, δ ppm): 2.49–2.51 (m, 2H, 2CH), 2.54 (d, J = 18.4 Hz, 1H, CH), 2.68 (d, J = 18.4 Hz, 1H, CH), 2.82 (s, 3H, CH_3_), 2.83 (s, 3H, CH_3_), 3.15 (dd, J = 2.8 Hz, J = 18.4 Hz, 2H, CH); ^13^C NMR (125 MHz, DMSO-*d*6, δ ppm): 25.2, 25.3, 35.2, 35.3, 36.7, 36.8, 175.4, 175.5, 177.2; HRMS (ESI) calcd for C_10_H_12_N_2_O_4_S [M + H]^+^: 289.0312; found: 289.0311.

#### 3.6.2. 3,3′-Thiobis(1-phenethylpyrrolidine-2,5-dione) (**3g**)

White powder (yield Method A: 0.35 g, 53%; Method B: 0.47 g, 72%; Method C: 0.53 g, 81%), m.p.121–122 °C. ^1^H NMR (500 MHz, DMSO-*d*6): Major δ: 2.45 (dd, J = 4.4 Hz, J = 18.4 Hz, 1H, CH), 2.59 (dd, J = 4.4 Hz, J = 18.4 Hz, 1H, CH_2_), 2.76–2.78 (m, 4H, 2CH_2_Ar), 3.12 (q, J = 9.2 Hz, 2H, 2CH), 3.54–3.64 (m, 4H, CH_2_N), 4.27 (dd, J = 4.1 Hz, J = 9.1 Hz, 2H, 2CH), 7.14–7.28 (m, 10H, Ar); Minor δ: 2.49–2.51 (m, 2H, 2CH), 2.76–2.78 (m, 4H, 2CH_2_Ar), 3.06 (q, J = 9.2 Hz, 2H, 2CH), 3.54–3.64 (m, 4H, 2CH_2_N), 3.97 (dd, J = 4.4 Hz, J = 9.1 Hz, 2H, 2CH), 7.14–7.28 (m, 10H, Ar); ^13^C NMR (125 MHz, DMSO-*d*6, δ ppm): 33.0, 33.1, 35.2, 36.6, 40.0, 40.1, 123.4, 126.9, 128.9, 129.1, 138.3, 138.4, 175.1, 175.3, 176.0, 176.8; HRMS (ESI) calcd for C_24_H_24_N_2_O_4_S [M + H]^+^: 437.1531; found: 437.1530.

#### 3.6.3. 3,3′-Thiobis(1-(4-methoxyphenethyl)pyrrolidine-2,5-dione) (**3h**)

White powder (yield Method A: 0.49 g, 66%; Method B: 0.51 g, 69%; Method C: 0.52 g, 70%), m.p.137–139 °C. ^1^H NMR (500 MHz, DMSO-*d*6): Major δ: 2.46–2.51 (m, 2H, 2CH), 2.69–2.72 (m, 4H, 2CH_2_Ar), 3.05–3.15 (m, 2H, 2CH), 3.52–3.57 (m, 4H, 2CH_2_N), 3.68 (s, 6H, OCH_3_), 4.25 (dd, J = 4.1 Hz, J = 9.1 Hz, 2H, 2CH), 6.79–6.89 (m, 4H, Ar), 7.05–7.09 (m, 4H, Ar); Minor δ: 2.56–2.60 (m, 2H, 2CH), 2.69–2.72 (m, 4H, 2CH_2_Ar), 3.05–3.15 (m, 2H, 2CH), 3.52–3.57 (m, 4H, 2CH_2_N), 3.67 (s, 6H, OCH_3_), 3.96 (dd, J = 4.4 Hz, J = 9.1 Hz, 2H, 2CH), 6.79–6.89 (m, 4H, Ar), 7.05–7.09 (m, 4H, Ar); ^13^C NMR (125 MHz, DMSO-*d*6, δ ppm): 32.1, 32.2, 35.1, 35.2, 36.4, 36.5, 40.2, 40.3, 55.4, 114.3, 114.4, 129.9, 130.1, 130.2, 130.3, 158.3, 175.1, 175.3, 176.0, 176.7; HRMS (ESI) calcd for C_26_H_28_N_2_O_6_S [M + H]^+^: 497.1742; found: 497.1744.

## 4. Conclusions

As a result of the study:

1. We established that the reaction of thioacetamide and N-arylmaleimides proceeded with the formation of three different products depending on the process conditions.
2. We demonstrated that tricyclic bridged compounds—2-(4-methyl-1,3,6-trioxo-2-aryloctahydro-1*H*-4,7-epithiopyrrolo[3,4-c]pyridin-7-yl)-N-arylacetamides **1** were formed by keeping a mixture of starting compounds of thioacetamide and N-arylmaleimide at a molar ratio of 1:2 at 50 °C in dioxane for 10–12 h. The reaction proceeded stereoselectively with the formation of exoisomers.
3. When the reaction was performed heated in dioxane, 2-(4-methyl-1,3,6-trioxo-2-aryl-2,3,5,6-tetrahydro-1*H*-pyrrolo[3,4-c]pyridin-7-yl)-N-arylacetamides **2** were formed as a result of the elimination of a hydrogen sulfide molecule from compounds **1**. Moreover, the batch addition of N-arylmaleimide to a hot solution of thioacetamide in dioxane was optimal.
4. 2-(4-Methyl-1,3,6-trioxo-2-aryl-2,3,5,6-tetrahydro-1*H*-pyrrolo[3,4-c]pyridin-7-yl)-N-arylacetamides **2** could be obtained by refluxing 2-(4-methyl-1,3,6-trioxo-2-aryloctahydro-1*H*-4,7-epithiopyrrolo[3,4-c]pyridin-7-yl)-N-arylacetamides **1** in dioxane, in acetic acid or in methanol in the presence of sodium methoxide, which is a new method for the synthesis of a condensed pyrrolo[3,4-c]pyridine system.
5. When N-substituted maleimide was added in portions to a hot solution of thioacetamide in acetic acid 3,3′-thiobis(1-arylpyrrolidine-2,5-diones) **3a**–**e** and 3,3′-thiobis(1-alkylpyrrolidine-2,5-diones) **3f**–**h** were formed. This is a new method for obtaining such compounds. In the case of N-arylmaleimides and N-methylmaleimide, the reaction proceeded stereoselectively with the formation of a mixture of R,R- and S,S-enantiomers.

## Figures and Tables

**Table 1:** Conditions for the reaction of N-arylmaleimides with thioacetamide and the product composition.

Solvent	Time, h	Temperature	Products, % ***			
			1a	2a	3a	Unidentified and Starting Compounds
CHCl_3_	5	reflux	Low conversion of starting compounds			
CH_3_OH	5	reflux				
(CH_3_)_2_CHOH	3	reflux	36	14	5	45
	6	reflux *	69	5	6	20
dioxane	5	r.t.	Low conversion of starting compounds			
	1	reflux	10	18	1	71
	3	reflux	20	38	2	40
	6	reflux *	5	85	2	8
	12	50 °C	87	-	-	13
dioxane + AcOH	5	reflux	-	80	15	5
AcOH	3	reflux	-	17	45	38
	7	reflux *	-	12	73	15
THF	9	r.t.	25	-	-	75
CH_3_COOC_2_H_5_	8–9	r.t.	24	-	-	76
CH_3_CN	12	r.t.	20	8	6	76
C_2_H_5_OH	4	reflux **	-	25	-	75

* Maleimide was added in several portions; ** Catalytic amount of triethylamine was added; *** According to LC MS Reaction Mass Monitoring.

**Table 2:** Crystal data and structure refinement for **3b**.

Empirical Formula	C_22_H_20_N_2_O_4_S·C_2_H_6_OS
Formula weight	486.59
Temperature, K	100(2)
Wavelength, Å	0.71073
Crystal system	Triclinic
Space group	P 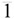
Unit cell dimensions	
a, Å	5.6417(2)
b, Å	13.7700(5)
c, Å	15.1459(6)
α, °	92.2380(10)
β, °	95.0400(10)
γ, °	95.8090(10)
Volume, Å^3^	1164.69(8)
Z	2
Density (calcd.), g•cm^−3^	1.387
Absorption coefficient, mm^−1^	0.268
F(000)	512
Crystal size, mm	0.53 × 0.38 × 0.30
θ range for data collection, °	2.058–34.981
Index ranges	−9 <= h <= 9
	−22 <= k <= 22
	−24 <= l <= 24
Reflections	
Collected	60,803
Independent [R_int_]	10230 [0.1044]
Observed (with I > 2σ(I))	6257
Completeness to θ_max_/θ_full_	0.999/0.999
Data/restraints/parameters	10230/0/302
Goodness-of-fit on F2	1.026
R1/wR2 indices for I > 2σ(I)	0.0683/0.1208
R1/wR2 indices (all data)	0.1329/0.1463
Δρ(ē)_max_/Δρ(ē)_min_, ē•Å^−3^	0.501/−0.674

## Data Availability

Not appliable.

## Supplementary Materials

The following supporting information can be downloaded at: https://www.mdpi.com/article/10.3390/molecules27248800/s1, Figures S1–S14: 1H, 13C NMR and HPLC/MS spectra of 1a–e; Figures S15–S32: 1H, 13C NMR and HPLC/MS spectra of 2a–f; Figures S33–S36 and S38–S58: 1H, 13C NMR and HPLC/MS spectra of 3a–h; Figure S37, Tables S1–S3: X-ray structural analysis data of **3b**.

## Sample Availability

Samples of the compounds are available from the authors.
