# Supplementary material for: New Aspects of the Reaction of Thioacetamide and N-Substituted Maleimides
Source: Molecules. 2022 Dec 12;27(24):8800. doi: 10.3390/molecules27248800 (PMC9785890; doi:10.3390/molecules27248800)
Supplement: Supplementary file 1 [file molecules-27-08800-s001.zip › molecules-2039248-supplementary.pdf]

Supplementary Materials

New aspects of the reaction of thioacetamide and N-arylmaleimides

Yulia V. Aseeva[1], Nadezhda V. Stolpovskaya[1*], Dmitriy Yu. Vandyshev[2], Vladimir B. Sulimov[2,3], Mikhail A. Prezent[4], Mikhail E. Minyaev[4], Khidmet S. Shikhaliev[1]

[1] *Department of Organic Chemistry, Faculty of Chemistry, Voronezh State University, Universitetska-ya Sq. 1, 394018 Voronezh, Russia*

[2] *Moscow Center of Fundamental and Applied Mathematics, 119234 Leninskie Gory str., 1, Moscow, Russia*

[3] *Research Computing Center, Lomonosov Moscow State University, Leninskie Gory, 1, building 4, Moscow, 119234, Russia*

[4] *N. D. Zelinsky Institute of Organic Chemistry, Russian Academy of Sciences, 47 Leninsky prosp., 119991 Moscow, Russia*

Correspondence: stolpovskaya@chem.vsu.ru

Contents

**Figure S1:** Data HPLC of compound **1a**	S-4
**Figure S2:** ^{1}H NMR spectrum (500 MHz, DMSO-d$_6$) of compound **1a**	S-5
**Figure S3:** Data HPLC of compound **1b**	S-6
**Figure S4:** ^{1}H NMR spectrum (500 MHz, DMSO-d$_6$) of compound **1b**	S-7
**Figure S5:** ^{13}C NMR spectrum (125 MHz, DMSO-d$_6$) of compound **1b**	S-8
**Figure S6:** Data HPLC of compound **1c**	S-9
**Figure S7:** ^{1}H NMR spectrum (500 MHz, DMSO-d$_6$) of compound **1c**	S-10
**Figure S8:** ^{13}C NMR spectrum (125 MHz, DMSO-d$_6$) of compound **1c**	S-11
**Figure S9:** Data HPLC of compound **1d**	S-12
**Figure S10:** ^{1}H NMR spectrum (500 MHz, DMSO-d$_6$) of compound **1d**	S-13
**Figure S11:** ^{13}C NMR spectrum (125 MHz, DMSO-d$_6$) of compound **1d**	S-14
**Figure S12:** Data HPLC of compound **1e**	S-15
**Figure S13:** ^{1}H NMR spectrum (500 MHz, DMSO-d$_6$) of compound **1e**	S-16
**Figure S14:** ^{13}C NMR spectrum (125 MHz, DMSO-d$_6$) of compound **1e**	S-17
**Figure S15**: Data HPLC of compound **2a**	S-18
**Figure S16**: ^{1}H NMR spectrum (500 MHz, DMSO-d6) of compound **2a**	S-19
**Figure S17**: ^{13}C NMR spectrum (125 MHz, DMSO-d6) of compound **2a**	S-20

**Figure S18**: Data HPLC of compound **2b**	S-21
**Figure S19**: ^{1}H NMR spectrum (500 MHz, DMSO-d6) of compound **2b**	S-22
**Figure S20**: ^{13}C NMR spectrum (125 MHz, DMSO-d6) of compound **2b**	S-23
**Figure S21**: Data HPLC of compound **2c**	S-24
**Figure S22**: ^{1}H NMR spectrum (500 MHz, DMSO-d6) of compound **2c**	S-25
**Figure S23**: ^{13}C NMR spectrum (125 MHz, DMSO-d6) of compound **2c**	S-26
**Figure S24**: Data HPLC of compound **2d**	S-27
**Figure S25**: ^{1}H NMR spectrum (500 MHz, DMSO-d6) of compound **2d**	S-28
**Figure S26**: ^{13}C NMR spectrum (125 MHz, DMSO-d6) of compound **2d**	S-29
**Figure S27**: Data HPLC of compound **2e**	S-30
**Figure S28**: ^{1}H NMR spectrum (500 MHz, DMSO-d6) of compound **2e**	S-31
**Figure S29**: ^{13}C NMR spectrum (125 MHz, DMSO-d6) of compound **2e**	S-32
**Figure S30**: Data HPLC of compound **2f**	S-33
**Figure S31**: ^{1}H NMR spectrum (500 MHz, DMSO-d6) of compound **2f**	S-34
**Figure S32**: ^{13}C NMR spectrum (125 MHz, DMSO-d6) of compound **2f**	S-35
**Figure S33**: Data HPLC of compound **3a**	S-36
**Figure S34**: ^{1}H NMR spectrum (500 MHz, $CDCl_3$) of compound **3a**	S-37
**Figure S35**: ^{1}H NMR spectrum (500 MHz, DMSO-d6) of compound **3a**	S-38
**Figure S36**: ^{13}C NMR spectrum (125 MHz, DMSO-d6) of compound **3a**	S-39
**Figure S37**: Molecular structure of **3b**•DMSO. A probability level for atomic displacement parameters for non-H atoms is set to 50%	S-40
**Table S1.** Crystal data and structure refinement for **3b**.	S-40
**Table S2.** Selected bond lengths for **3b**, Å.	S-41
**Table S3.** Selected angle values for **3b**, °.	S-41
**Figure S38**: Data HPLC of compound **3b**	S-42
**Figure S39**: ^{1}H NMR spectrum (500 MHz, DMSO-d6) of compound **3b**	S-43
**Figure S40**: ^{13}C NMR spectrum (125 MHz, DMSO-d6) of compound **3b**	S-44
**Figure S41**: Data HPLC of compound **3c**	S-45
**Figure S42**: ^{1}H NMR spectrum (500 MHz, $CDCl_3$) of compound **3c**	S-46
**Figure S43**: ^{13}C NMR spectrum (125 MHz, DMSO-d6) of compound **3c**	S-47
**Figure S44**: Data HPLC of compound **3d**	S-48
**Figure S45**: ^{1}H NMR spectrum (500 MHz, DMSO-d6) of compound **3d**	S-49

**Figure S46**: ^{13}C NMR spectrum (125 MHz, DMSO-d6) of compound **3d**	S-50
**Figure S47**: Data HPLC of compound **3e**	S-51
**Figure S48**: ^{1}H NMR spectrum (500 MHz, DMSO-d6) of compound **3e**	S-52
**Figure S49**: ^{13}C NMR spectrum (125 MHz, DMSO-d6) of compound **3e**	S-53
**Figure S50**: Data HPLC of compound **3f**	S-54
**Figure S51**: ^{1}H NMR spectrum (500 MHz, DMSO-d6) of compound **3f**	S-55
**Figure S52**: ^{13}C NMR spectrum (125 MHz, DMSO-d6) of compound **3f**	S-56
**Figure S53**: Data HPLC of compound **3g**	S-57
**Figure S54**: ^{1}H NMR spectrum (500 MHz, DMSO-d6) of compound **3g**	S-58
**Figure S55**: ^{13}C NMR spectrum (125 MHz, DMSO-d6) of compound **3g**	S-59
**Figure S56**: Data HPLC of compound **3h**	S-60
**Figure S57**: ^{1}H NMR spectrum (500 MHz, DMSO-d6) of compound **3h**	S-61
**Figure S58**: ^{13}C NMR spectrum (125 MHz, DMSO-d6) of compound **3h**	S-62

User Chromatograms

Fragmentor Voltage 191 Collision Energy 0 Ionization Mode ESI

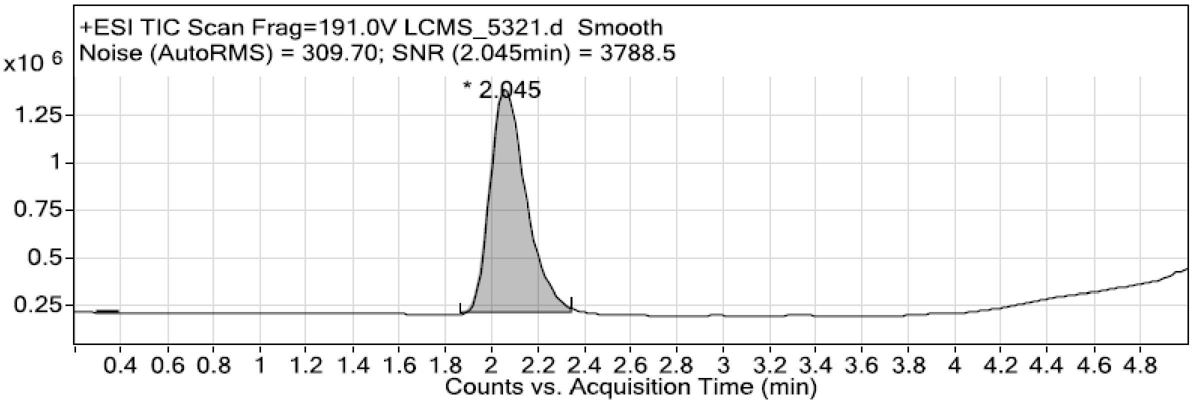

Integration Peak List

Peak	Start	RT	End	Height	Area	Area %	Signal To Noise
1	1.863	2.045	2.343	1173307.92	12879644.72	100	3788.5

Noise Measurements

Noise Type	Signal Definition	Noise Multiplier	Noise Value
Auto-RMS	Height	1	309.7027588

User Spectra

Spectrum Source	Fragmentor Voltage	Collision Energy	Ionization Mode
Peak (1) in "+ TIC Scan Smo"	191	0	ESI

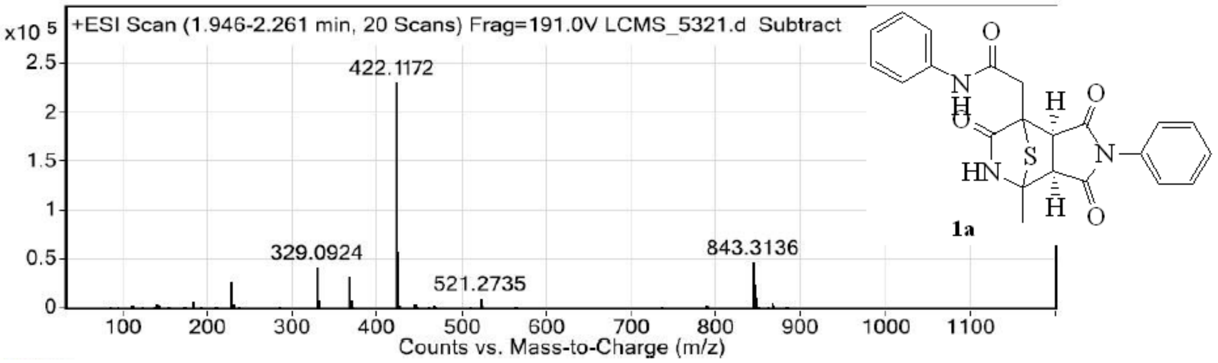

Figure S1: Data HPLC of compound **1a**

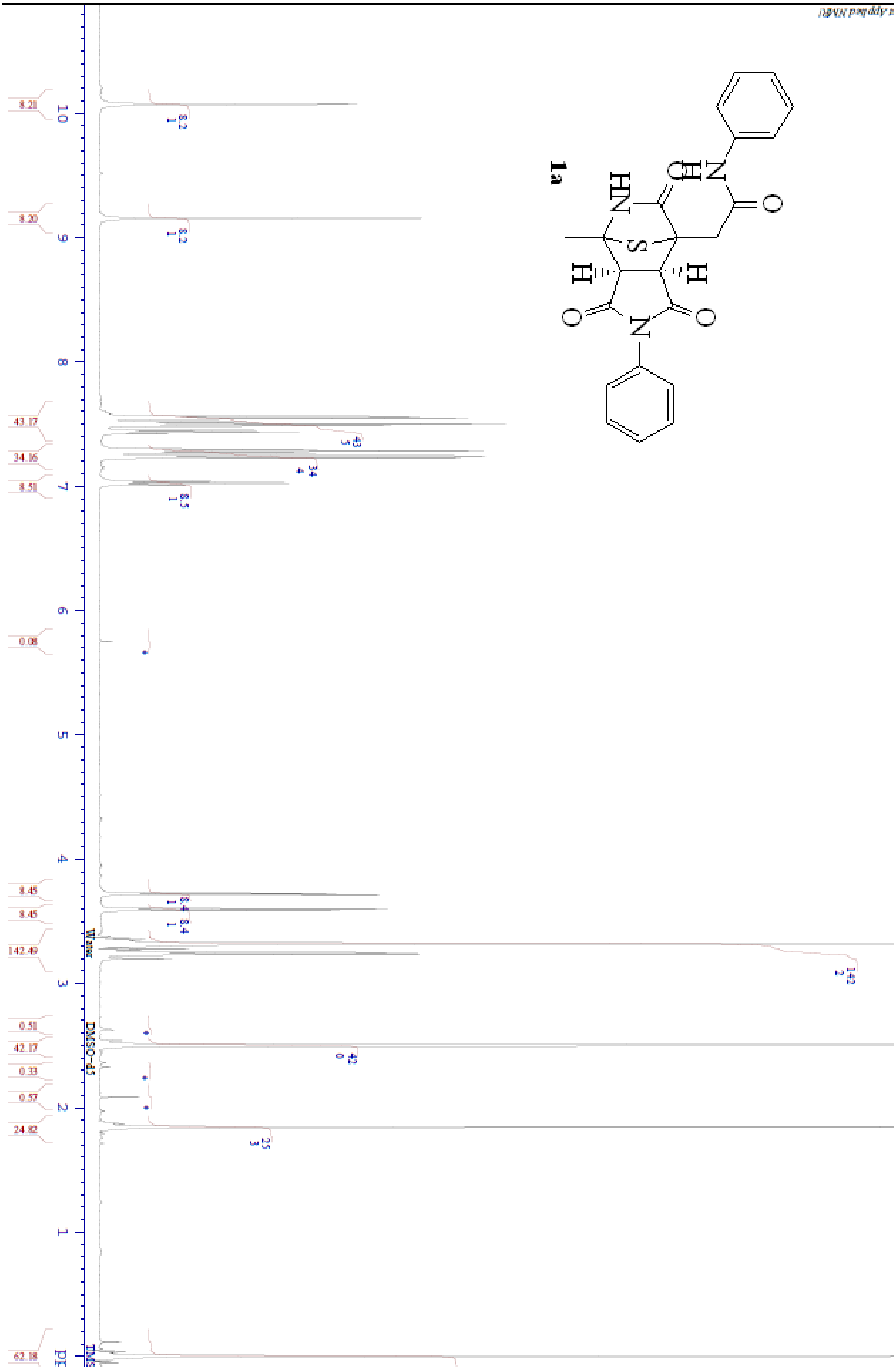

Figure S2: ^{1}H NMR spectrum (500 MHz, DMSO-d6) of compound **1a**

User Chromatograms

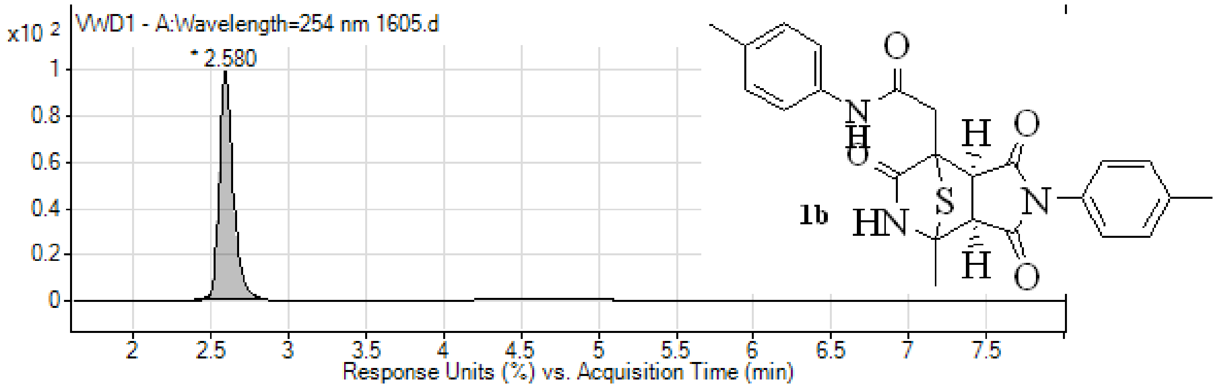

Integration Peak List

Peak	Start	RT	End	Height	Area	Area %
1	2,393	2,58	2,847	594,36	3743,44	100

User Spectra

Fragmentor Voltage	Collision Energy	Ionization Mode
191	0	ESI

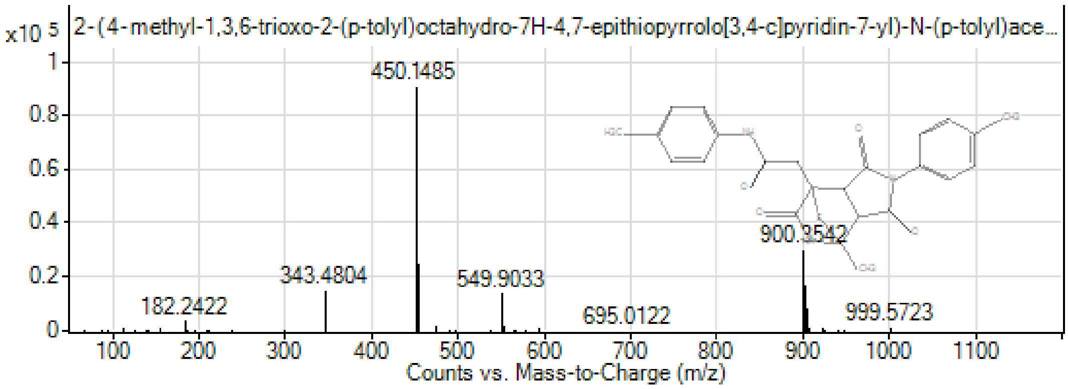

Figure S3: Data HPLC of compound **1b**

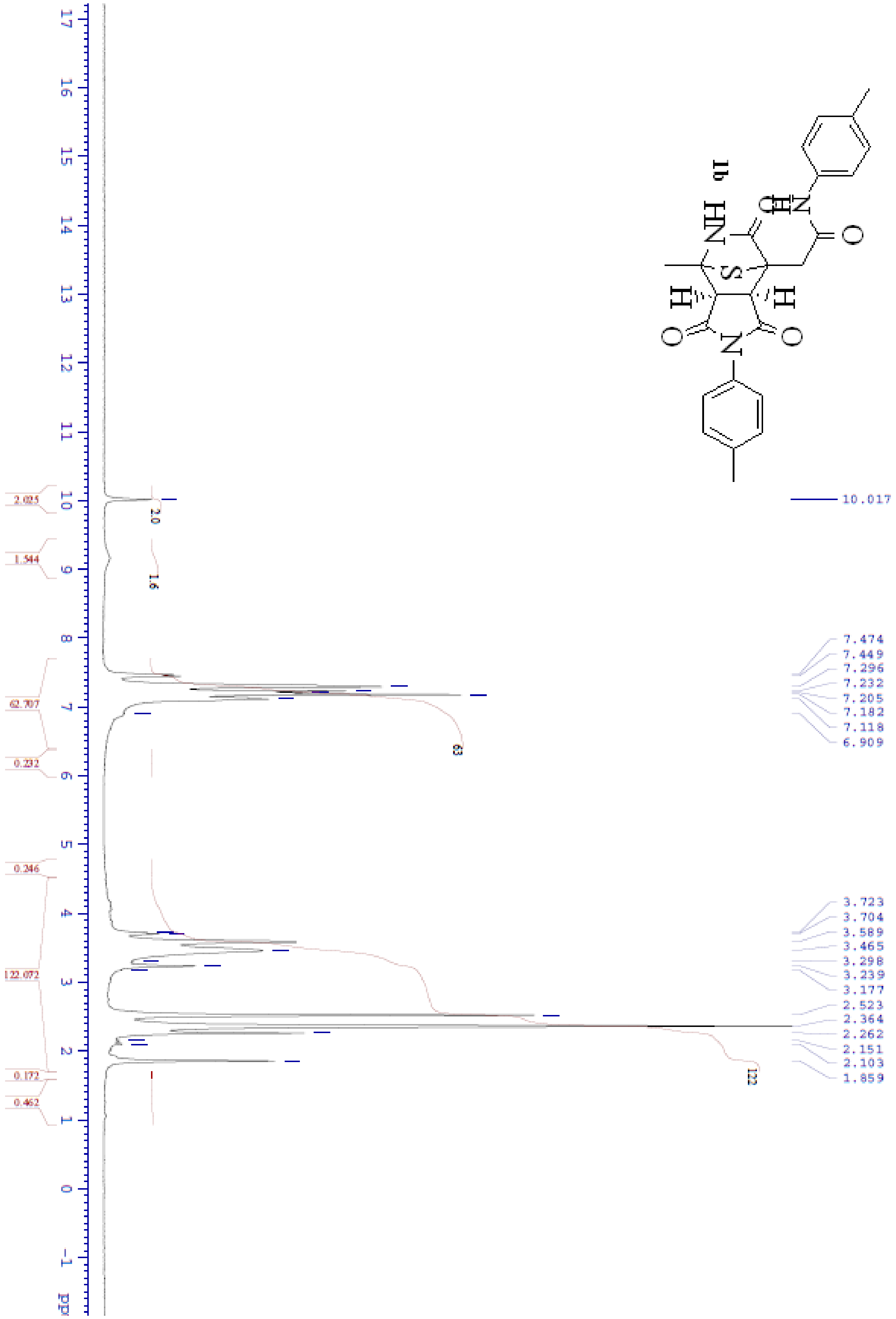

Figure S4: ^{1}H NMR spectrum (500 MHz, DMSO-d6) of compound **1b**

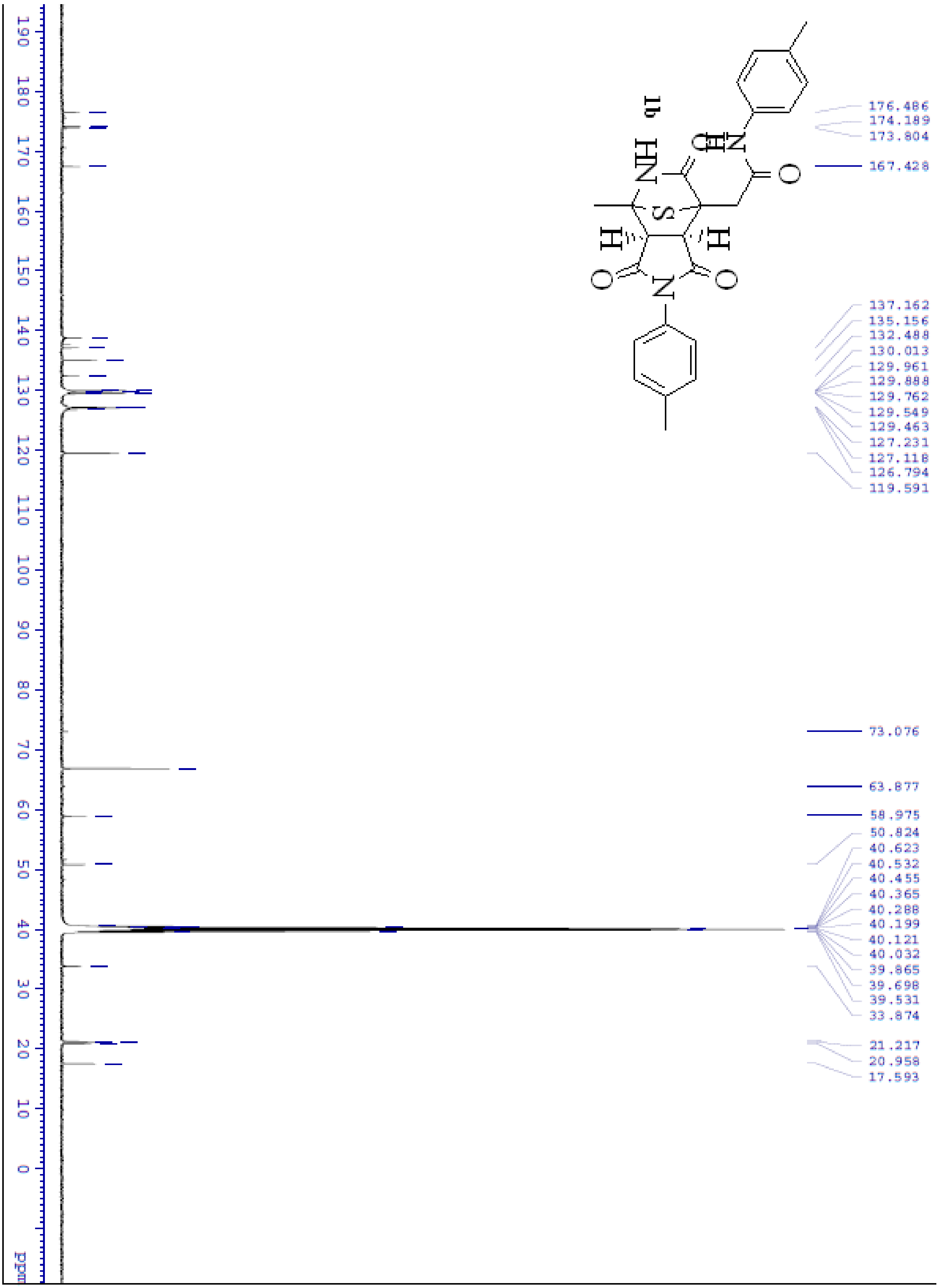

Figure S5: ^{13}C NMR spectrum (125 MHz, DMSO-d6) of compound **1b**

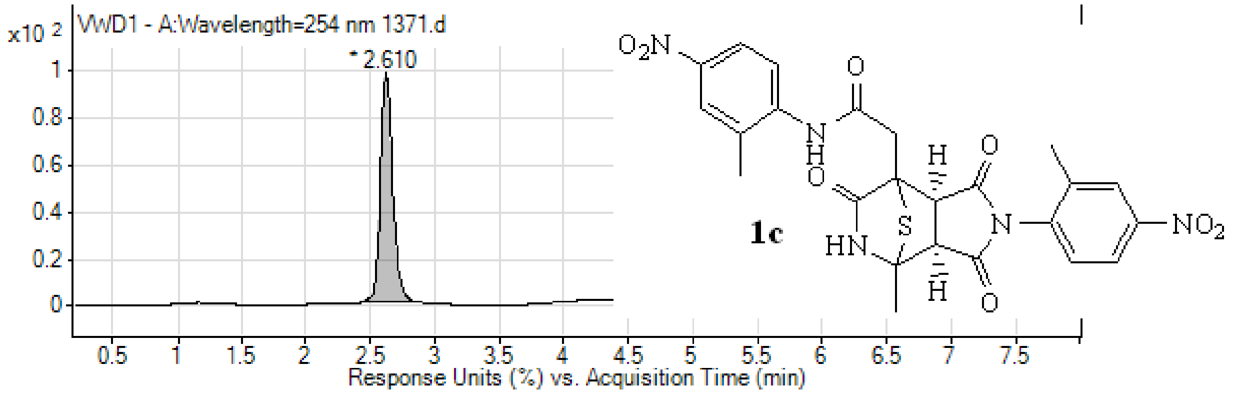

Integration Peak List

Peak	Start	RT	End	Height	Area	Area %
1	2,42	2,61	2,863	186,8	1237,52	100

User Spectra

Fragmentor Voltage	Collision Energy	Ionization Mode
191	0	ESI

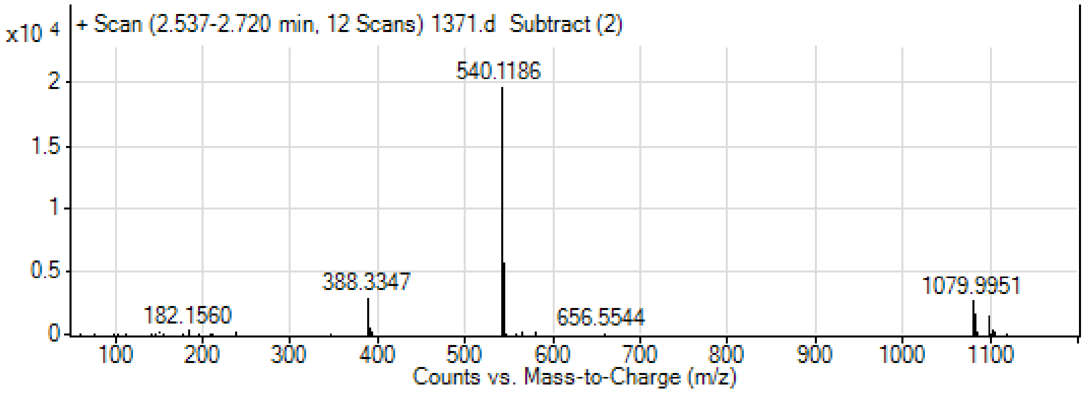

Figure S6: Data HPLC of compound **1c**

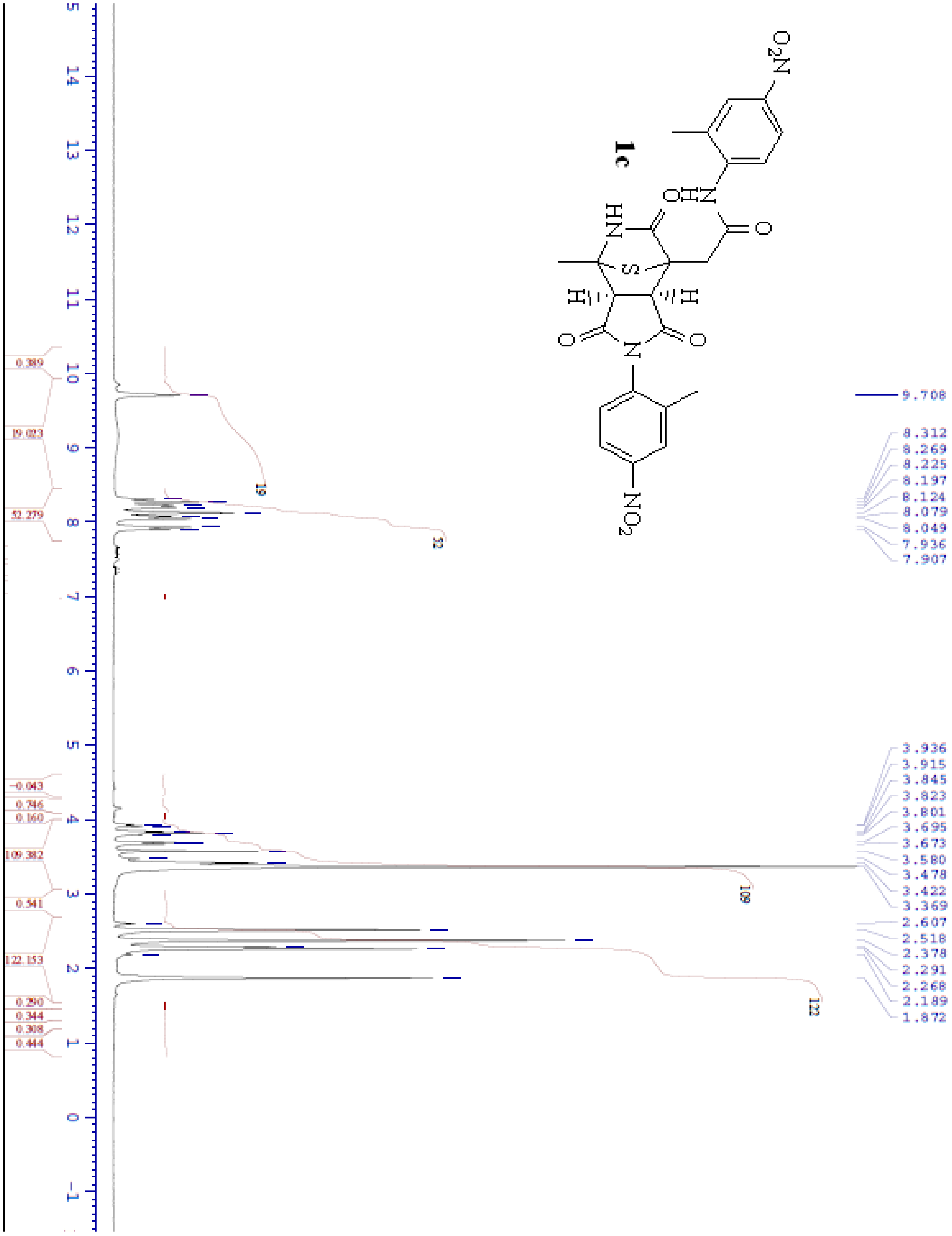

Figure S7: ^{1}H NMR spectrum (500 MHz, DMSO-d6) of compound **1c**

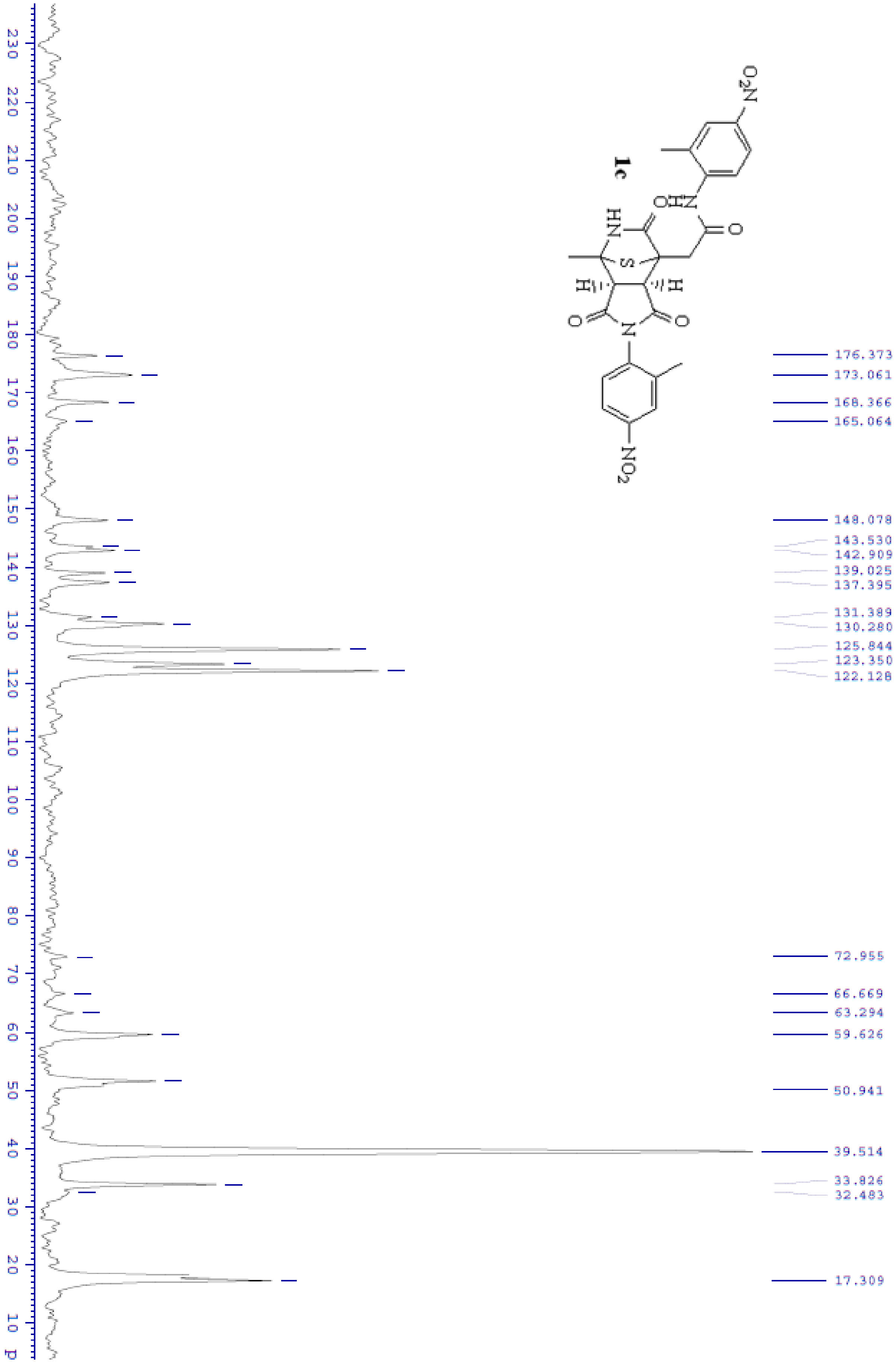

Figure S8: ^{13}C NMR spectrum (125 MHz, DMSO-d6) of compound **1c**

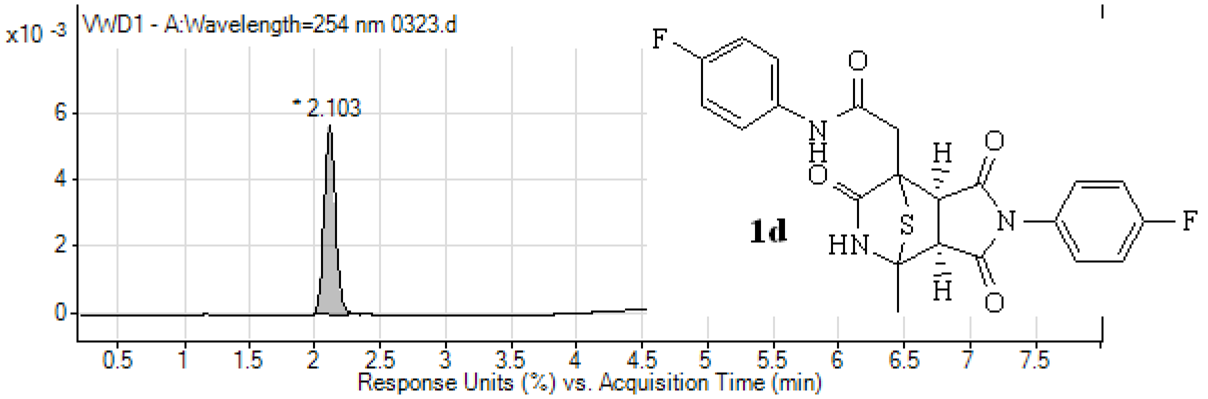

Integration Peak List

Peak	Start	RT	End	Height	Area	Area %
1	1,983	2,103	2,247	330,89	1957,57	100

User Spectra

Fragmentor Voltage	Collision Energy	Ionization Mode
191	0	ESI

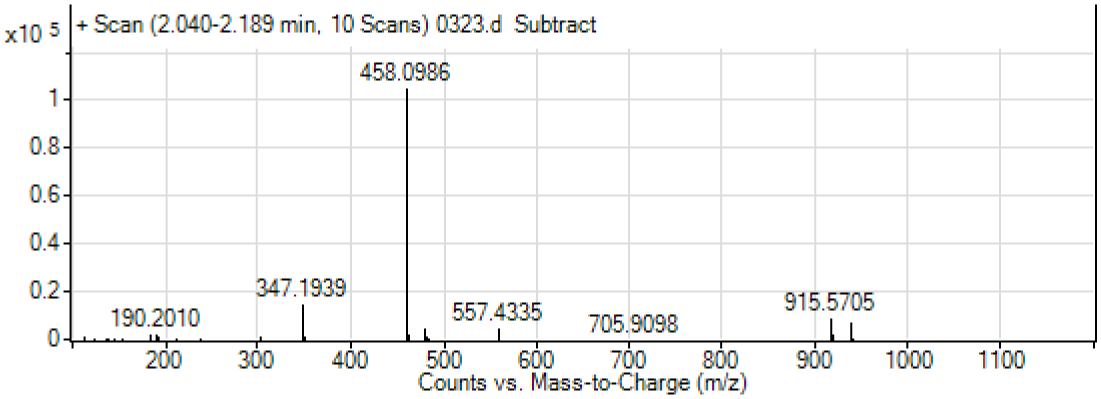

Figure S9: Data HPLC of compound **1d**

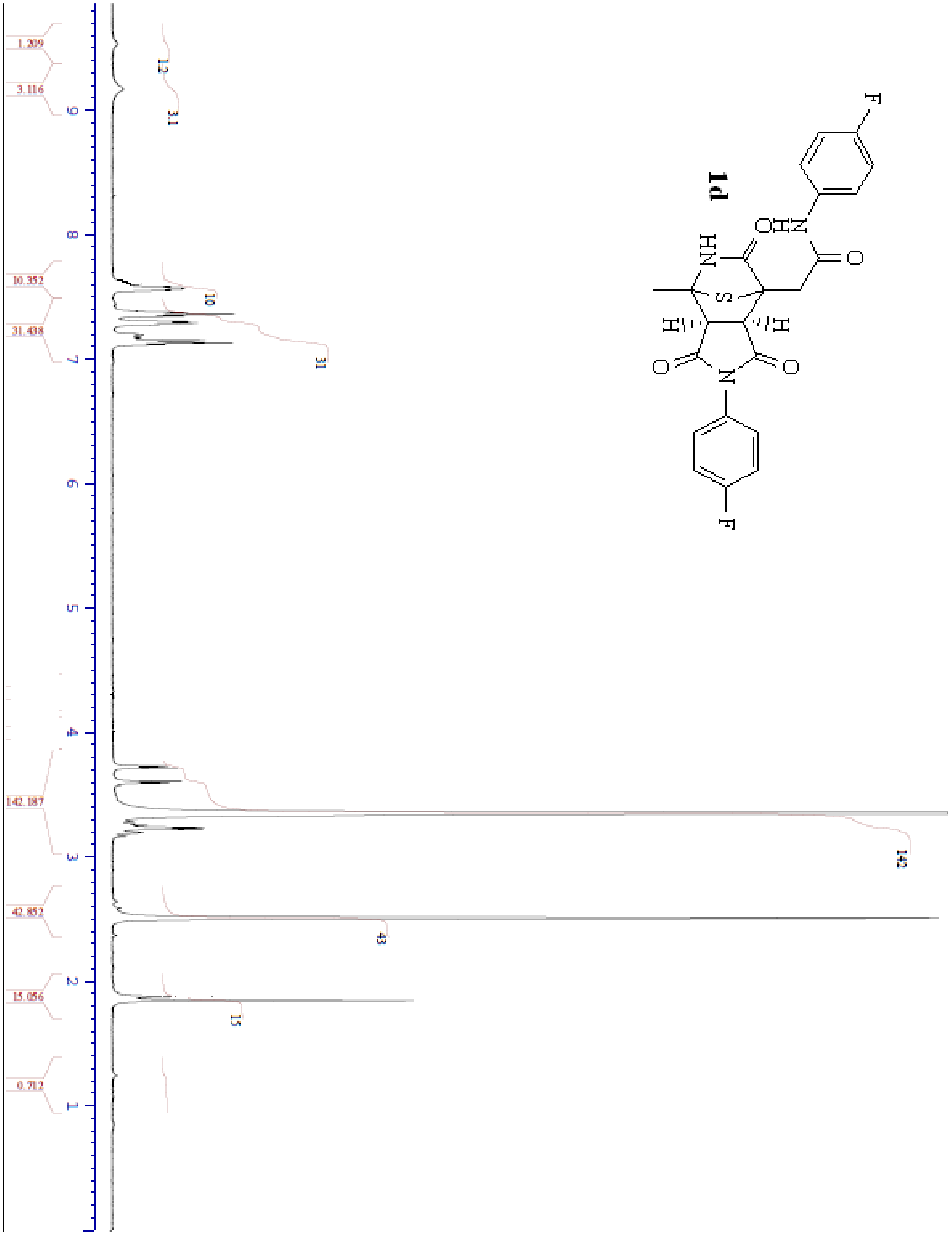

Figure S10: ^{1}H NMR spectrum (500 MHz, DMSO-d6) of compound **1d**

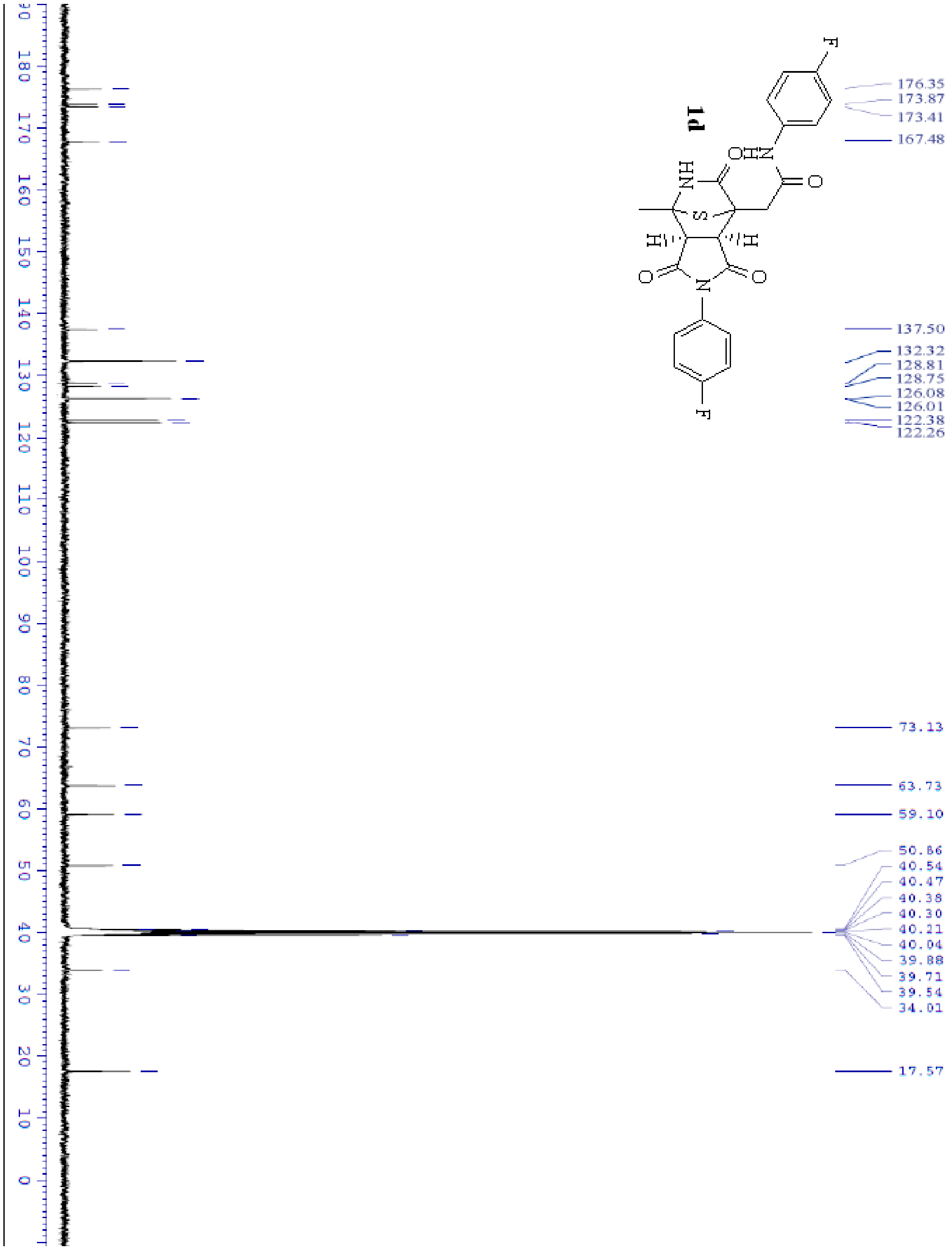

Figure S11: ^{13}C NMR spectrum (125 MHz, DMSO-d6) of compound **1d**

User Chromatograms

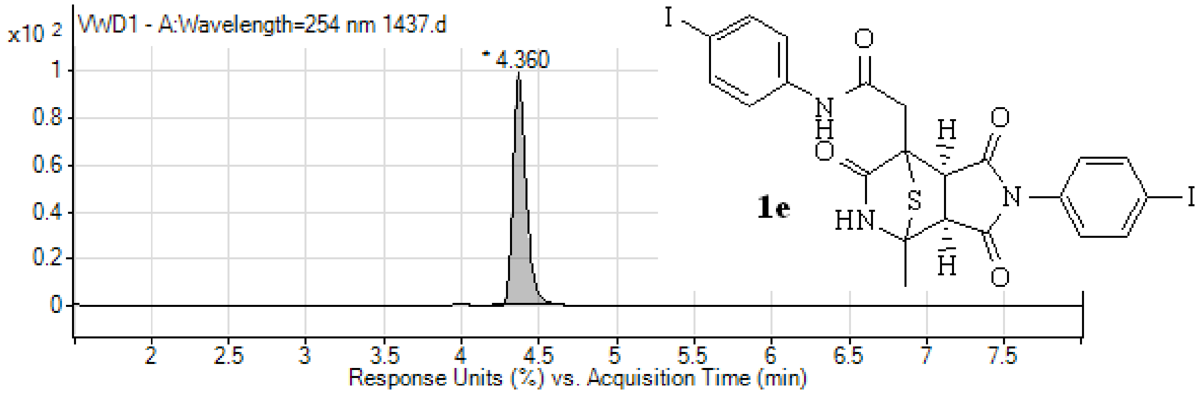

Integration Peak List

Peak	Start	RT	End	Height	Area	Area %
1	4,2	4,36	4,637	921,53	5377,5	100

User Spectra

Fragmentor Voltage	Collision Energy	Ionization Mode
191	0	ESI

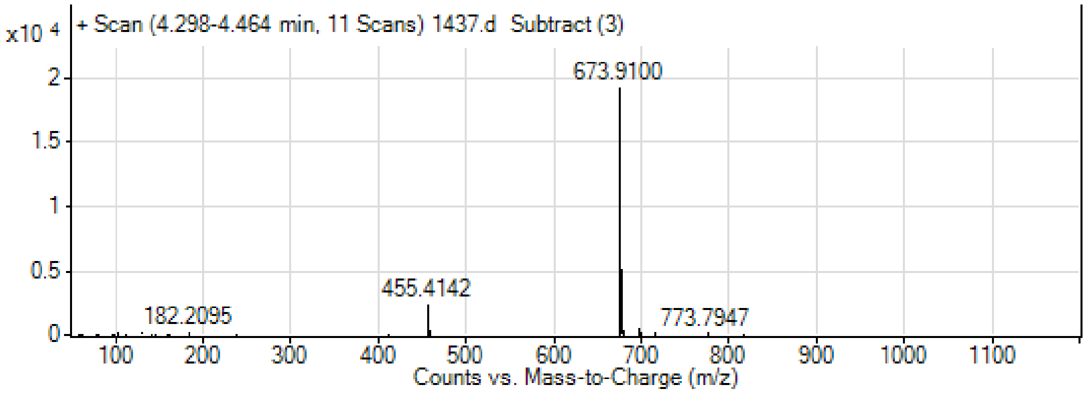

Figure S12: Data HPLC of compound **1e**

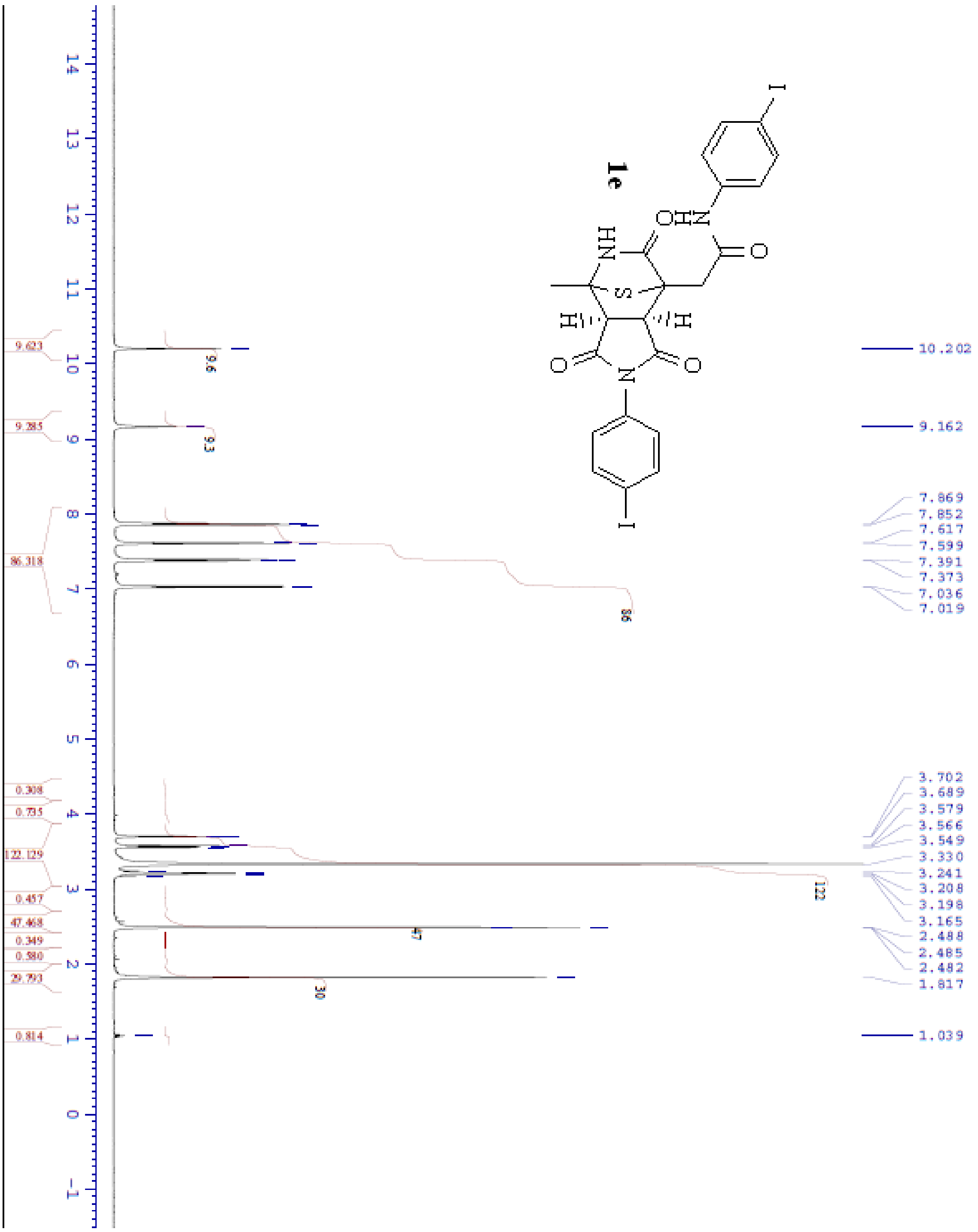

Figure S13: ^{1}H NMR spectrum (500 MHz, DMSO-d6) of compound **1e**

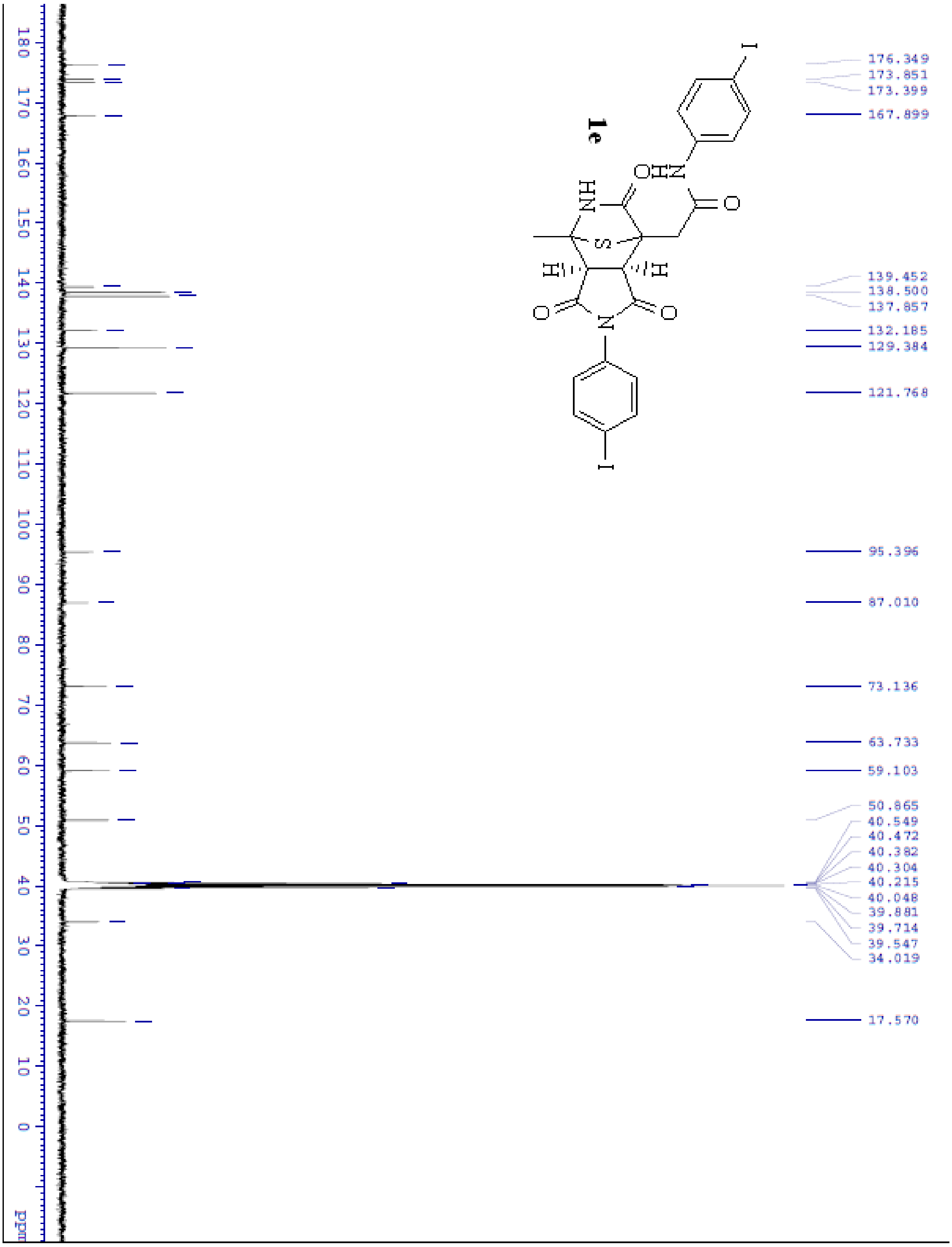

Figure S14: ^{13}C NMR spectrum (125 MHz, DMSO-d6) of compound **1e**

User Chromatograms

Fragmentor Voltage 191 Collision Energy 0 Ionization Mode ESI

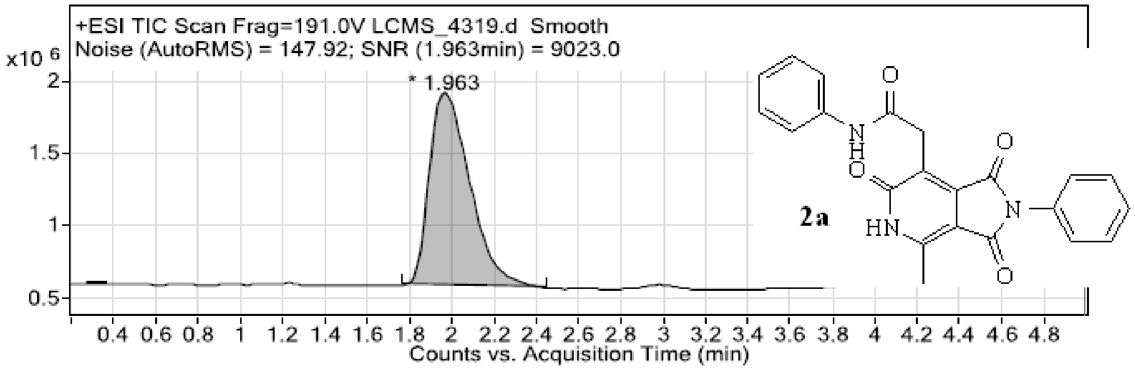

Integration Peak List

Peak	Start	RT	End	Height	Area	Area %	Signal To Noise
1	1.764	1.963	2.444	1334703.11	17856755.04	100	9023

Noise Measurements

Noise Type	Signal Definition	Noise Multiplier	Noise Value
Auto-RMS	Height	1	147.9220123

Noise Regions

Start	End
0.273183333	0.373183333

User Spectra

trum Source (1) in "+ TIC Scan Smo" Fragmentor Voltage 191 Collision Energy 0 Ionization Mode ESI

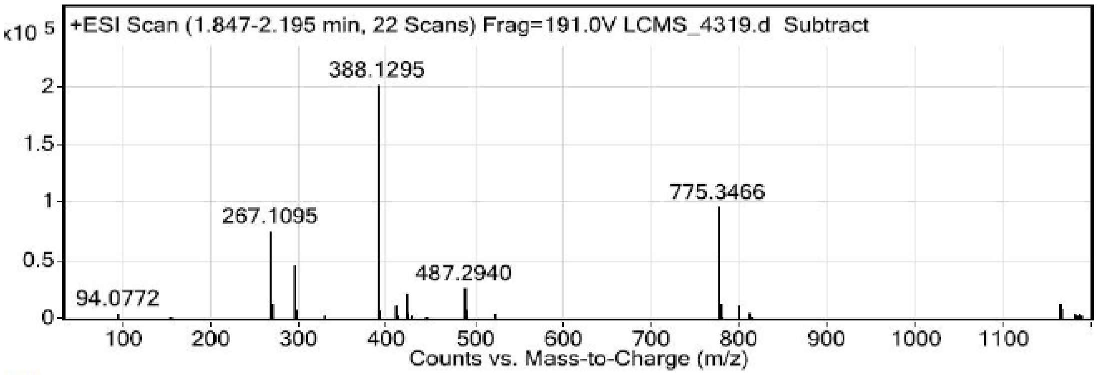

Figure S15: Data HPLC of compound **2a**

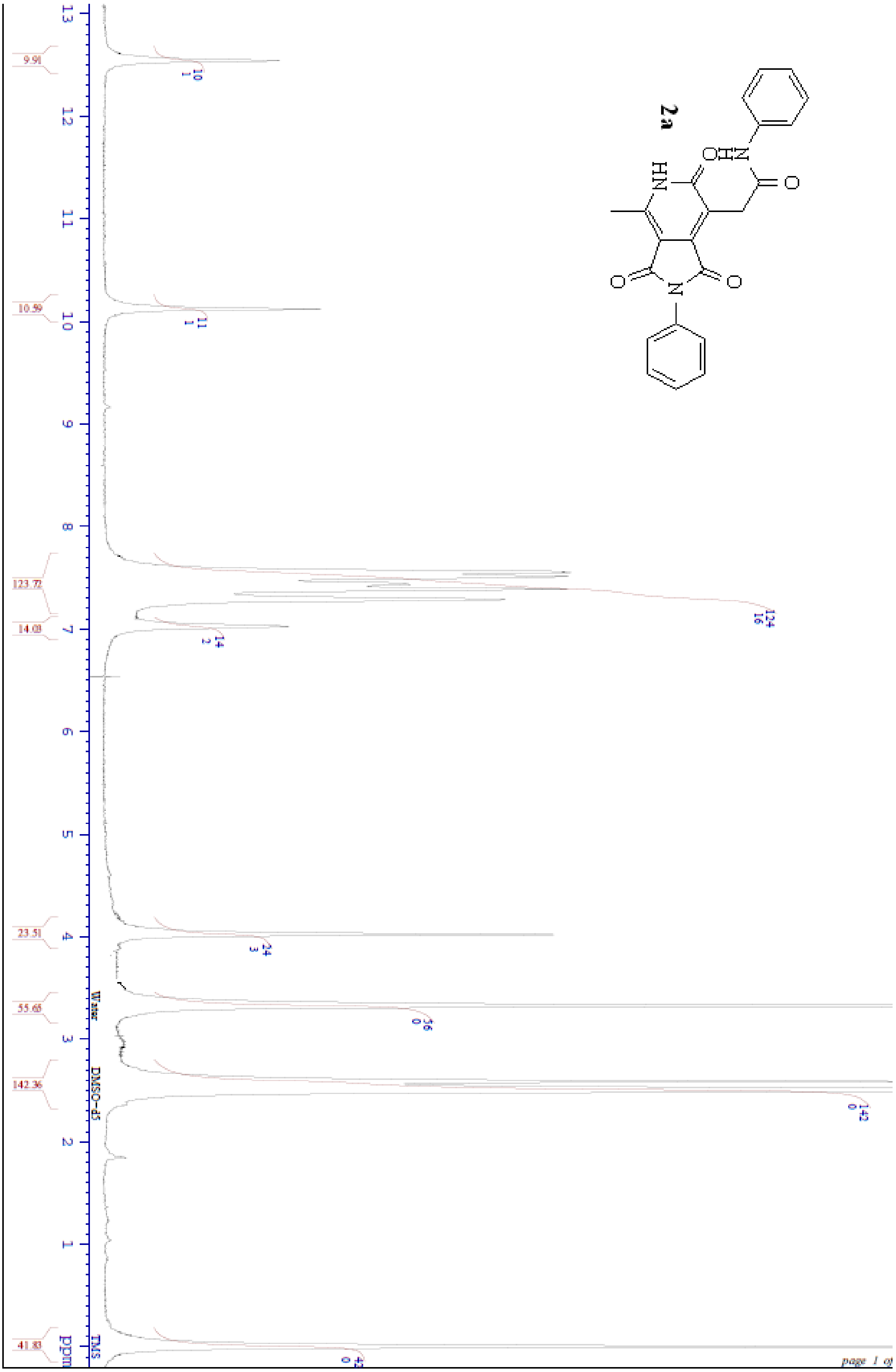

Figure S16: ^{1}H NMR spectrum (500 MHz, DMSO-d6) of compound **2a**

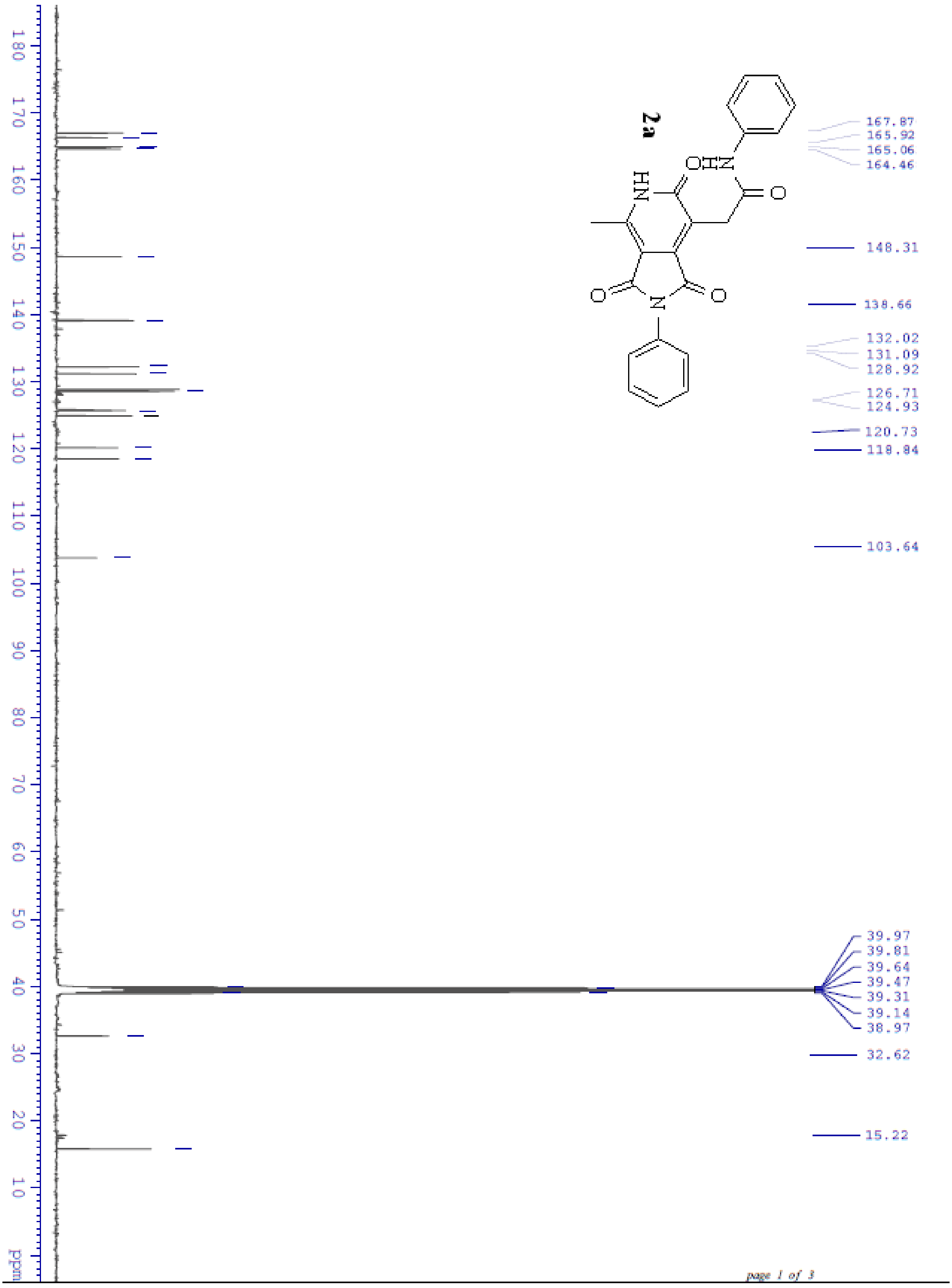

Figure S17: ^{13}C NMR spectrum (125 MHz, DMSO-d6) of compound **2a**

User Chromatograms

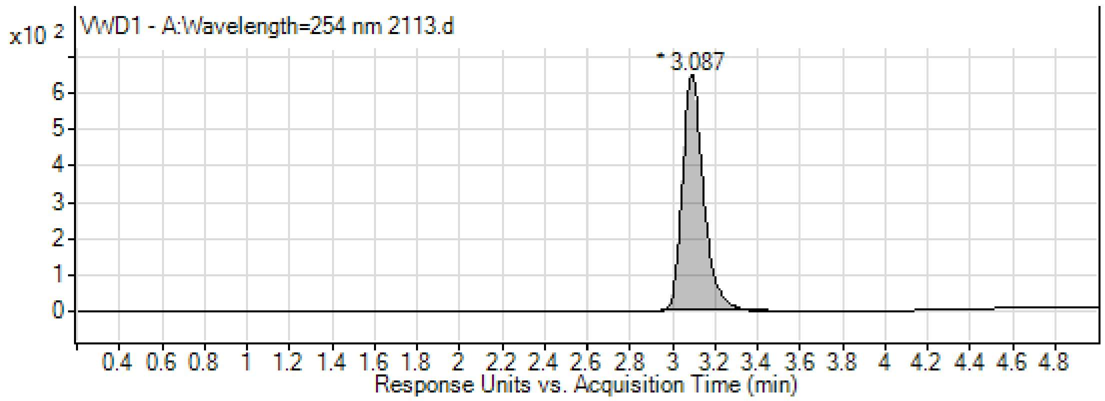

Integration Peak List

Peak	Start	RT	End	Height	Area	Area %
1	2,94	3,087	3,447	652,22	4548,14	100

User Spectra

Fragmentor Voltage	Collision Energy	Ionization Mode
191	0	ESI

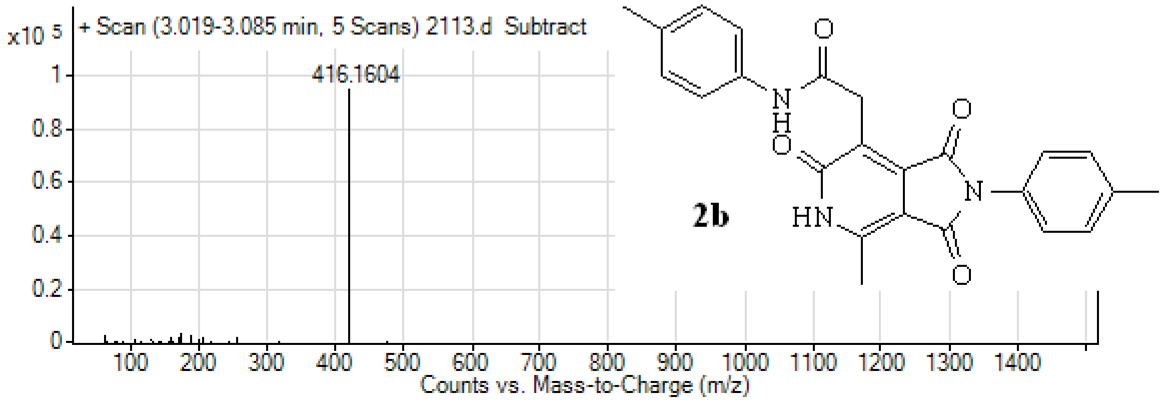

Figure S18: Data HPLC of compound **2b**

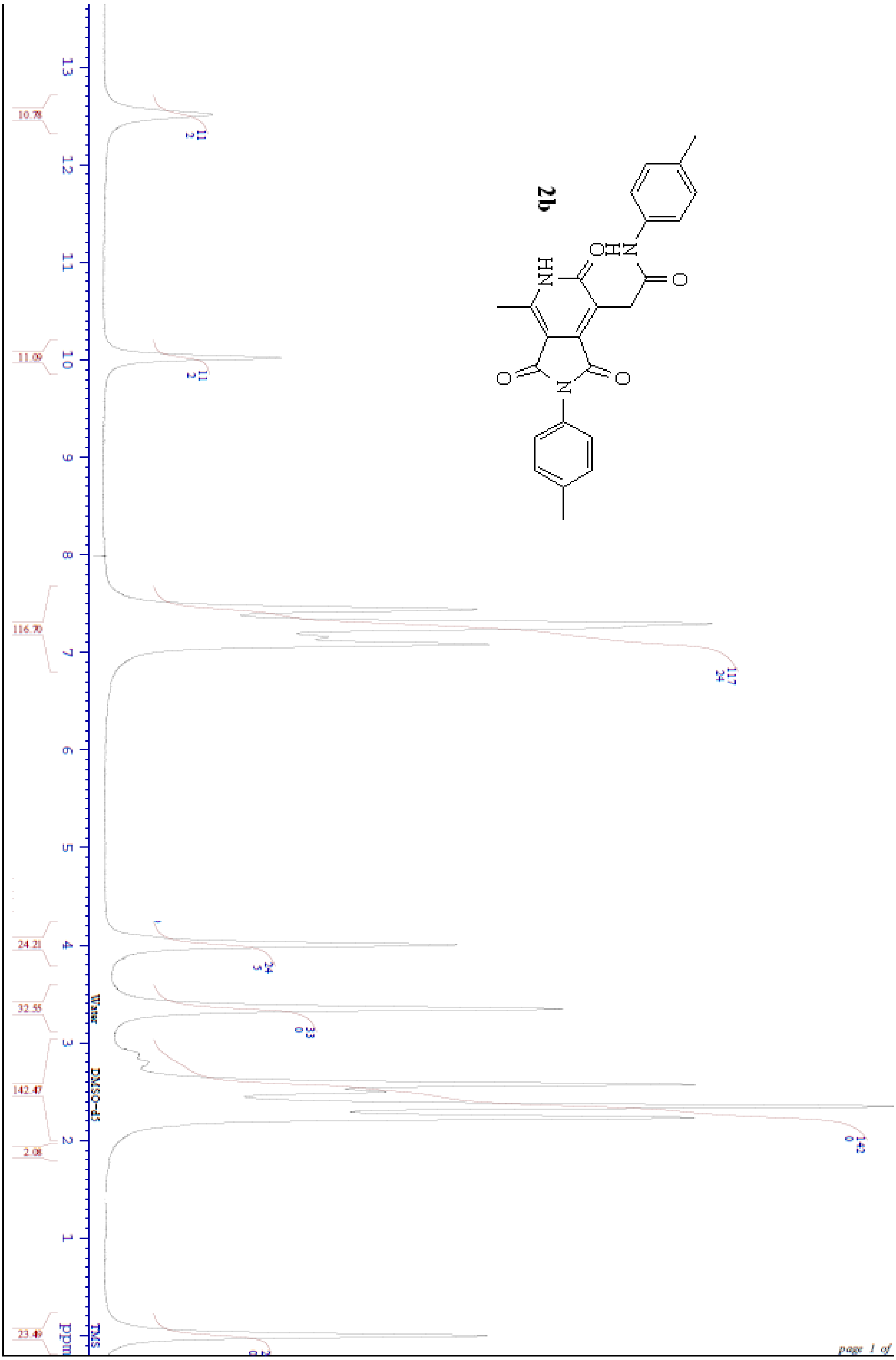

Figure S19: ^{1}H NMR spectrum (500 MHz, DMSO-d6) of compound **2b**

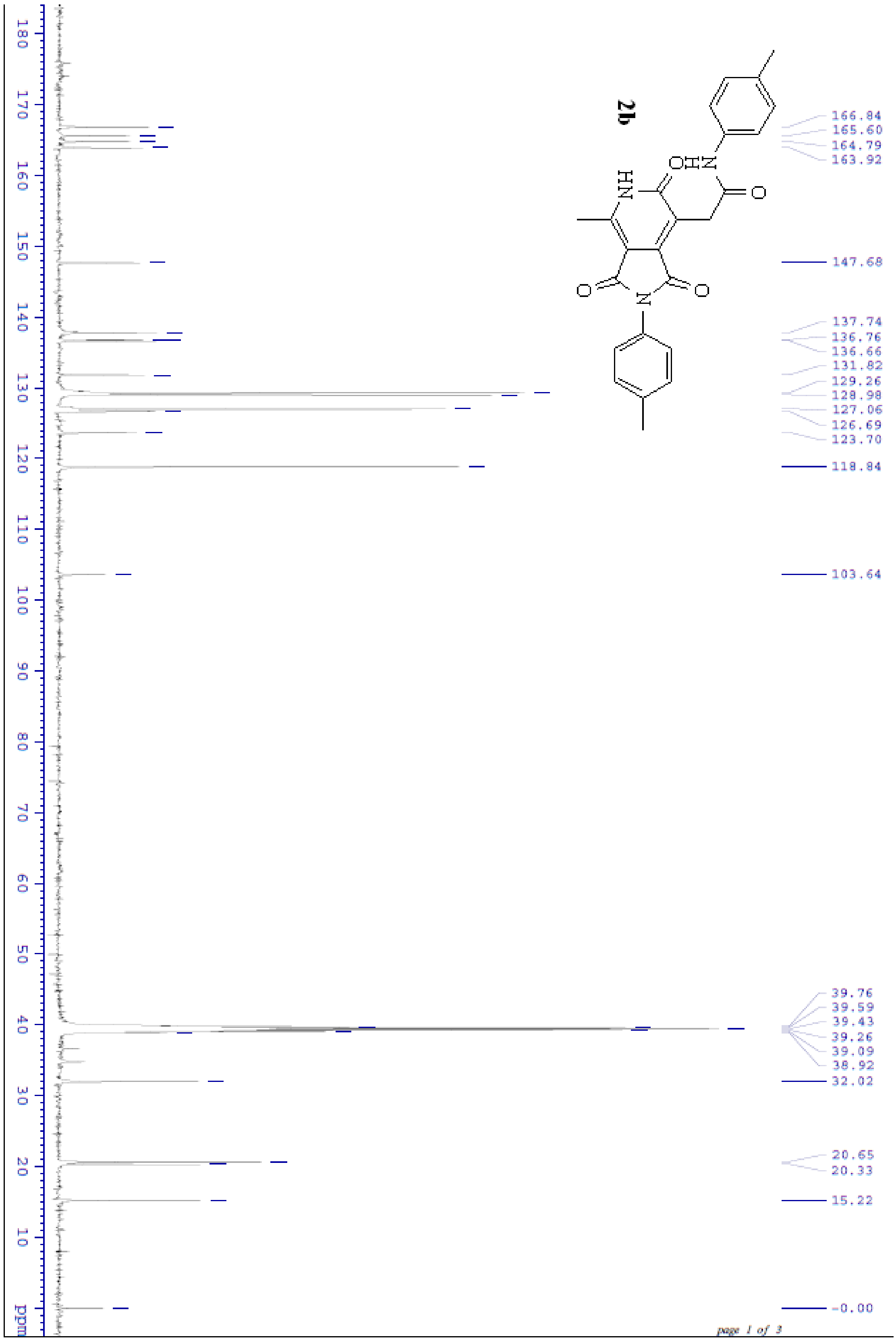

Figure S20: ^{13}C NMR spectrum (125 MHz, DMSO-d6) of compound **2b**

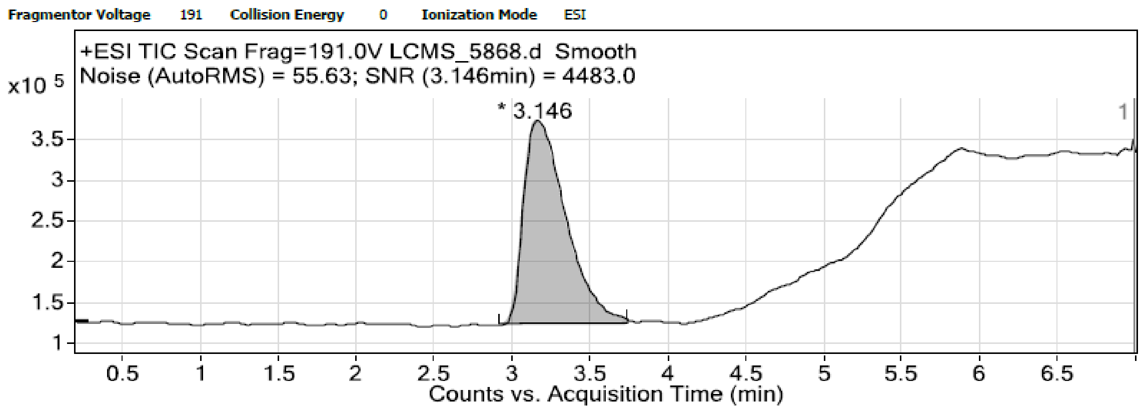

User Chromatogram Peak List

RT	Height	Height %	Area	Area %	Area Sum %	S/N	Symmetry	Width
3.146	249392.82	100	4749675.21	100	100	4483	2.82	0.829

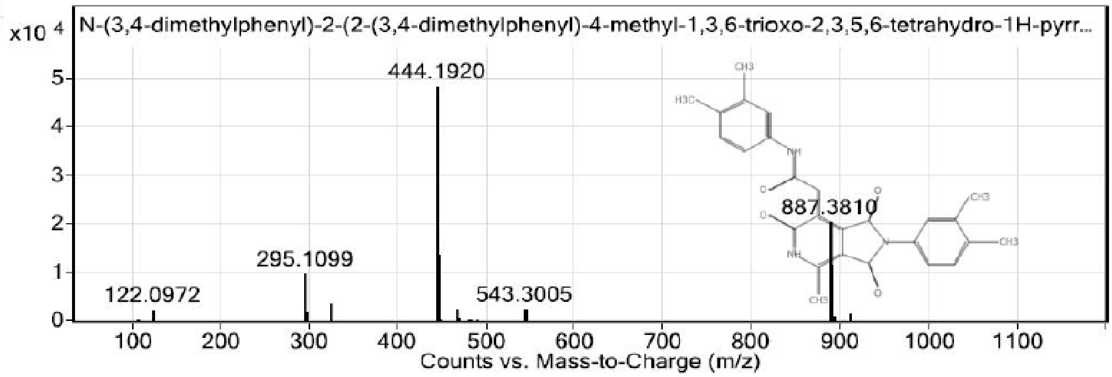

Figure S21: Data HPLC of compound **2c**

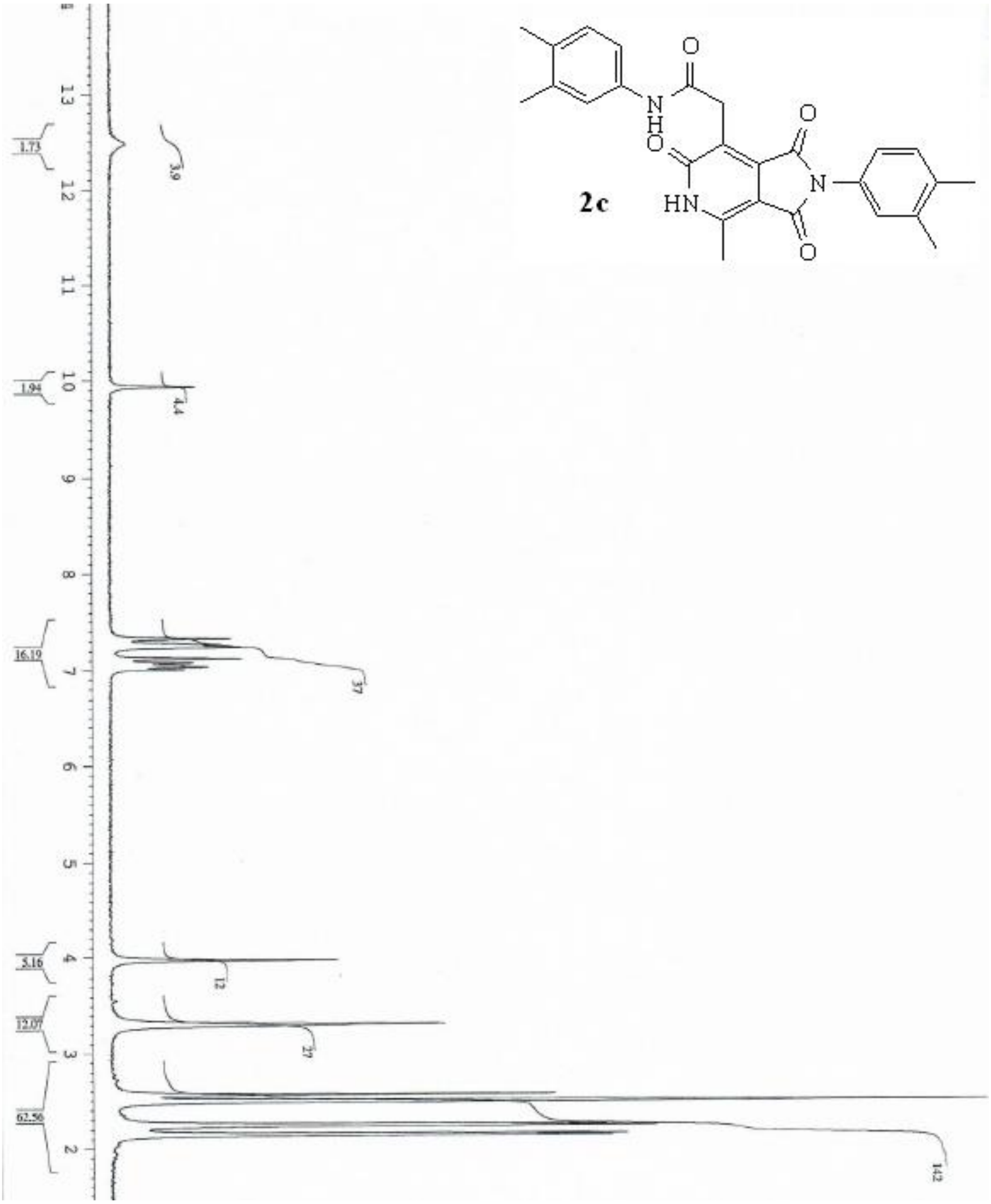

Figure S22: ^{1}H NMR spectrum (500 MHz, DMSO-d6) of compound **2c**

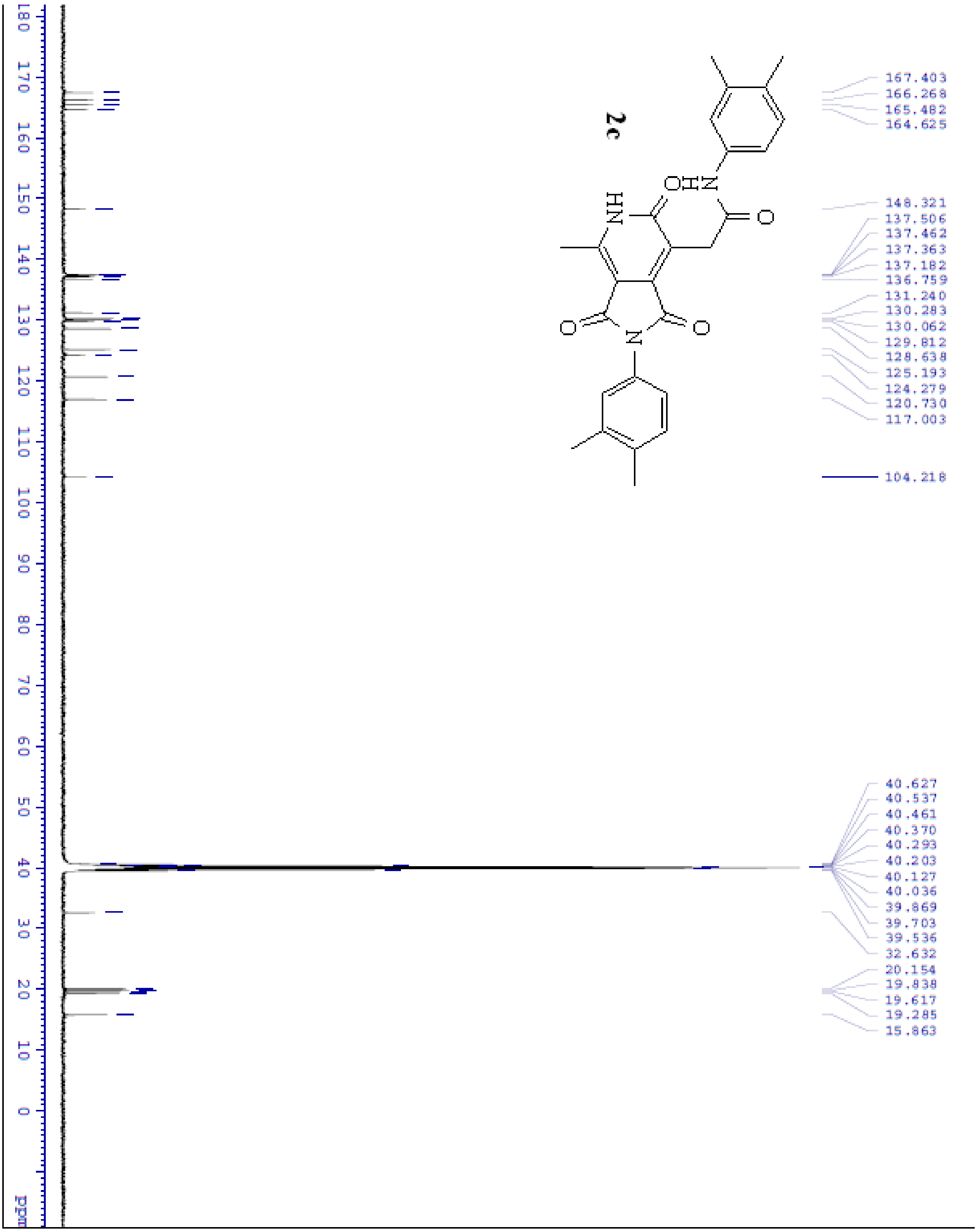

Figure S23: ^{13}C NMR spectrum (125 MHz, DMSO-d6) of compound **2c**

User Chromatograms

Fragmentor Voltage 191 **Collision Energy** 0 **Ionization Mode** ESI

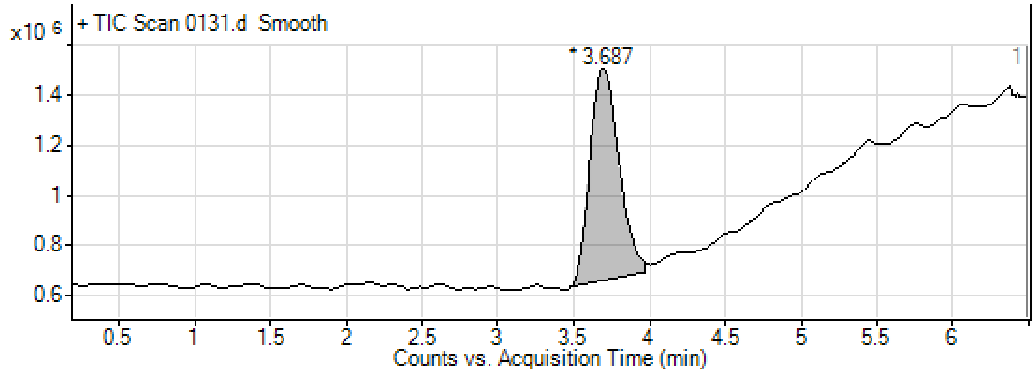

Integration Peak List

Peak	Start	RT	End	Height	Area	Area %
1	3,472	3,687	3,969	854435,71	11440062,31	100

User Spectra

Fragmentor Voltage 191 **Collision Energy** 0 **Ionization Mode** ESI

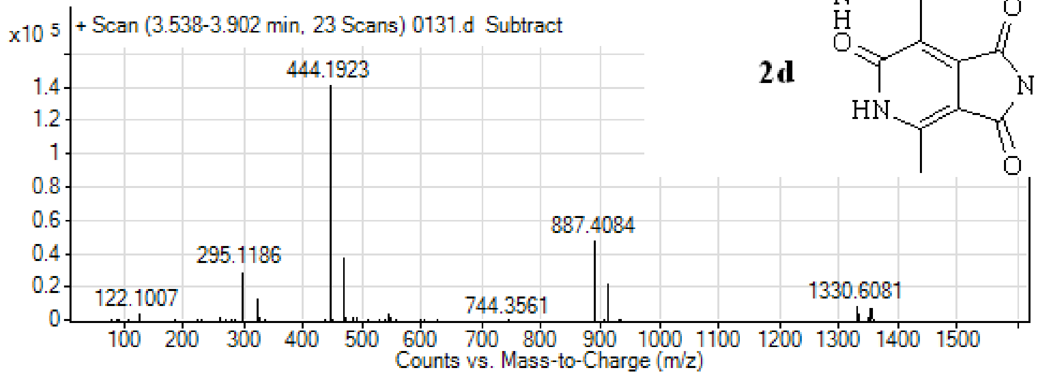

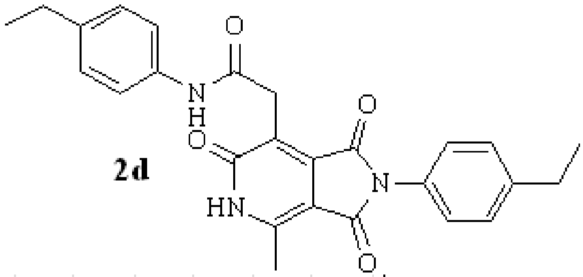

2d

Figure S24: Data HPLC of compound **2d**

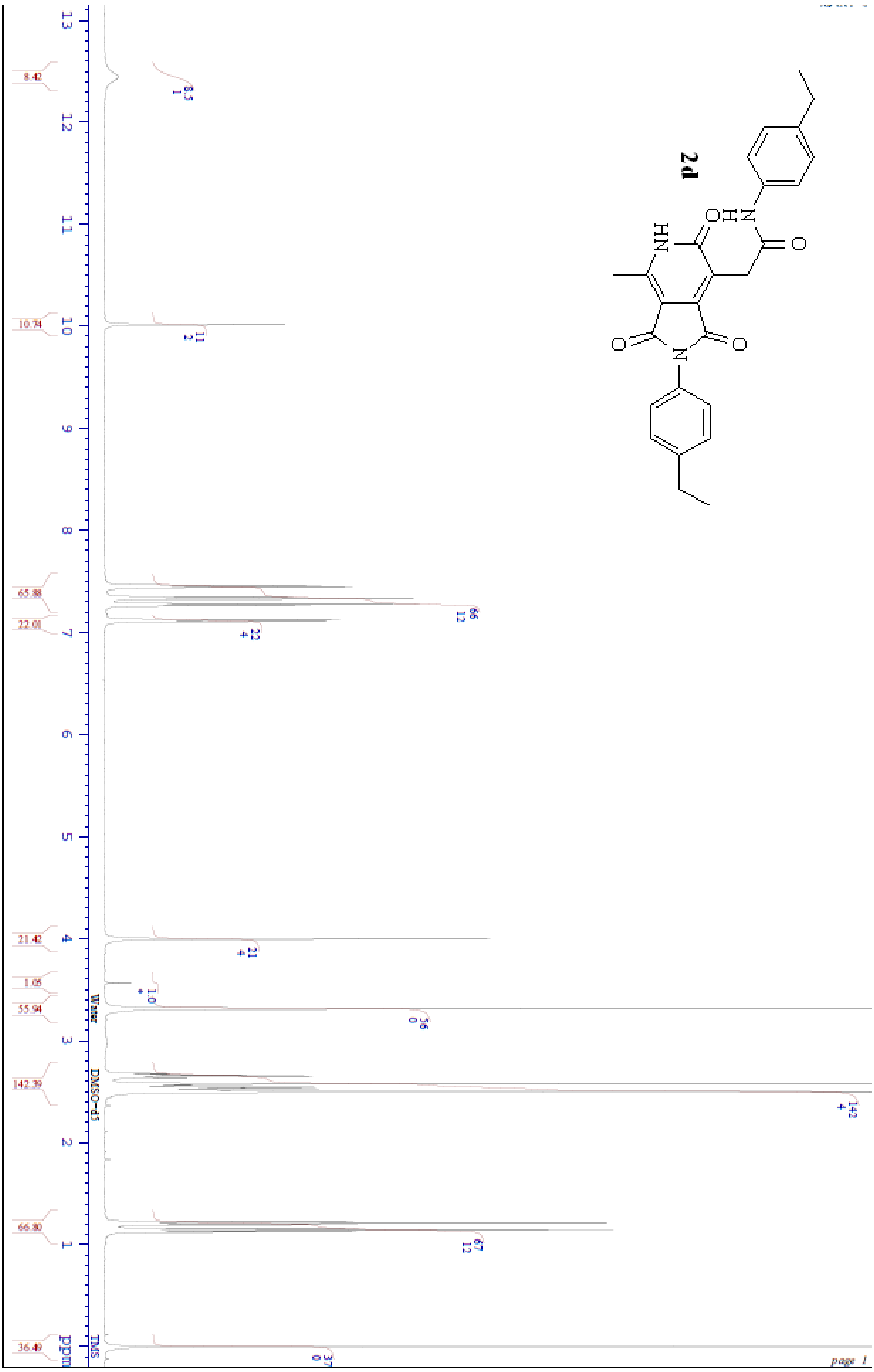

Figure S25: ^{1}H NMR spectrum (500 MHz, DMSO-d6) of compound **2d**

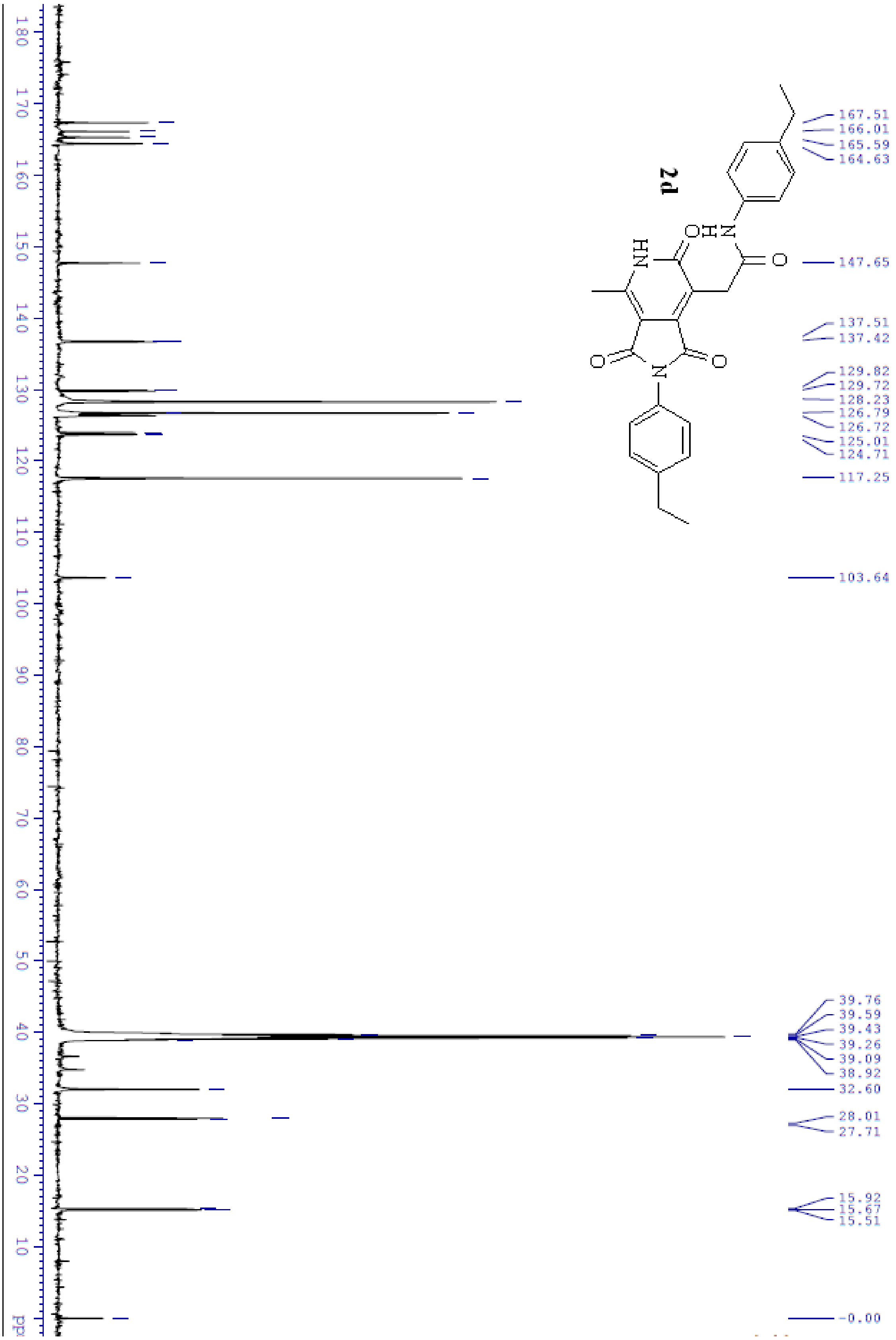

Figure S26: ^{13}C NMR spectrum (125 MHz, DMSO-d6) of compound **2d**

User Chromatograms

Fragmentor Voltage 191 **Collision Energy** 0 **Ionization Mode** ESI

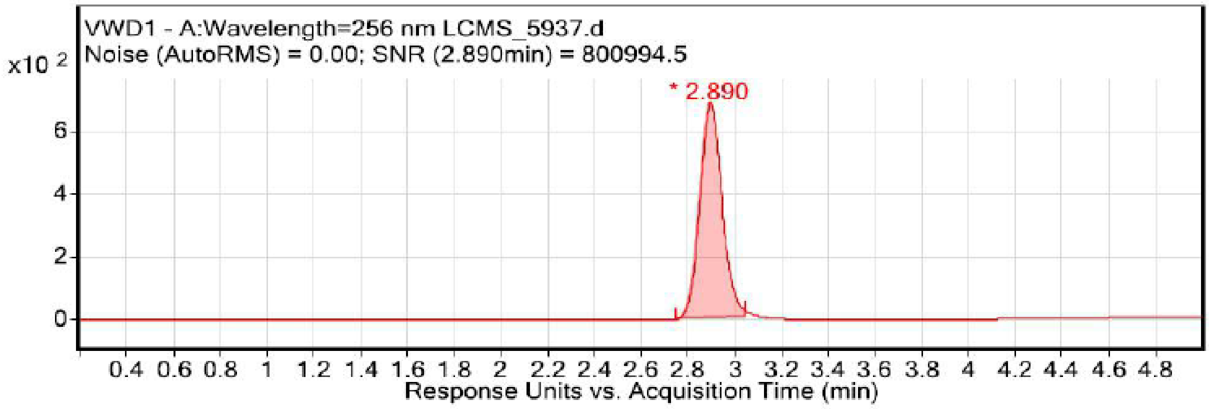

Integration Peak List

Peak	Start	RT	End	Height	Area	Area %	Signal To Noise
1	2.75	2.89	3.043	692.29	4583.64	100	800994.5

Noise Measurements

Noise Type	Signal Definition	Noise Multiplier	Noise Value
Auto-RMS	Height	1	0.000864285

User Spectra

Spectrum Source Peak (1) in "+ TIC Scan Smo" **Fragmentor Voltage** 191 **Collision Energy** 0 **Ionization Mode** ESI

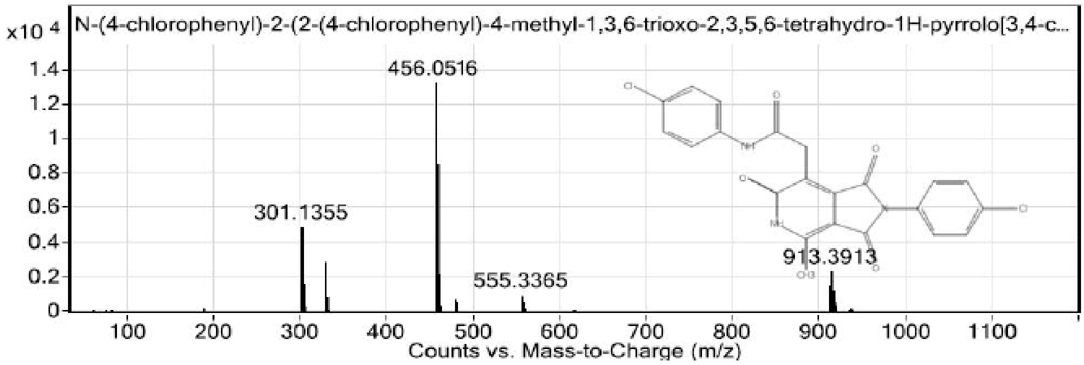

Figure S27: Data HPLC of compound **2e**

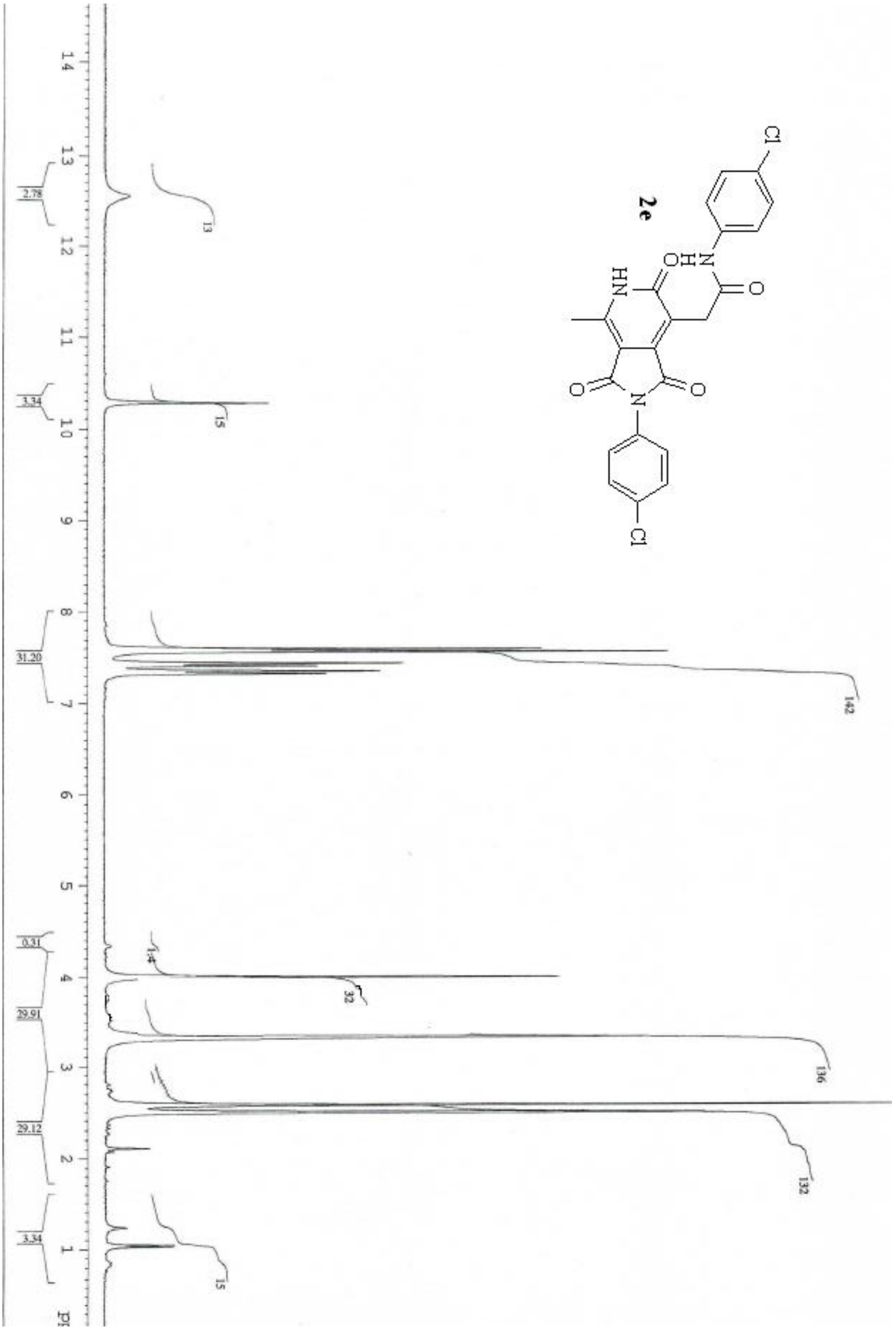

Figure S28: ^{1}H NMR spectrum (500 MHz, DMSO-d6) of compound **2e**

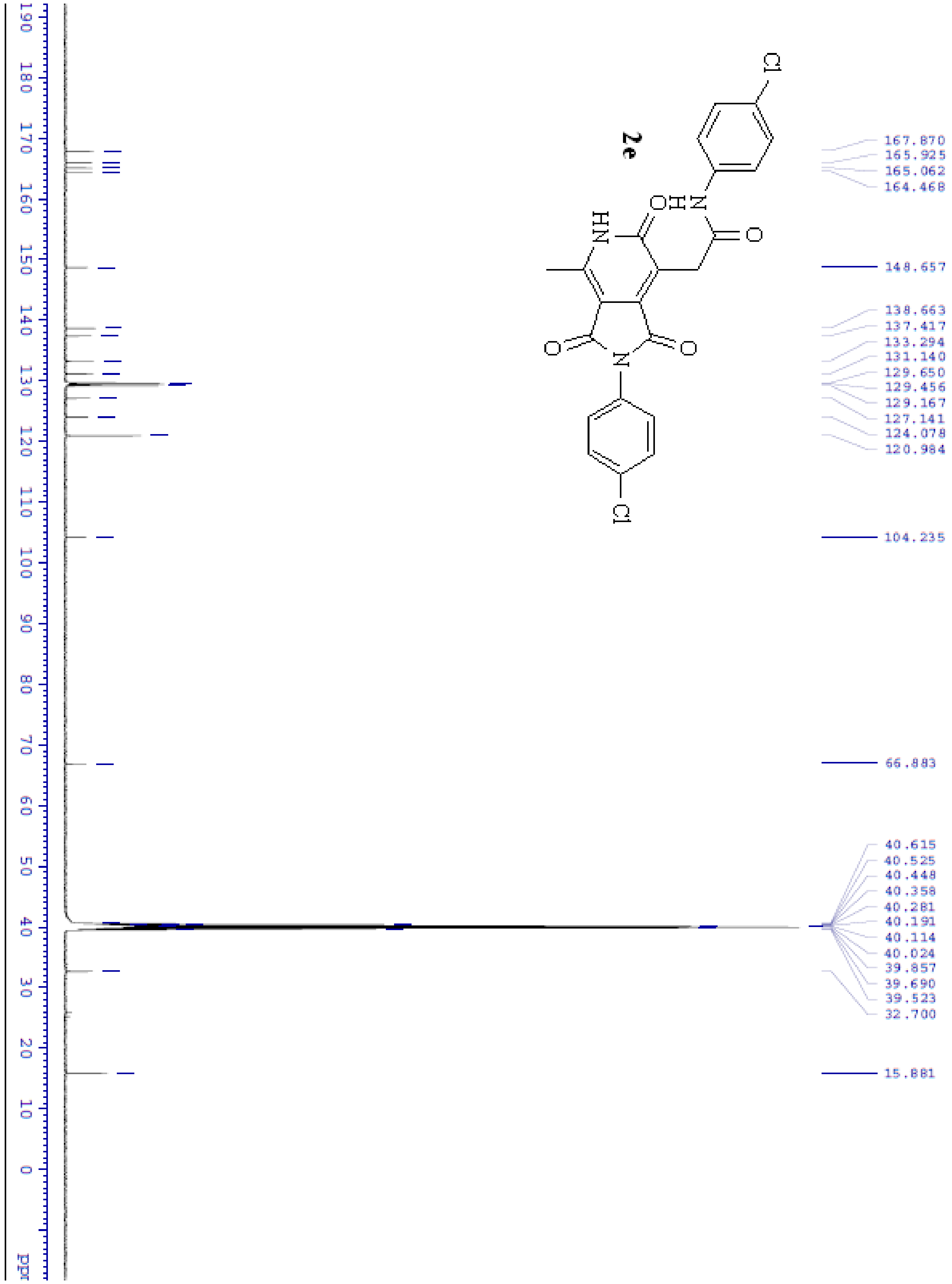

Figure S29: ^{13}C NMR spectrum (125 MHz, DMSO-d6) of compound **2e**

User Chromatograms

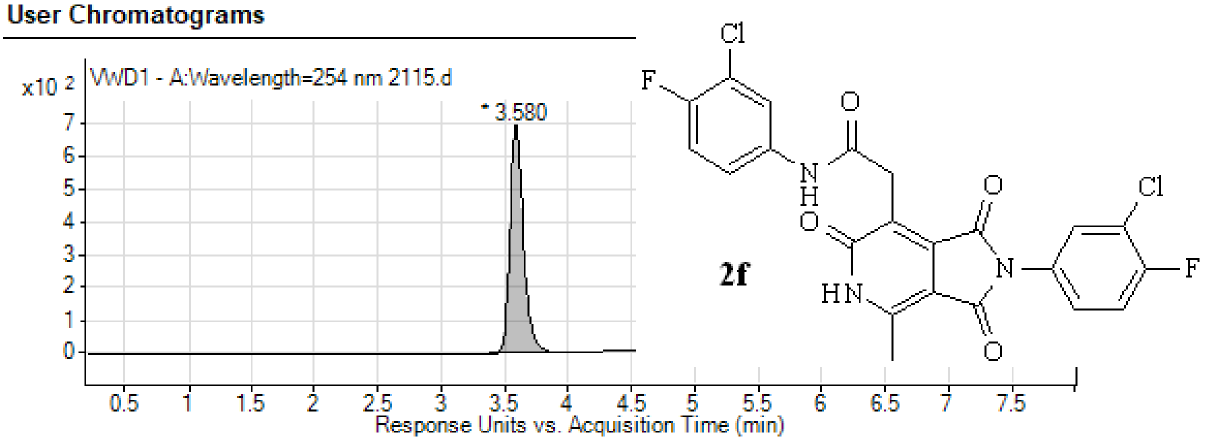

Integration Peak List

Peak	Start	RT	End	Height	Area	Area %
1	3,377	3,58	3,977	696,92	5138,01	100

User Spectra

Fragmentor Voltage	Collision Energy	Ionization Mode
191	0	ESI

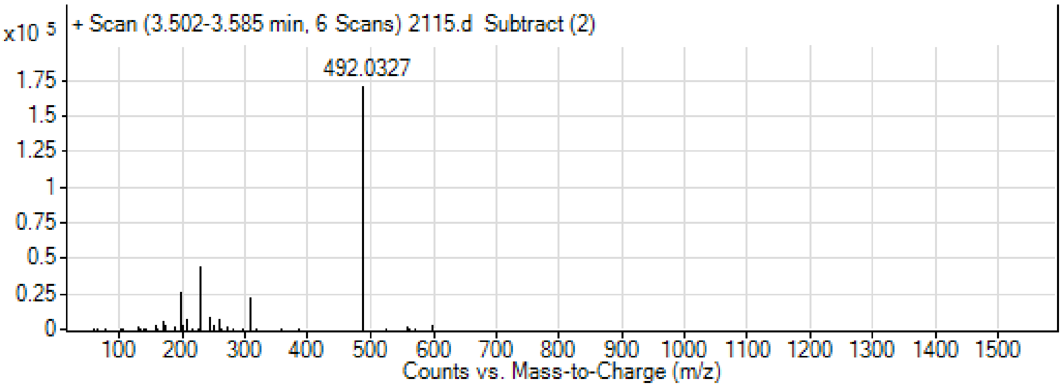

Figure S30: Data HPLC of compound **2f**

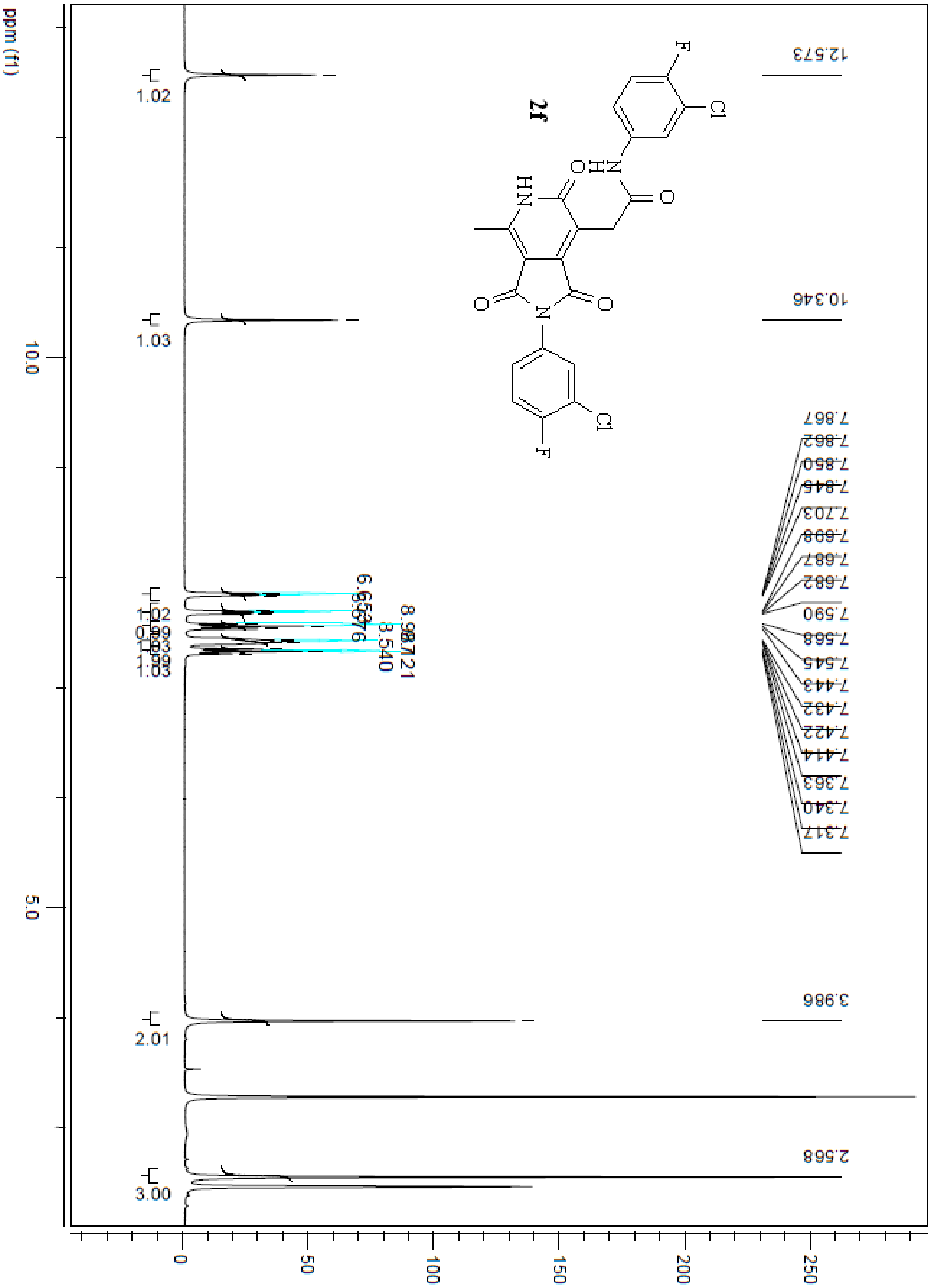

Figure S31: ^{1}H NMR spectrum (500 MHz, DMSO-d6) of compound **2f**

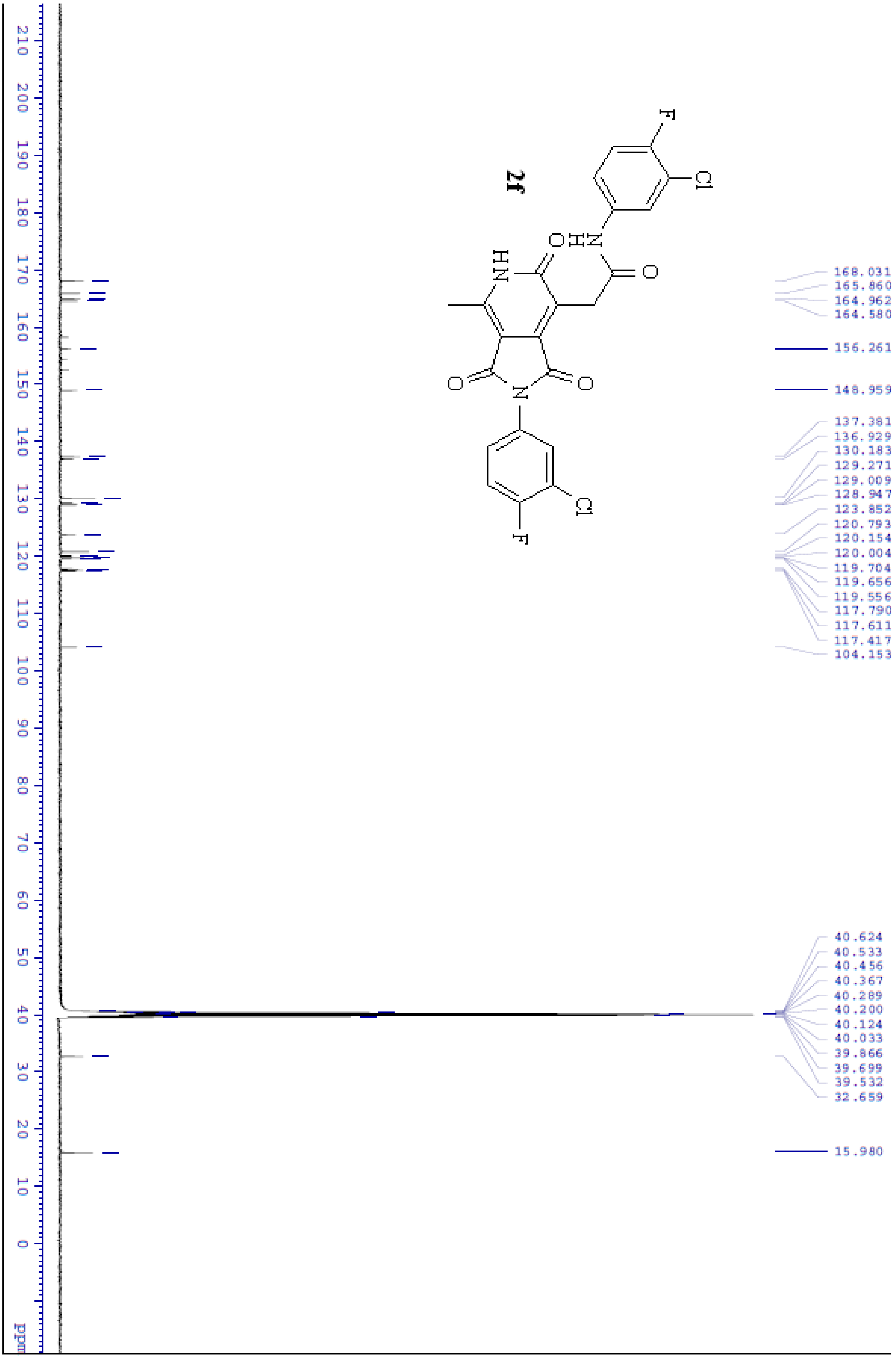

Figure S32: ^{13}C NMR spectrum (125 MHz, DMSO-d6) of compound **2f**

User Chromatograms

Fragmentor Voltage 191 **Collision Energy** 0 **Ionization Mode** ESI

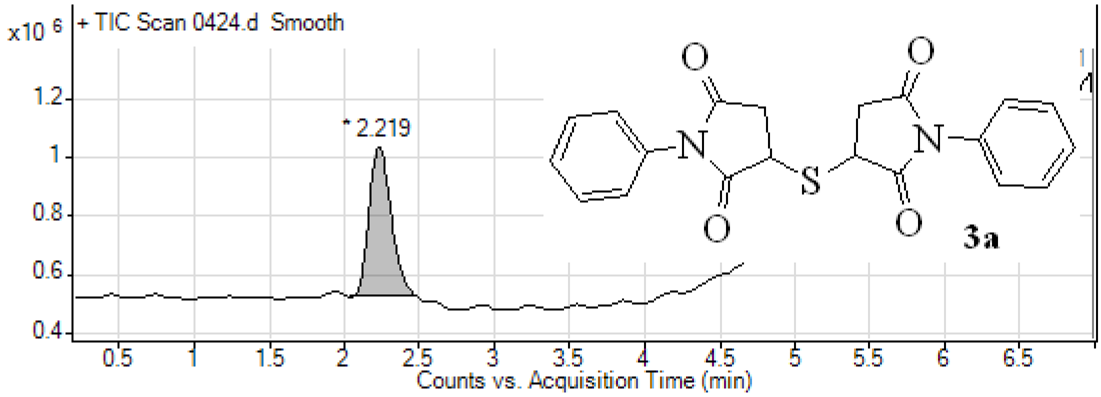

Integration Peak List

Peak	Start	RT	End	Height	Area	Area %
1	2,037	2,219	2,468	511906,16	5209411,17	100

User Spectra

Fragmentor Voltage	Collision Energy	Ionization Mode
191	0	ESI

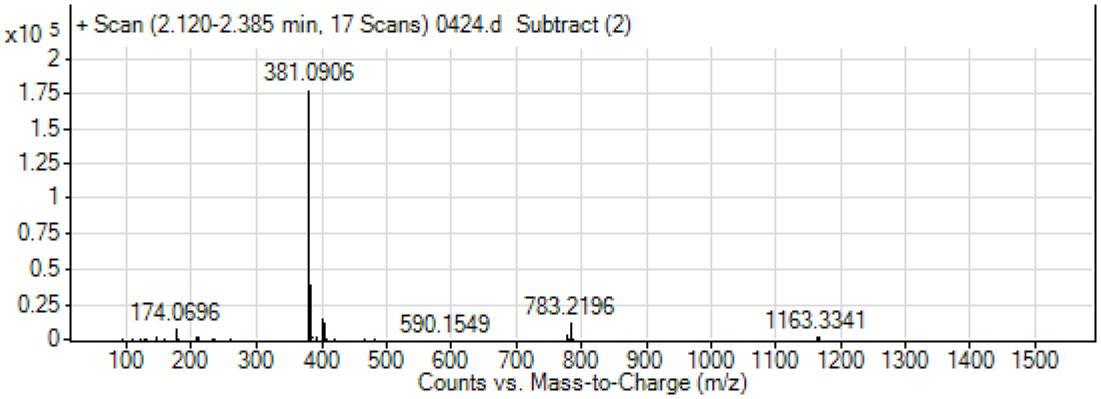

Figure S33: Data HPLC of compound **3a**

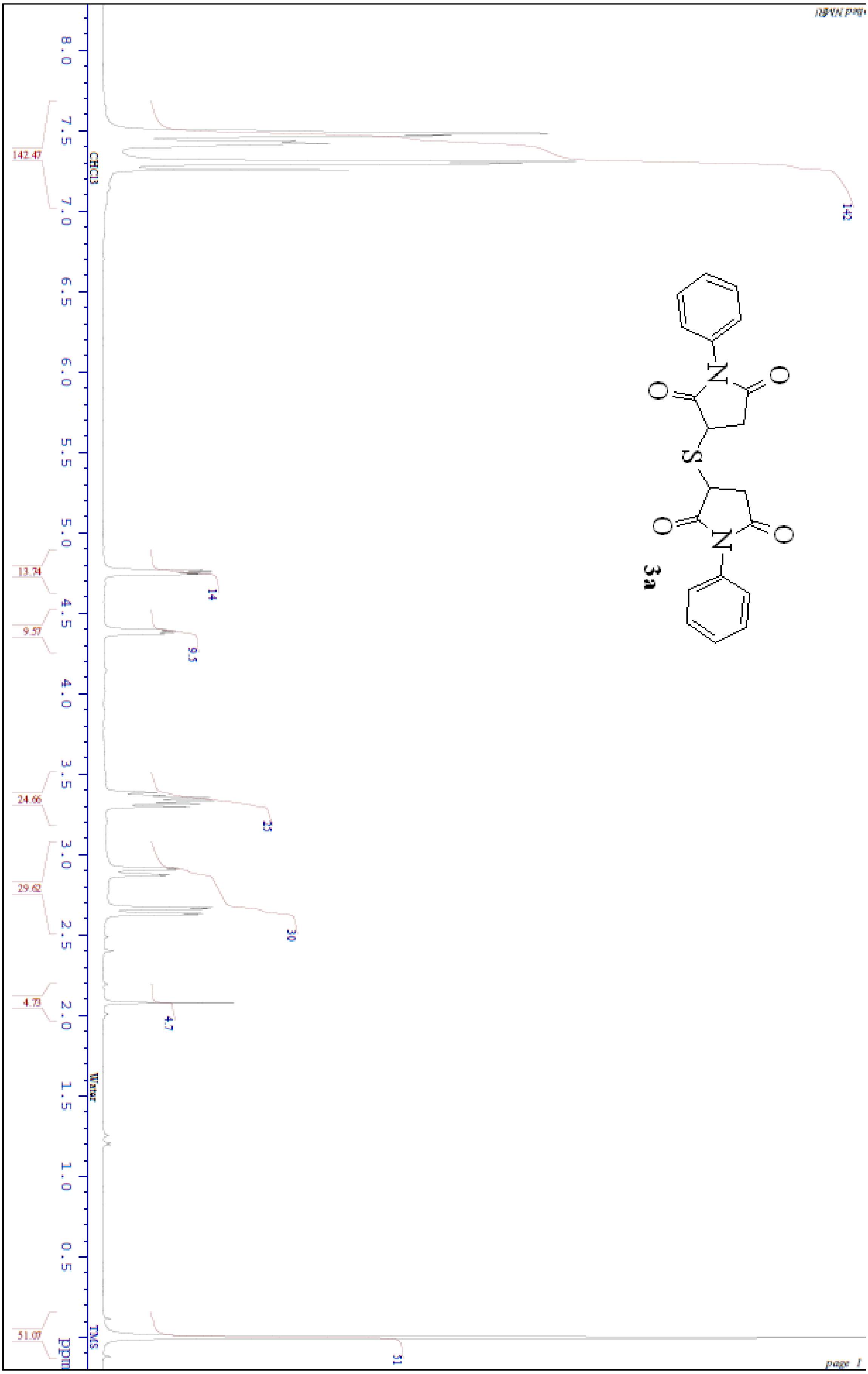

Figure S34: ^{1}H NMR spectrum (500 MHz, $CDCl_3$) of compound **3a**

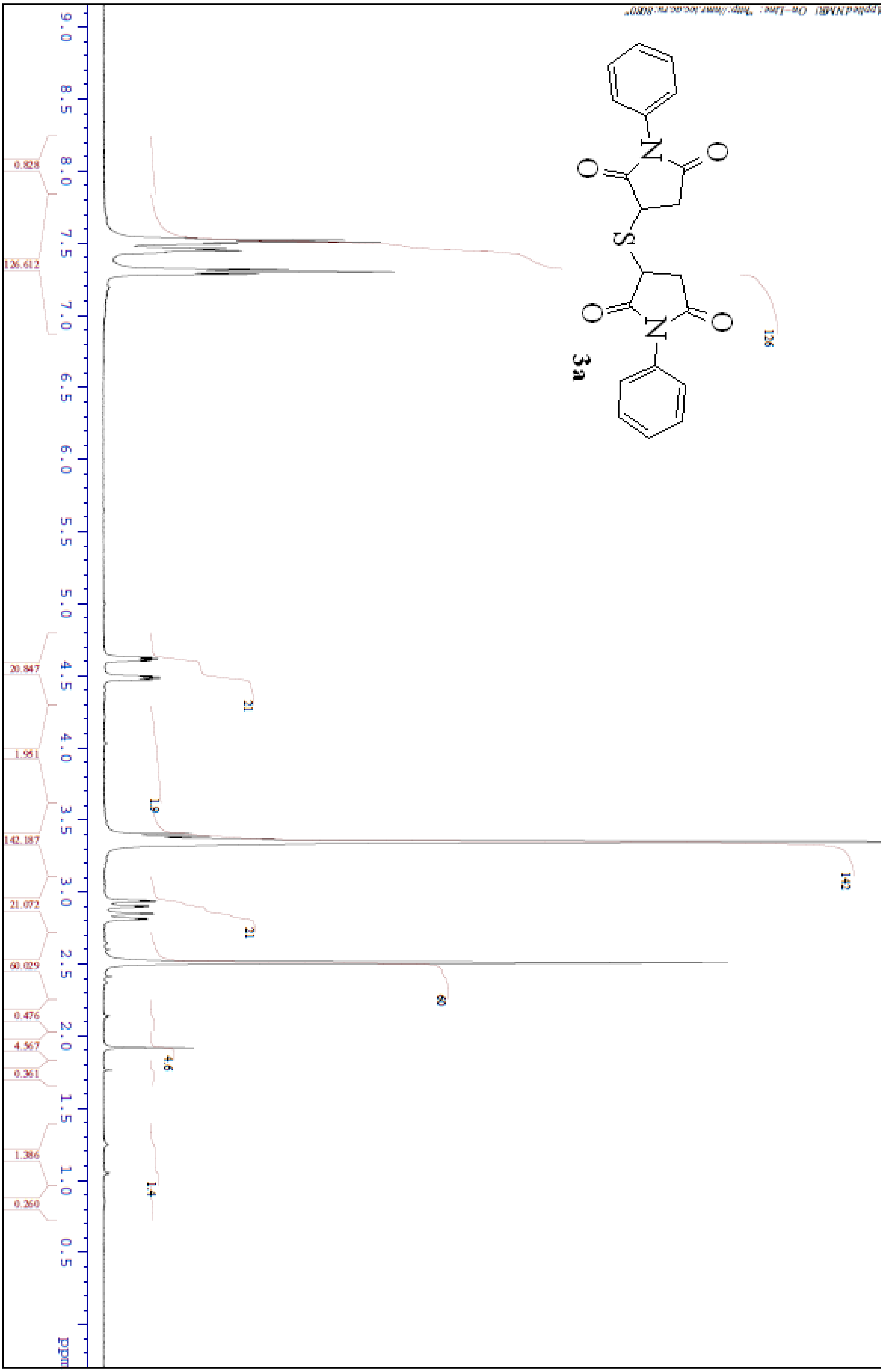

Figure S35: ^{1}H NMR spectrum (500 MHz, DMSO-d6) of compound **3a**

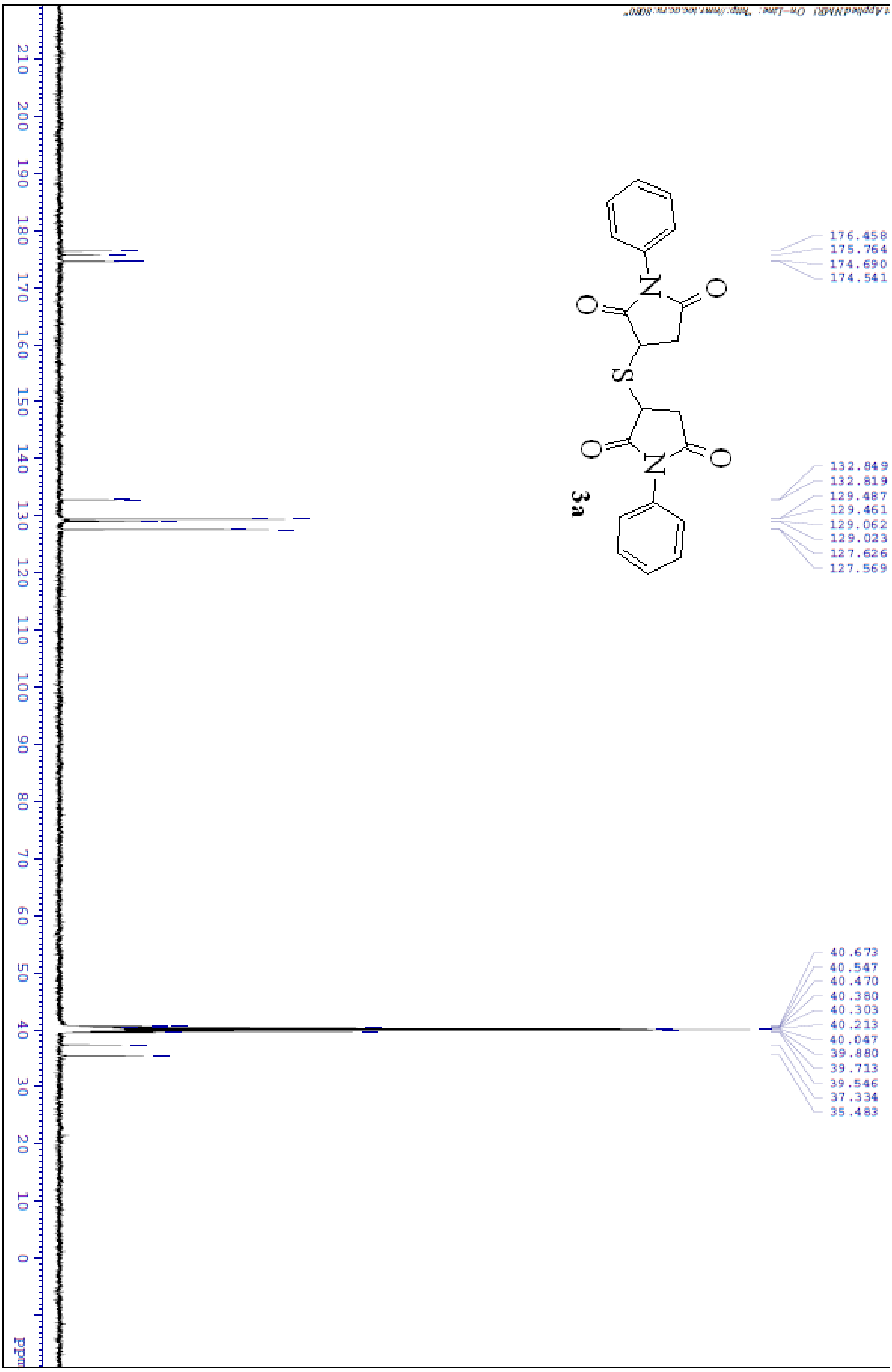

Figure S36: ^{13}C NMR spectrum (125 MHz, DMSO-d6) of compound **3a**

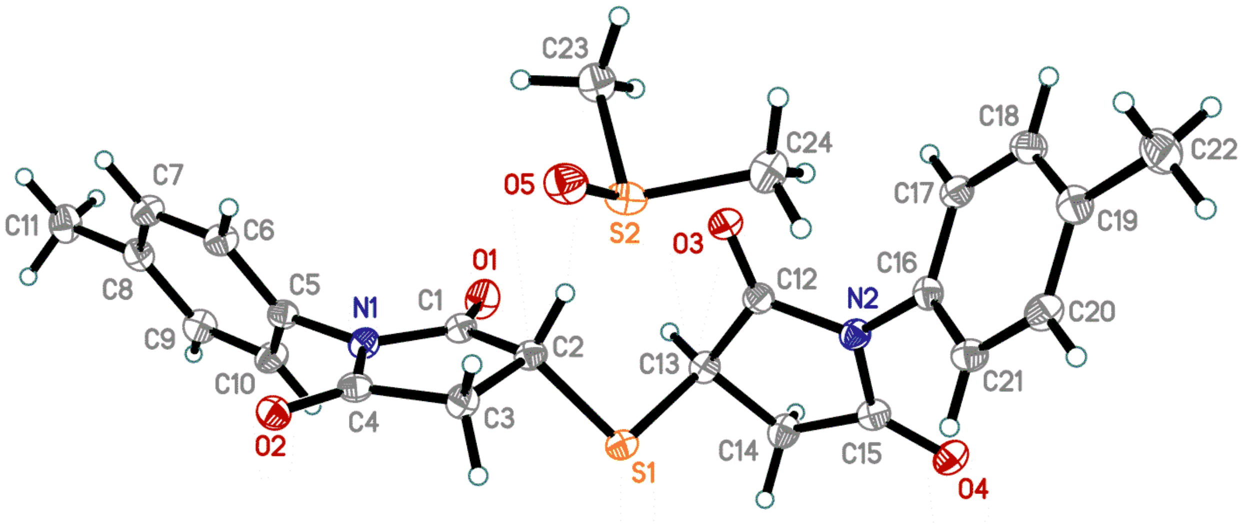

Figure S37: Molecular structure of **3b**•DMSO. A probability level for atomic displacement parameters for non-H atoms is set to 50%.

Table S1. Crystal data and structure refinement for **3b**.

Empirical formula	$C_{22}H_{20}N_2O_4S$•C_2H_6OS
Formula weight	486.59
Temperature, K	100(2)
Wavelength, Å	0.71073
Crystal system	Triclinic
Space group	$P\bar{1}$
Unit cell dimensions	
a, Å	5.6417(2)
b, Å	13.7700(5)
c, Å	15.1459(6)
α, °	92.2380(10)
β, °	95.0400(10)
γ, °	95.8090(10)
Volume, $Å^3$	1164.69(8)
Z	2
Density (cald.), g•cm^{-3}	1.387
Absorption coefficient, mm^{-1}	0.268
F(000)	512
Crystal size, mm	0.53×0.38×0.30
θ range for data collection, °	2.058-34.981
Index ranges	-9<=h<=9,
	-22<=k<=22,
	-24<=l<=24
Reflections	
Collected	60803
Independent [R_{int}]	10230 [0.1044]
Observed (with I>2σ(I))	6257

Completeness to θ_{max} / θ_{full}	0.999 / 0.999
Data / restraints / parameters	10230 / 0 / 302
Goodness-of-fit on F^2	1.026
R1 / wR2 indices for I>2σ(I)	0.0683 / 0.1208
R1 / wR2 indices (all data)	0.1329 / 0.1463
$\Delta\rho(\bar{e})_{max}$ / $\Delta\rho(\bar{e})_{min}$, $\bar{e}$•$Å^{-3}$	0.501 / -0.674

Table S2. Selected bond lengths for **3b**, Å.

S1-C2	1.8163(19)	N2-C16	1.432(2)	C12-C13	1.517(3)
S1-C13	1.827(2)	C1-C2	1.513(3)	C13-C14	1.535(3)
O1-C1	1.211(2)	C2-C3	1.529(3)	C14-C15	1.509(3)
O2-C4	1.208(2)	C3-C4	1.506(3)	C16-C21	1.387(3)
O3-C12	1.213(2)	C5-C6	1.385(3)	C16-C17	1.388(3)
O4-C15	1.204(2)	C5-C10	1.386(3)	C17-C18	1.383(3)
N1-C1	1.388(3)	C6-C7	1.391(3)	C18-C19	1.396(3)
N1-C4	1.401(2)	C7-C8	1.392(3)	C19-C20	1.388(3)
N1-C5	1.434(2)	C8-C9	1.393(3)	C19-C22	1.509(3)
N2-C12	1.393(2)	C8-C11	1.506(3)	C20-C21	1.389(3)
N2-C15	1.400(2)	C9-C10	1.390(3)		

Table S3. Selected angle values for **3b**, °.

C2-S1-C13	102.28(9)	N1-C4-C3	107.99(16)	C14-C13-S1	108.24(14)
C1-N1-C4	112.89(16)	C6-C5-C10	121.11(18)	C15-C14-C13	105.07(16)
C1-N1-C5	122.68(16)	C6-C5-N1	119.04(17)	O4-C15-N2	124.08(19)
C4-N1-C5	124.38(16)	C10-C5-N1	119.81(17)	O4-C15-C14	127.90(18)
C12-N2-C15	112.73(16)	C5-C6-C7	119.26(18)	N2-C15-C14	108.00(16)
C12-N2-C16	122.94(16)	C6-C7-C8	120.97(19)	C21-C16-C17	120.76(18)
C15-N2-C16	124.29(16)	C7-C8-C9	118.42(18)	C21-C16-N2	119.50(17)
O1-C1-N1	124.60(18)	C7-C8-C11	120.96(19)	C17-C16-N2	119.71(17)
O1-C1-C2	127.08(18)	C9-C8-C11	120.62(19)	C18-C17-C16	119.08(18)
N1-C1-C2	108.32(16)	C10-C9-C8	121.48(18)	C17-C18-C19	121.39(19)
C1-C2-C3	104.92(16)	C5-C10-C9	118.76(18)	C20-C19-C18	118.35(19)
C1-C2-S1	111.76(13)	O3-C12-N2	124.46(18)	C20-C19-C22	121.55(19)
C3-C2-S1	110.58(13)	O3-C12-C13	127.33(18)	C18-C19-C22	120.10(19)
C4-C3-C2	105.53(15)	N2-C12-C13	108.16(16)	C19-C20-C21	121.13(19)
O2-C4-N1	124.27(18)	C12-C13-C14	104.26(16)	C16-C21-C20	119.28(18)
O2-C4-C3	127.71(17)	C12-C13-S1	107.67(13)		

User Chromatograms

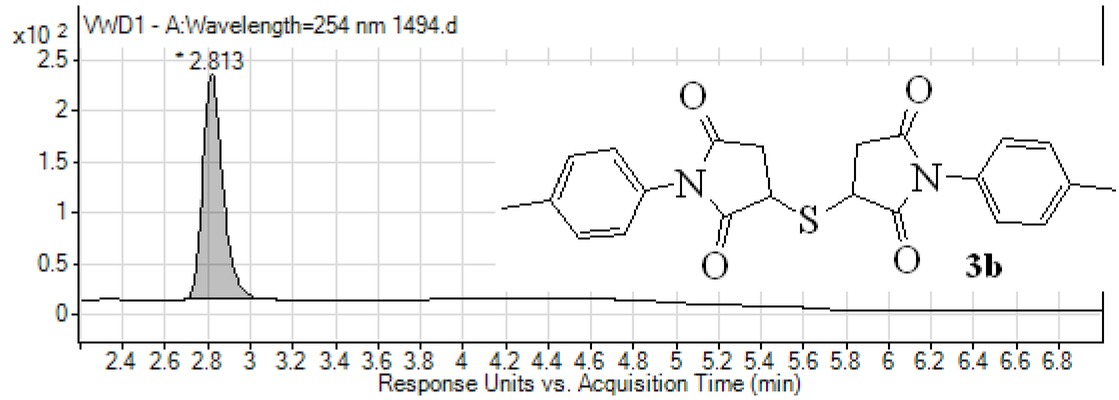

Integration Peak List

Peak	Start	RT	End	Height	Area	Area %
1	2,69	2,813	3,073	221,81	1456,45	100

User Spectra

Fragmentor Voltage	Collision Energy	Ionization Mode
191	0	ESI

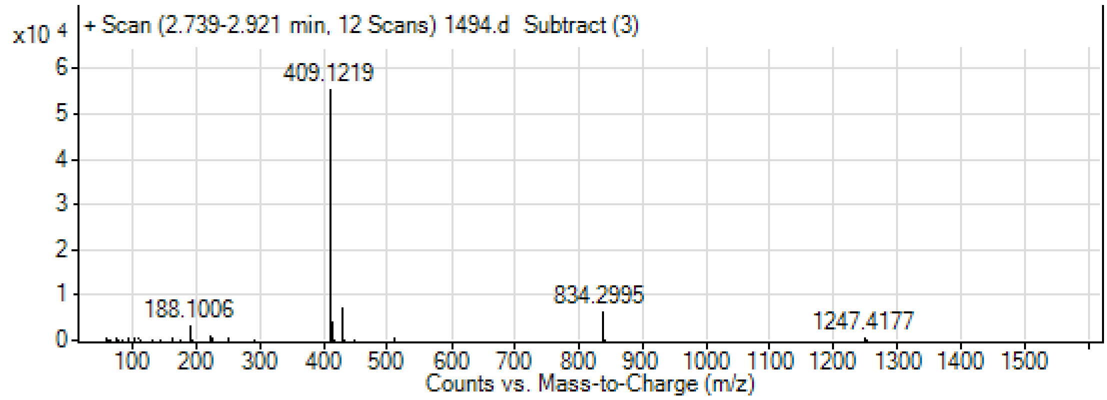

Figure S38: Data HPLC of compound **3b**

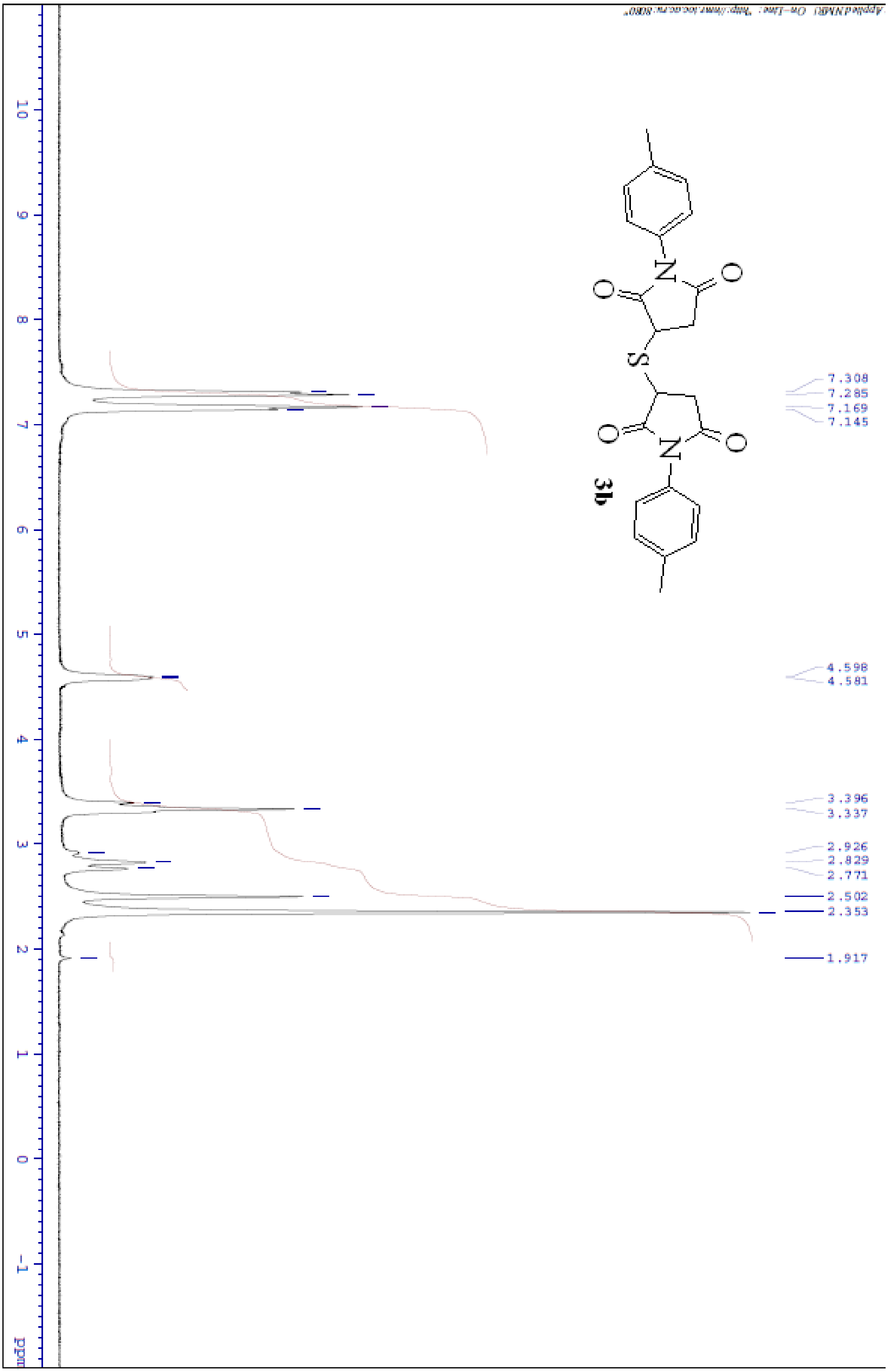

Figure S39: ^{1}H NMR spectrum (500 MHz, DMSO-d6) of compound **3b**

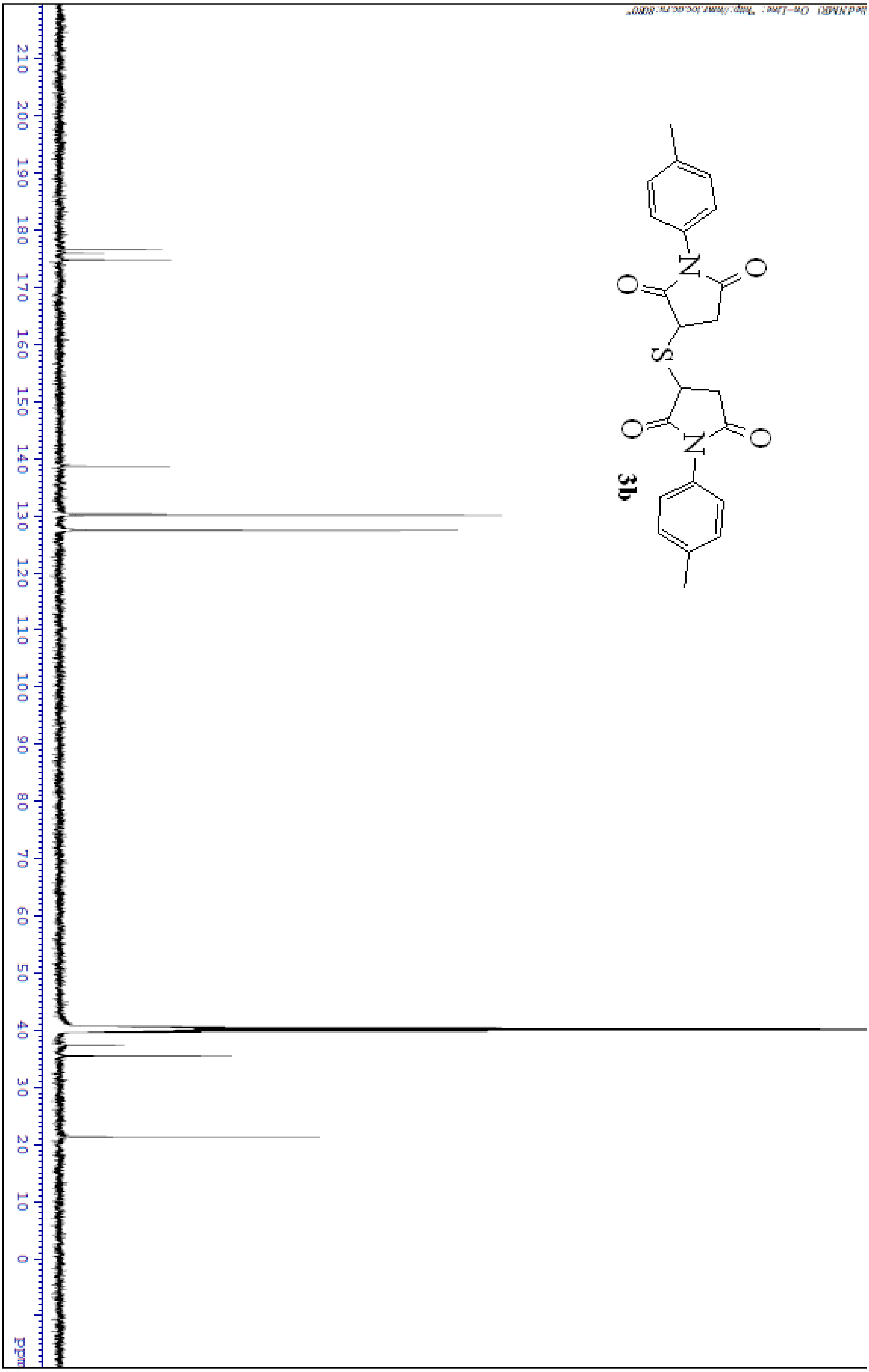

Figure S40: ^{13}C NMR spectrum (125 MHz, DMSO-d6) of compound **3b**

User Chromatograms

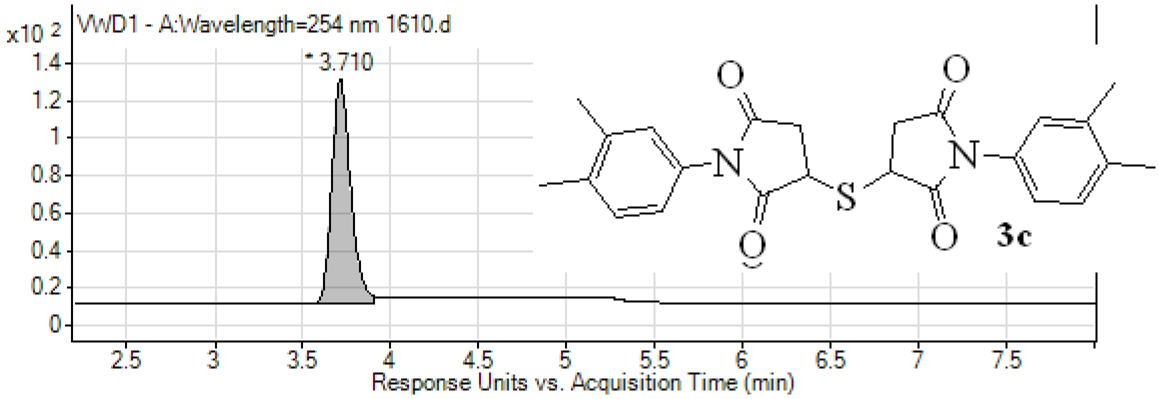

Integration Peak List

Peak	Start	RT	End	Height	Area	Area %
1	3,577	3,71	3,903	119,74	895,67	100

User Spectra

Fragmentor Voltage	Collision Energy	Ionization Mode
191	0	ESI

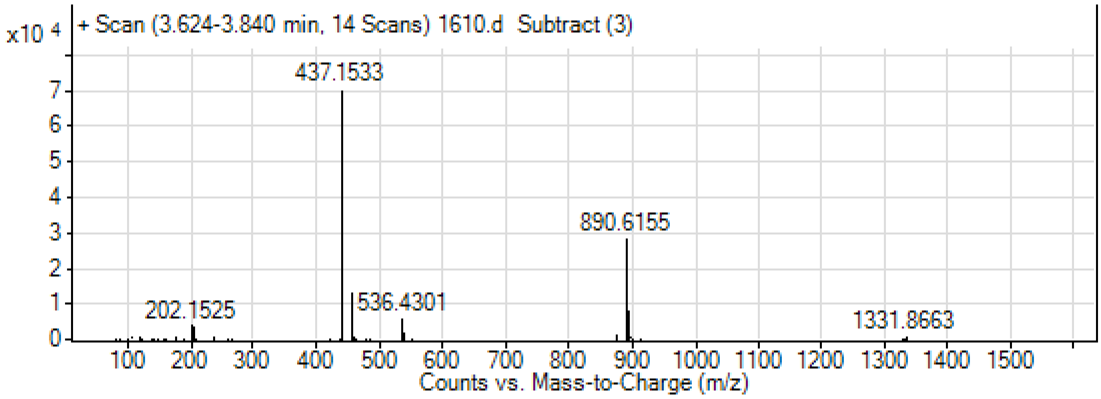

Figure S41: Data HPLC of compound **3c**

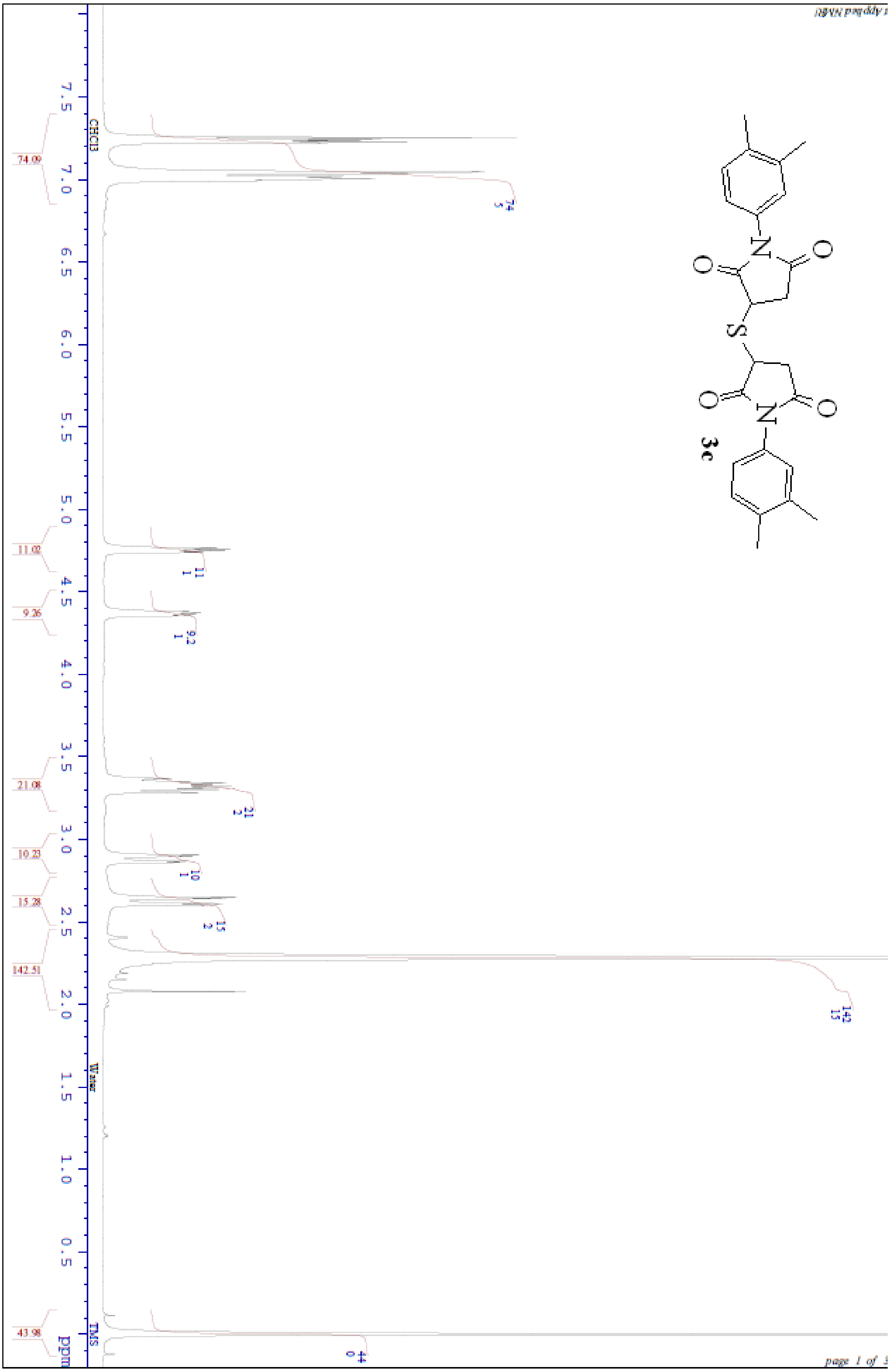

Figure S42: ^{1}H NMR spectrum (500 MHz, $CDCl_3$) of compound **3c**

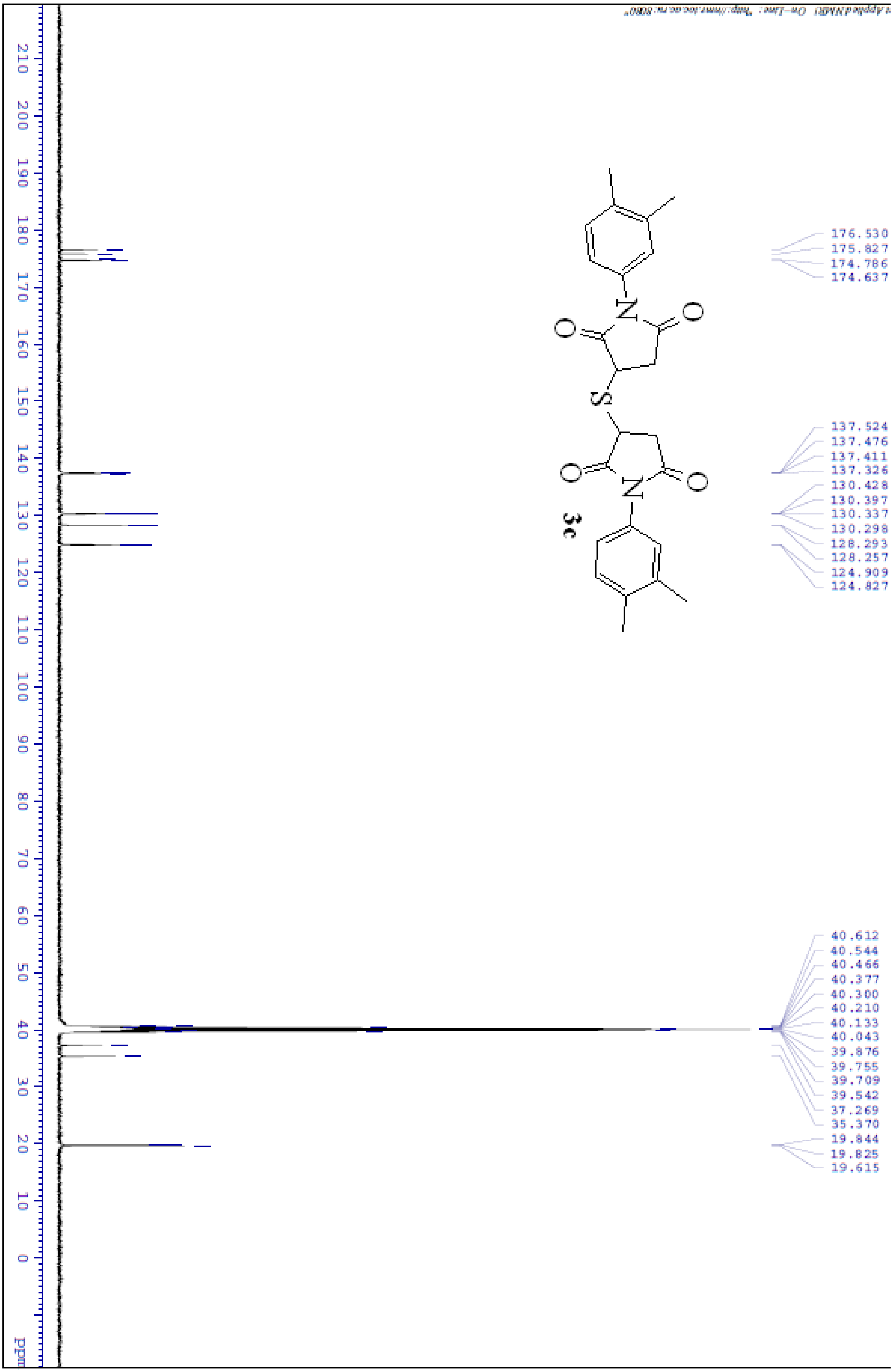

Figure S43: ^{13}C NMR spectrum (125 MHz, DMSO-d6) of compound **3c**

User Chromatograms

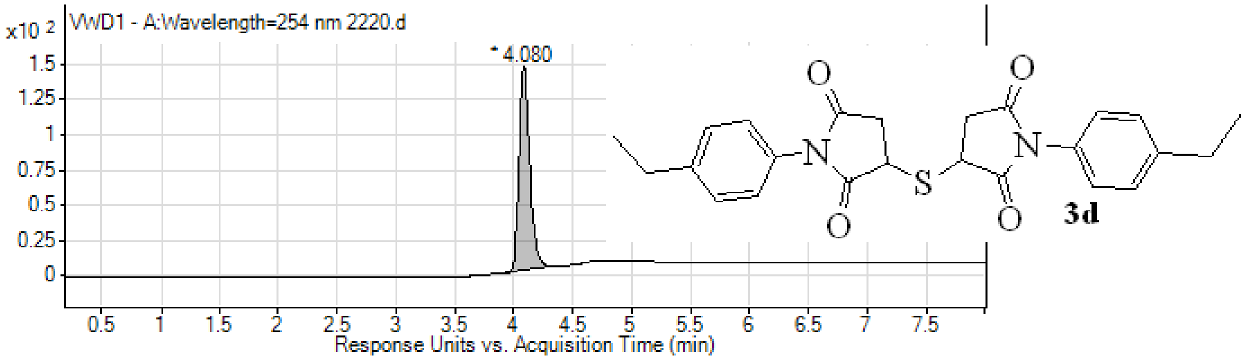

Integration Peak List

Peak	Start	RT	End	Height	Area	Area %
1	3,91	4,08	4,337	144,21	918,31	100

User Spectra

Fragmentor Voltage	Collision Energy	Ionization Mode
191	0	ESI

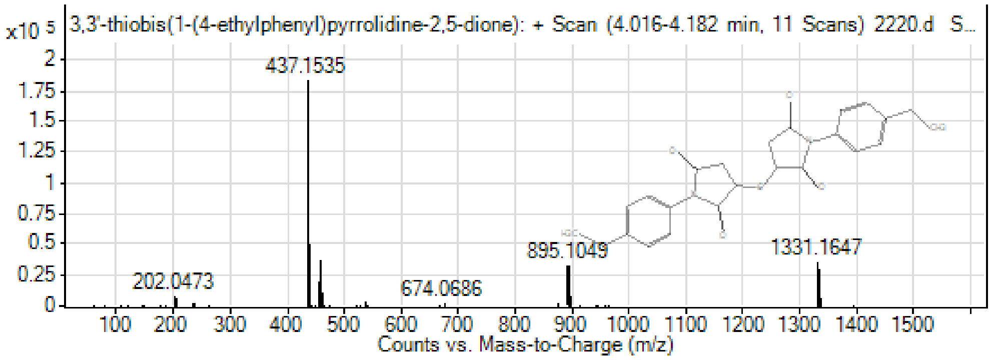

Figure S44: Data HPLC of compound **3d**

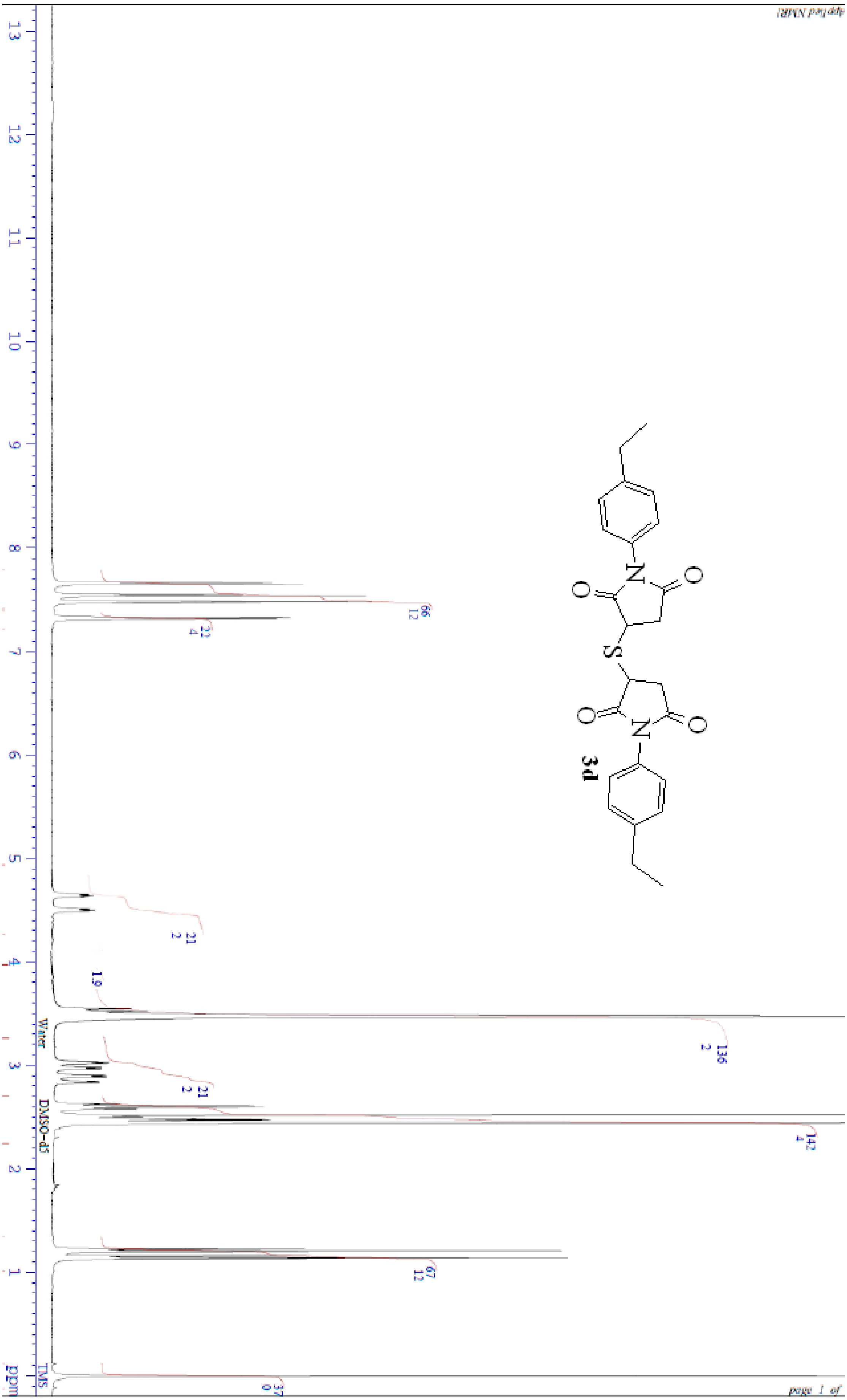

Figure S45: ^{1}H NMR spectrum (500 MHz, DMSO-d6) of compound **3d**

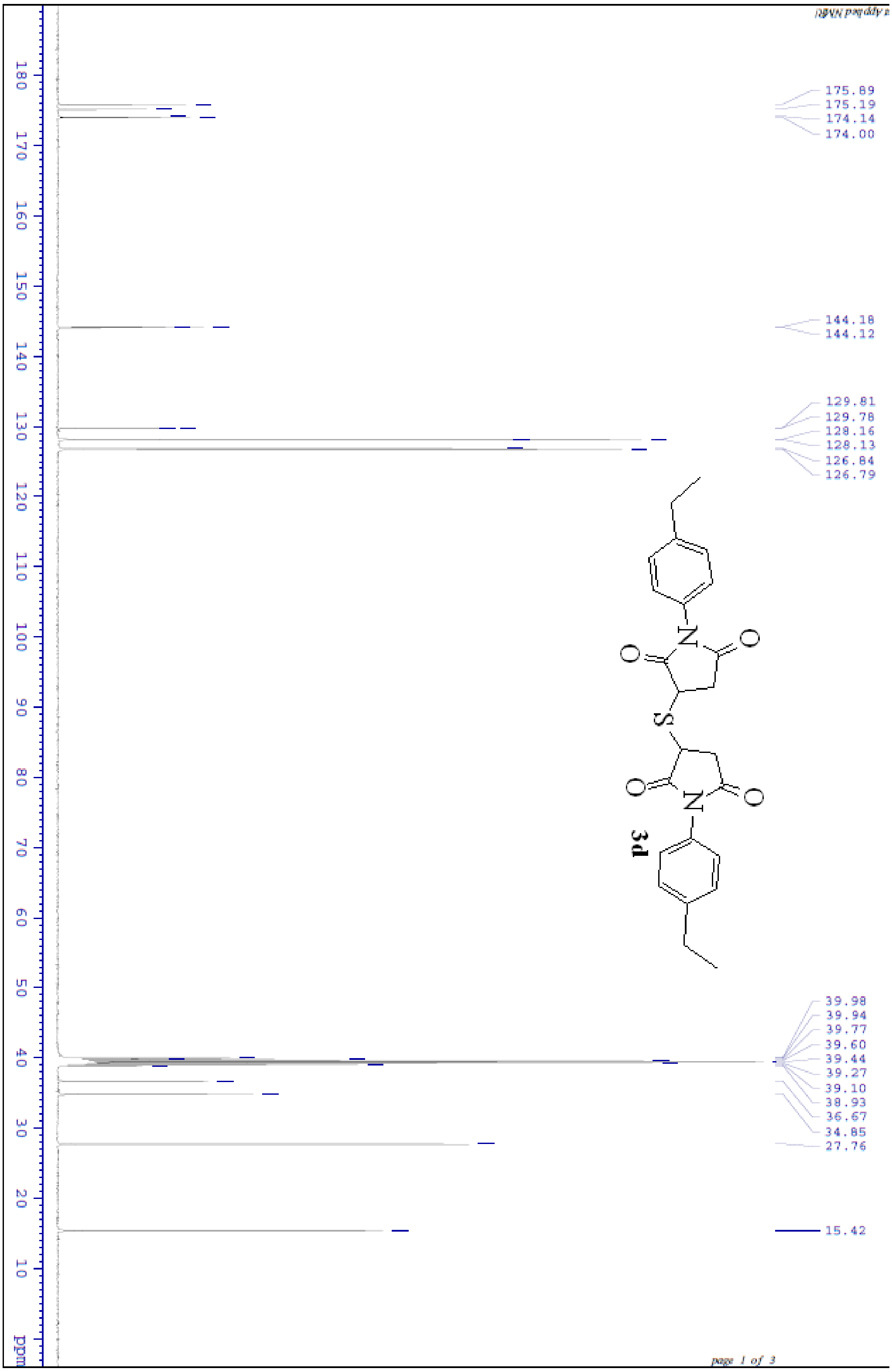

Figure S46: ^{13}C NMR spectrum (125 MHz, DMSO-d6) of compound **3d**

User Chromatograms

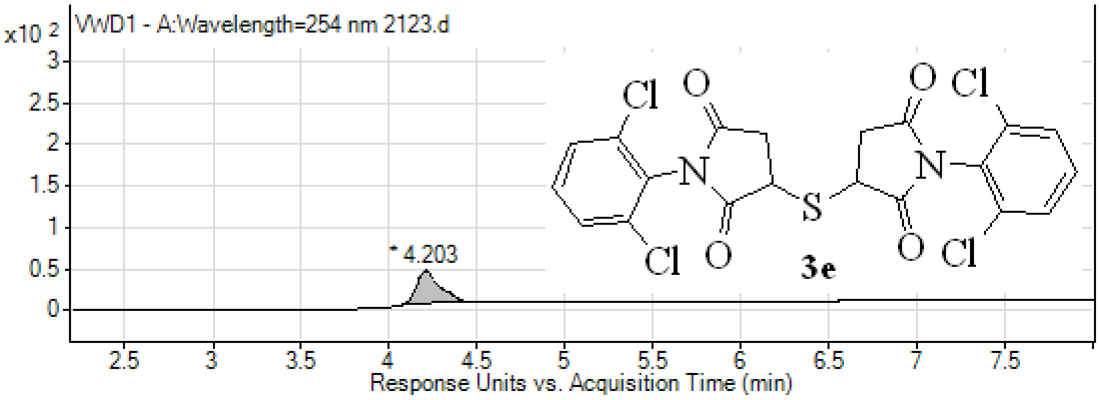

Integration Peak List

Peak	Start	RT	End	Height	Area	Area %
1	4,057	4,203	4,427	40,72	369,79	100

User Spectra

Fragmentor Voltage	Collision Energy	Ionization Mode
191	0	ESI

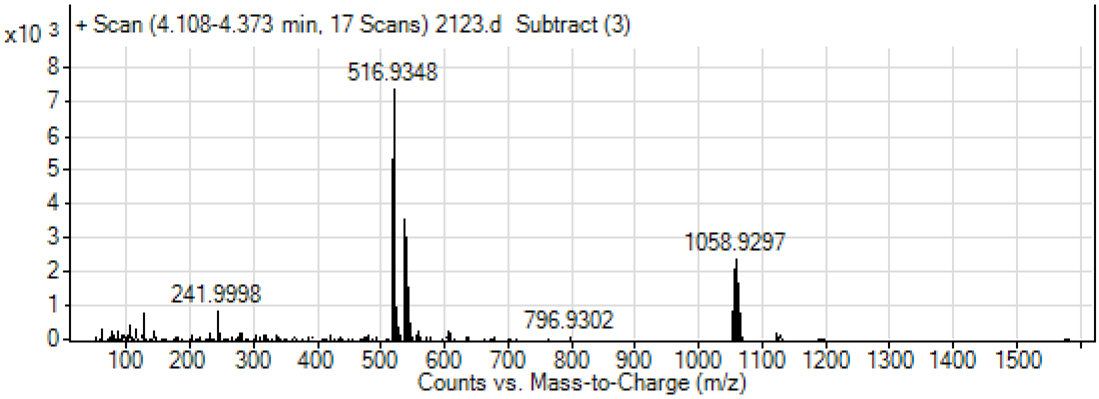

Figure S47: Data HPLC of compound **3e**

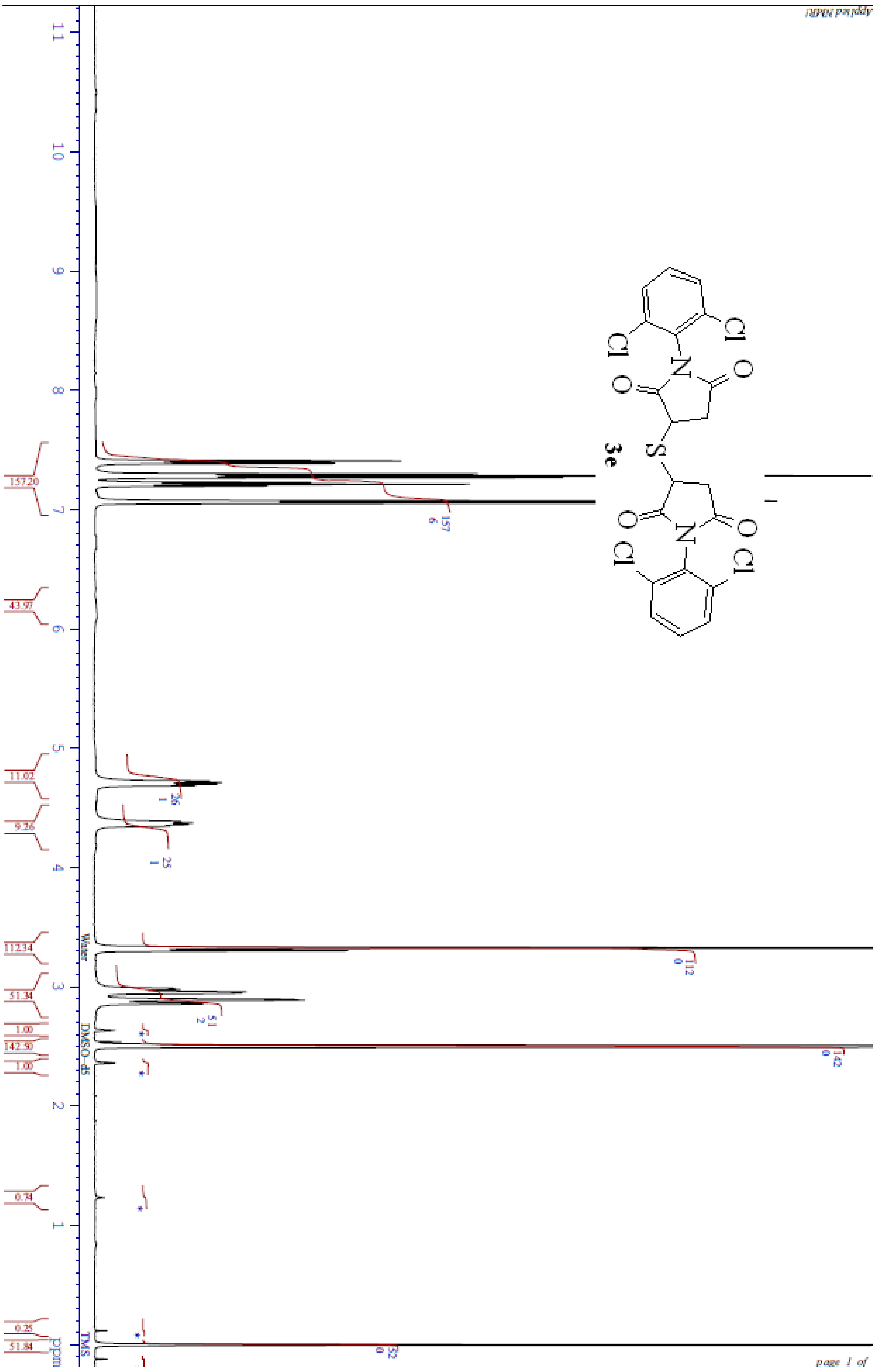

Figure S48: ^{1}H NMR spectrum (500 MHz, DMSO-d6) of compound **3e**

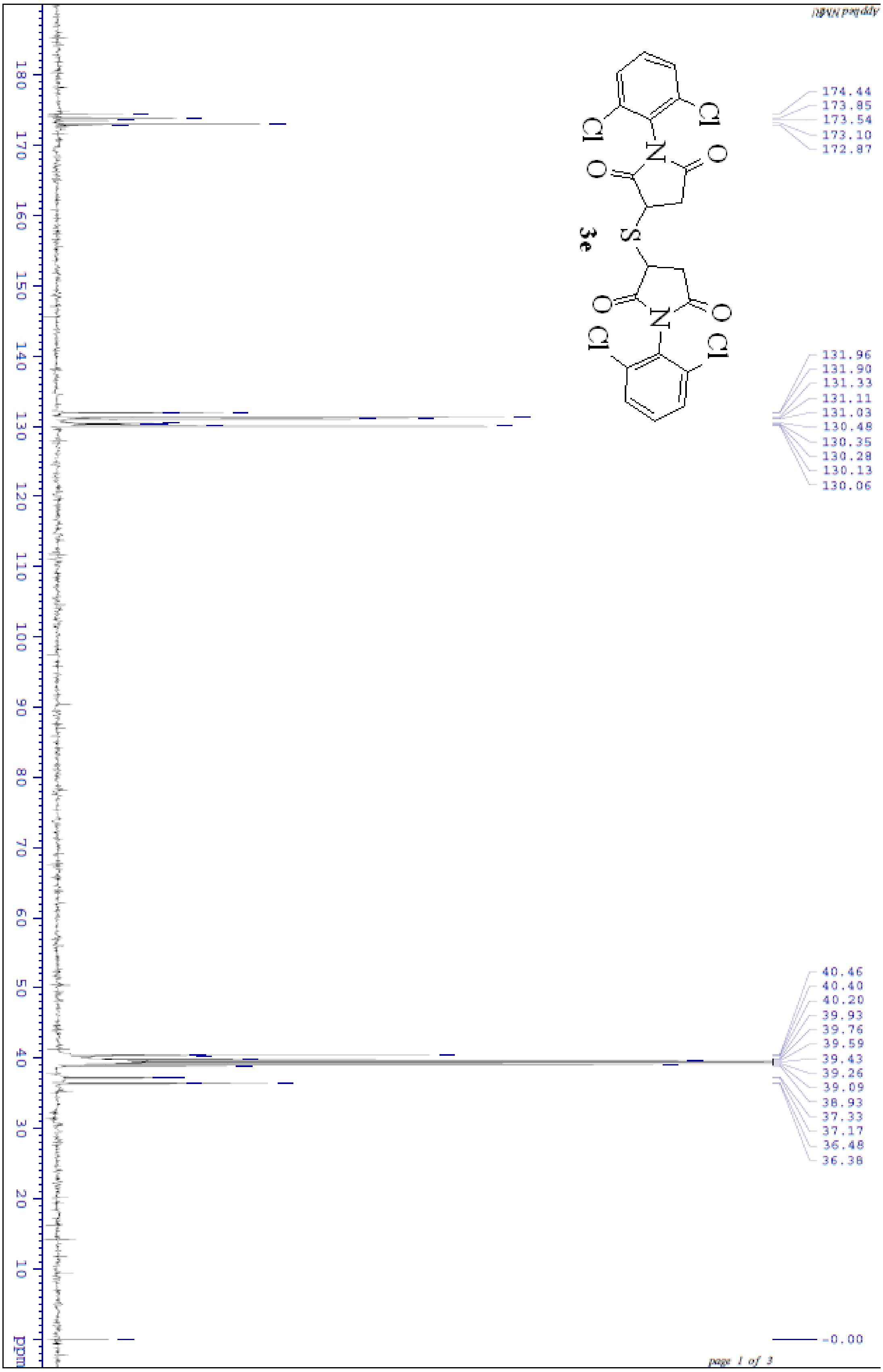

Figure S49: ^{13}C NMR spectrum (125 MHz, DMSO-d6) of compound **3e**

User Chromatograms

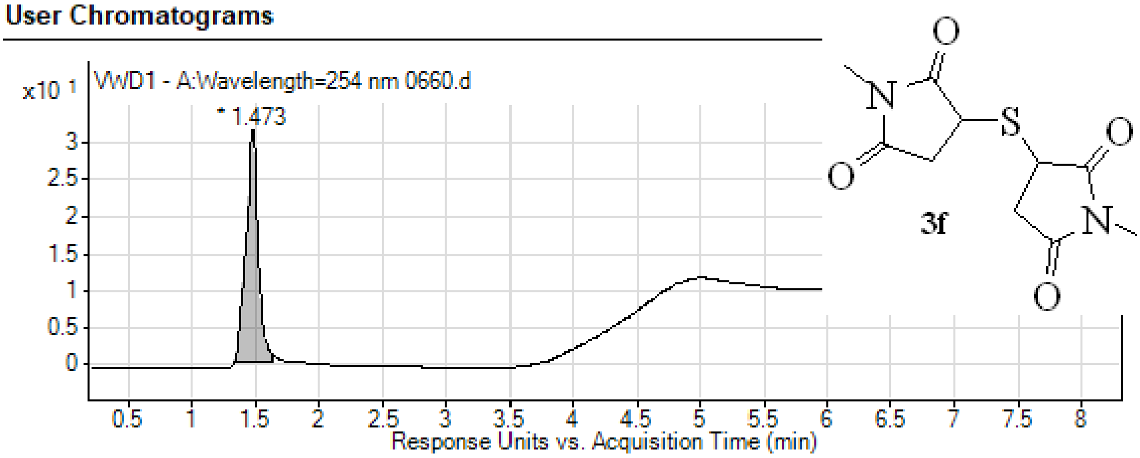

Integration Peak List

Peak	Start	RT	End	Height	Area	Area %
1	1,32	1,473	1,633	31,34	213,37	100

User Spectra

Fragmentor Voltage	Collision Energy	Ionization Mode
185	0	ESI

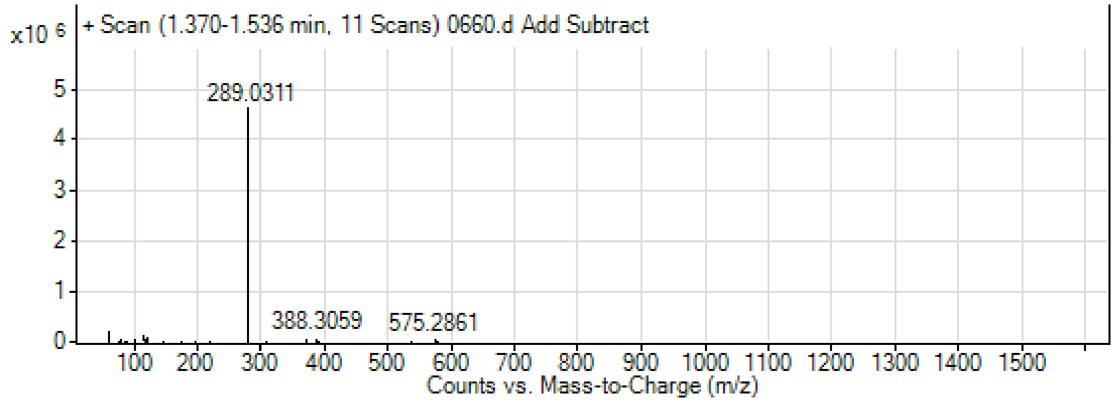

Figure S50: Data HPLC of compound **3f**

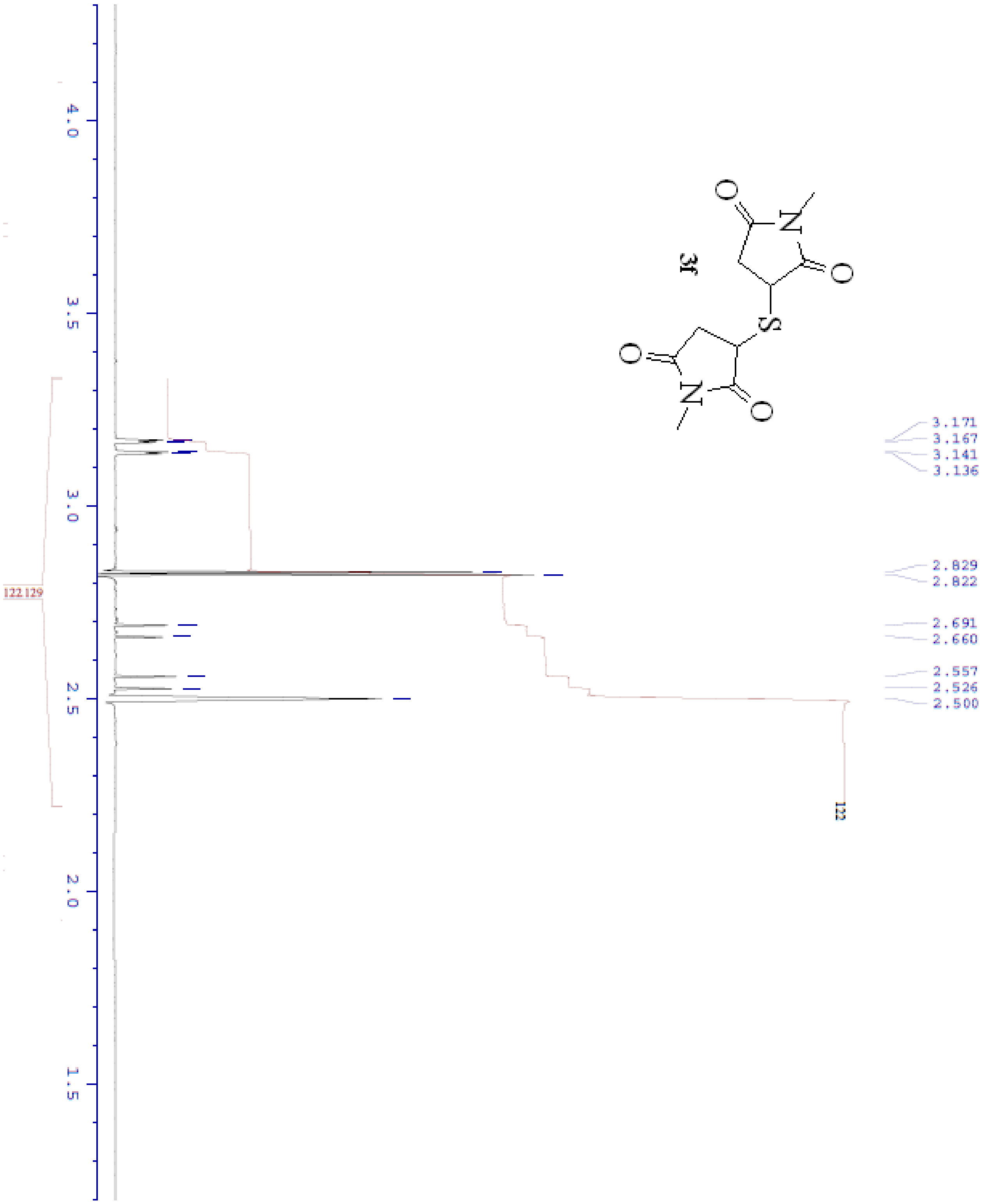

Figure S51: ^{1}H NMR spectrum (500 MHz, DMSO-d6) of compound **3f**

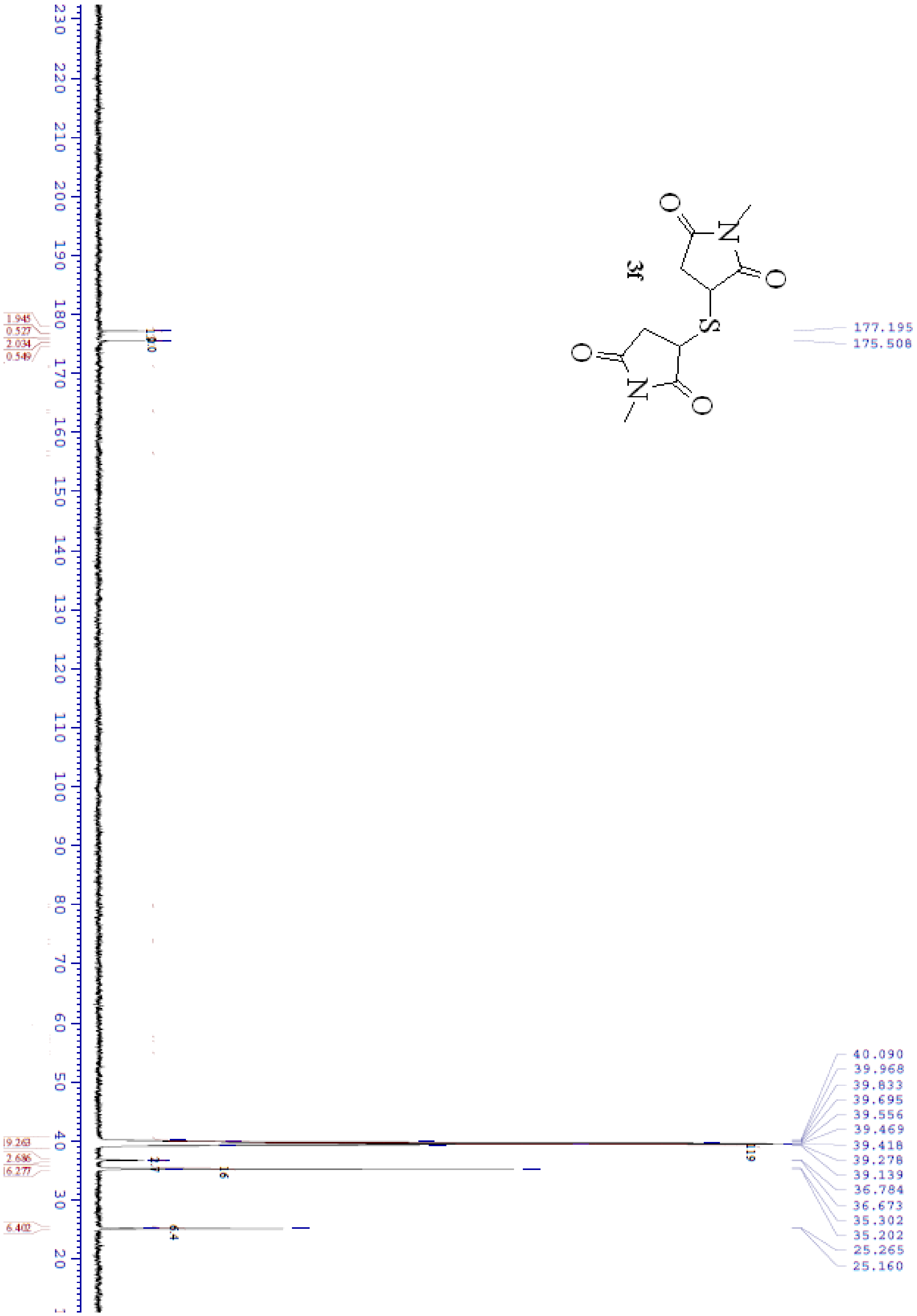

Figure S52: ^{13}C NMR spectrum (125 MHz, DMSO-d6) of compound **3f**

User Chromatograms

Fragmentor Voltage 185 **Collision Energy** 0 **Ionization Mode** ESI

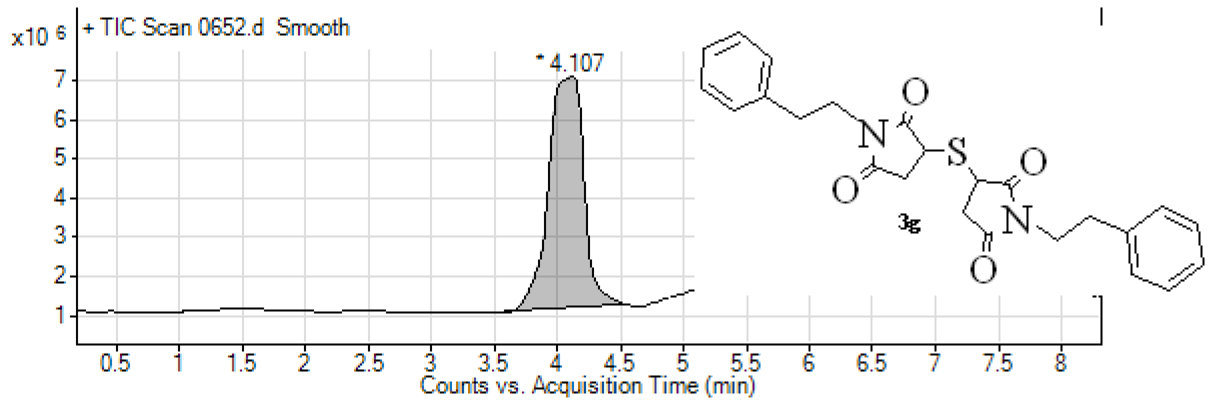

Integration Peak List

Peak	Start	RT	End	Height	Area	Area %
1	3,576	4,107	4,554	5909688,05	116224160,9	100

User Spectra

Fragmentor Voltage	Collision Energy	Ionization Mode
185	0	ESI

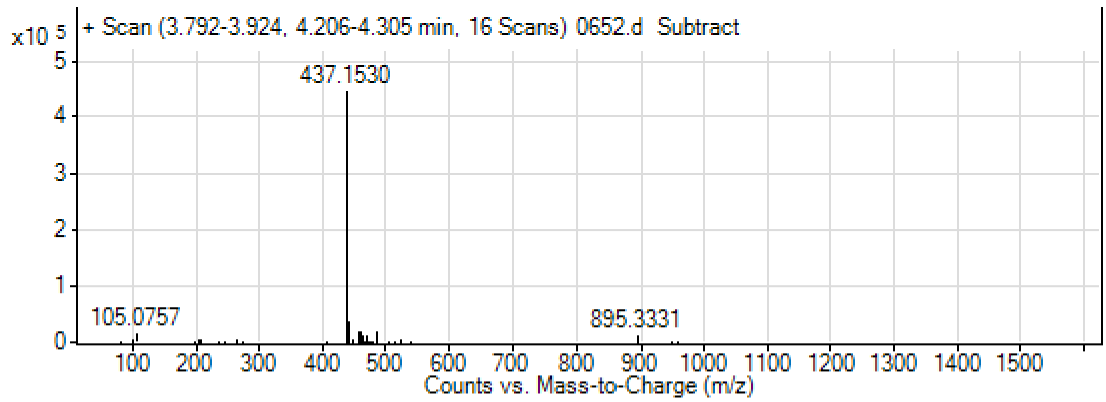

Figure S53: Data HPLC of compound **3g**

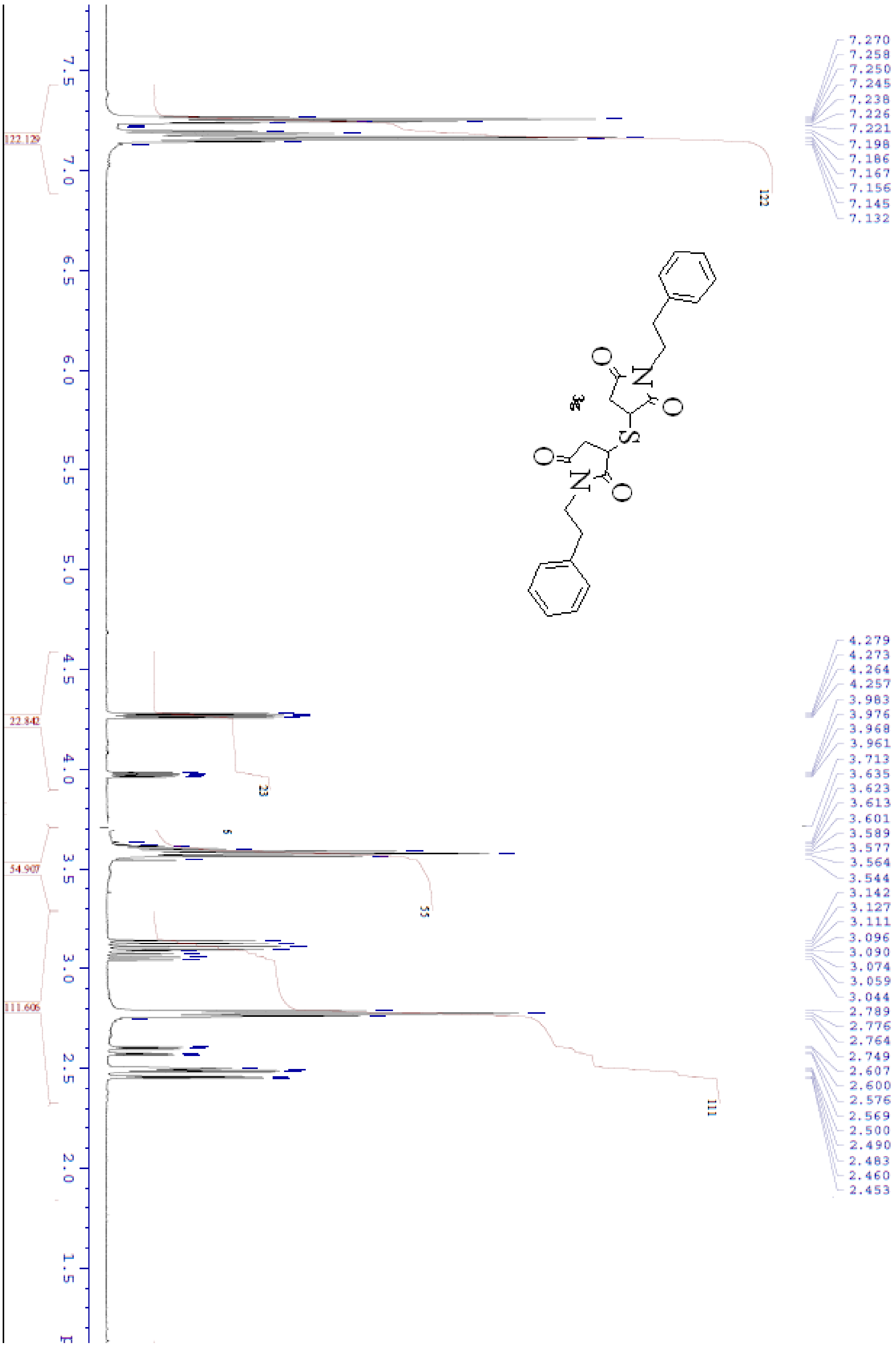

Figure S54: ^{1}H NMR spectrum (500 MHz, DMSO-d6) of compound **3g**

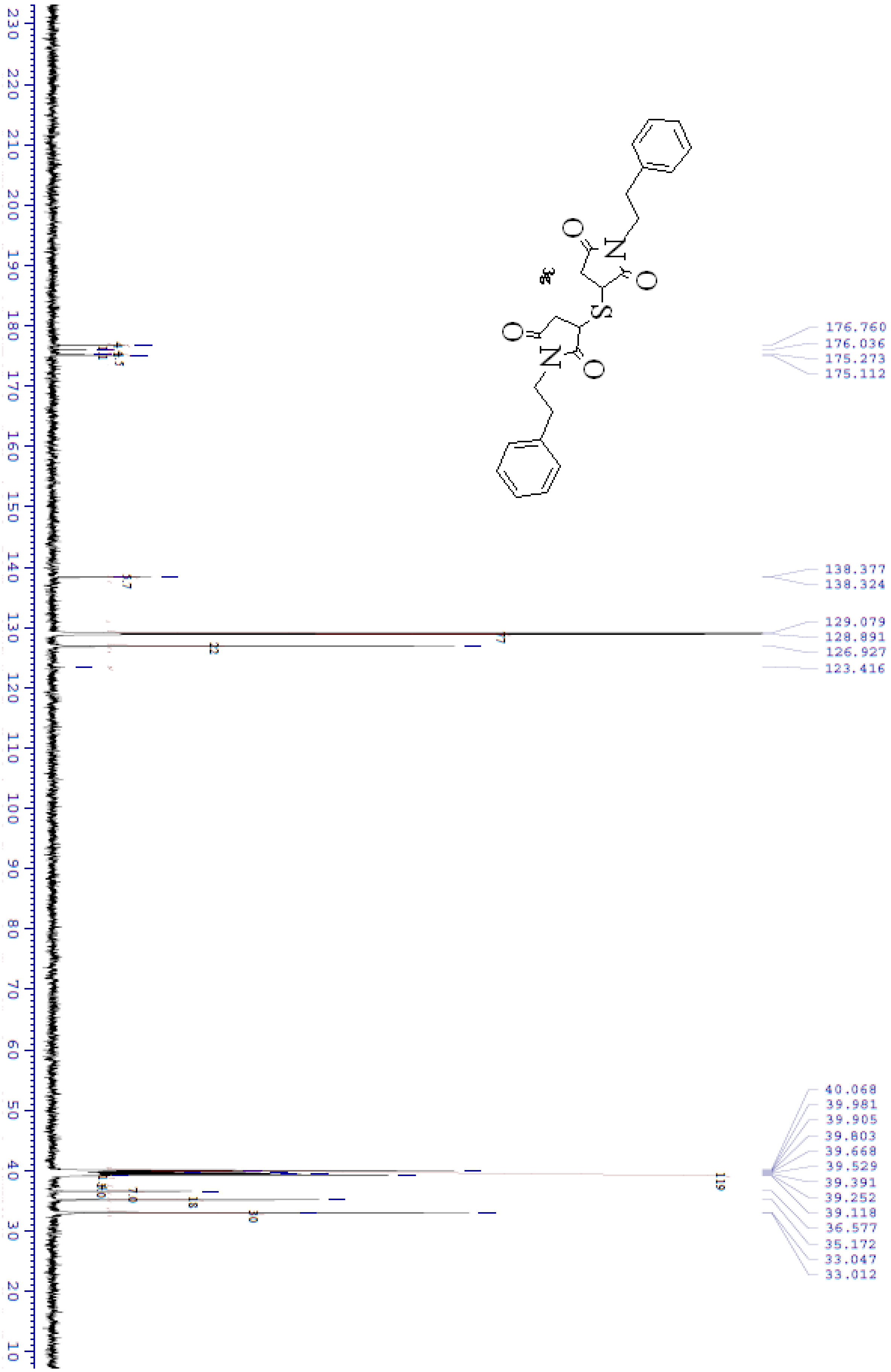

Figure S55: ^{13}C NMR spectrum (125 MHz, DMSO-d6) of compound **3g**

User Chromatograms

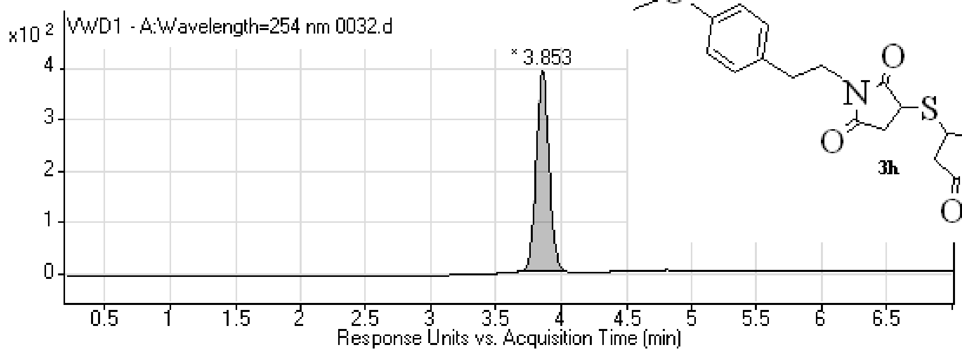

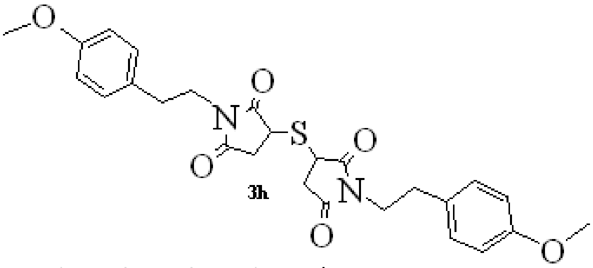

Integration Peak List

Peak	Start	RT	End	Height	Area	Area %
1	3,66	3,853	4,05	391,5	2701,99	100

User Spectra

Fragmentor Voltage	Collision Energy	Ionization Mode
191	0	ESI

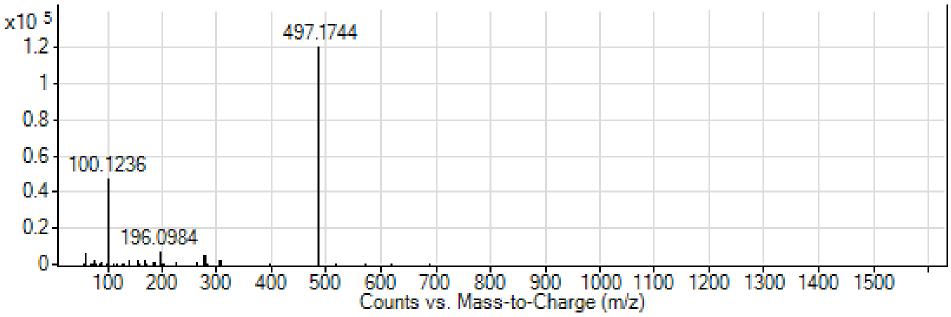

Figure S56: Data HPLC of compound **3h**

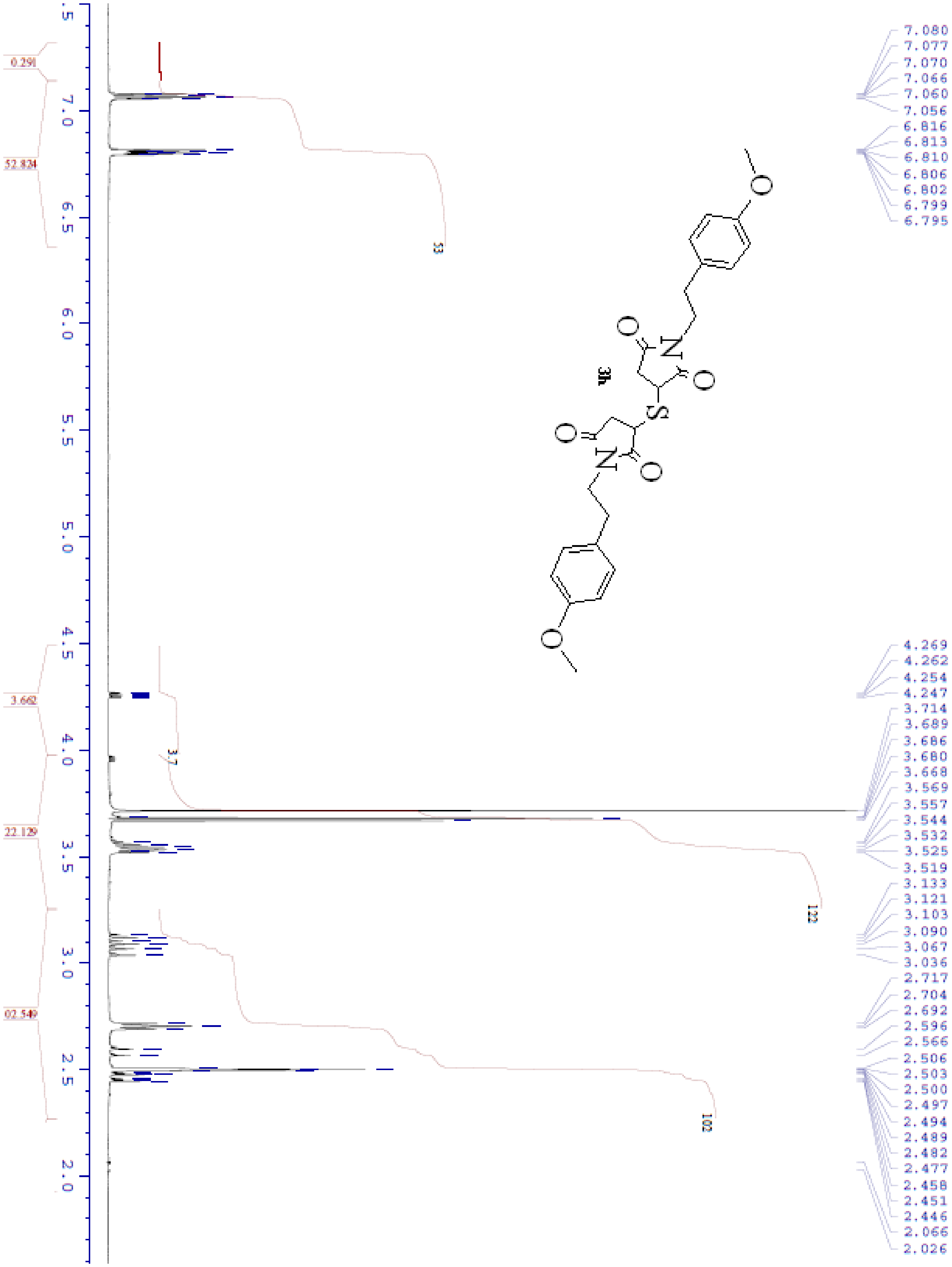

Figure S57: ^{1}H NMR spectrum (500 MHz, DMSO-d6) of compound **3h**

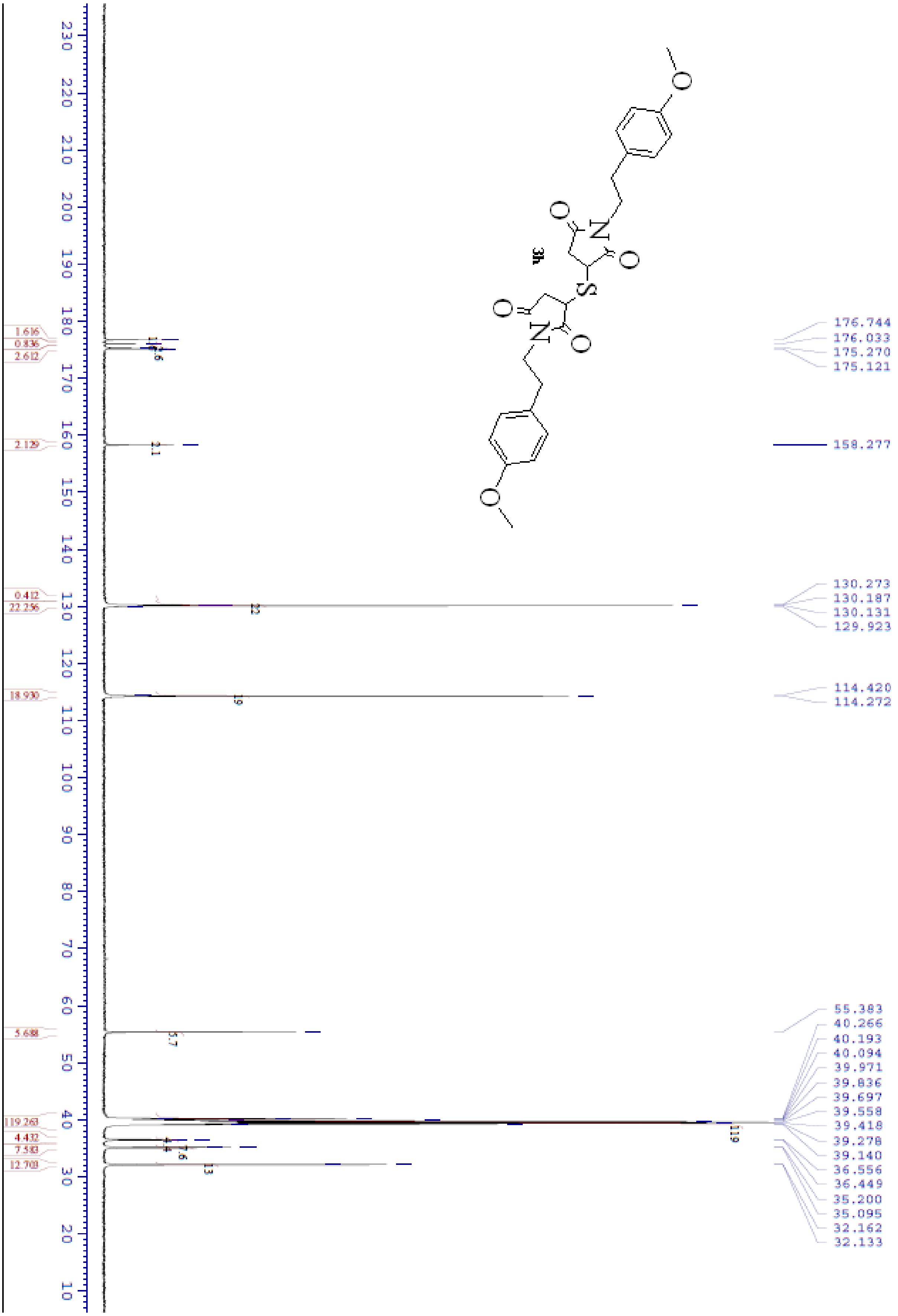

Figure S58: ^{13}C NMR spectrum (125 MHz, DMSO-d6) of compound **3h**
